# Disulfidptosis-related genes define a prognostic signature and novel therapeutic targets in Ewing’s sarcoma through transcriptomic analysis and experimental validation

**DOI:** 10.3389/fimmu.2026.1722729

**Published:** 2026-04-15

**Authors:** Zhenyang Wang, Yongqin Chen, Yuxuan Yang, Lei Qi, Biteng Xu, Ke Li, Liang Wang, Xiejia Jiao

**Affiliations:** 1 Department of Orthopaedics, Qilu Hospital of Shandong University, Jinan, Shandong, China; 2 Department of Orthopaedic, The Second Qilu Hospital of Shandong University, Jinan, Shandong, China

**Keywords:** Keywords: disulfidptosis, disulfidptosis-related genes, Ewing’s sarcoma, immune infiltration, risk model

## Abstract

**Background:**

Ewing’s sarcoma (ES) is a malignant osseous neoplasm characterized by a dismal prognosis, particularly in its metastatic variant. The significance of disulfidptosis—a newly identified cell death mechanism induced by cystine metabolic imbalance and mitochondrial dysfunction—has yet to be investigated in ES. Thus, the aim of this study was to assess the prognostic significance of disulfidptosis-related genes (DRGs) in this disease.

**Methods:**

We analyzed four GEO datasets to examine nine DRGs through differential expression, co-expression networks, and functional enrichment analyses. A predictive risk signature was developed using unsupervised clustering, Cox regression, and LASSO. Drug sensitivity was predicted using the GDSC database, and immune infiltration was quantified by ssGSEA. Single-cell RNA sequencing data was analyzed to explore DRGs distribution and functional heterogeneity. Molecular docking simulations were performed to evaluate interactions between DRGs and chemotherapeutic agents. Experimental validation of key DRGs was conducted in RD-ES cells using PCR and Western blotting, followed by functional studies of *NDUFA11* via siRNA knockdown, assessing proliferation, migration, and invasion.

**Results:**

Eight of nine DRGs were dysregulated in ES tissues. A five-gene risk model (*NDUFS1*, *LRPPRC*, *NDUFA11*, *OXSM*, *NUBPL*) stratified patients into high- and low-risk groups with significantly different survival outcomes. The risk score was an independent prognostic indicator. Drug sensitivity analysis revealed enhanced response to microtubule inhibitors in the low-risk group. Single-cell analysis demonstrated predominant enrichment of these five DRGs in malignant ES cells. *NDUFA11*-high malignant cells exhibited distinct metabolic and signaling pathway enrichment and stronger intercellular communication with the tumor microenvironment. Molecular docking confirmed stable binding between DRGs proteins and chemotherapeutic compounds. Experimental validation confirmed dysregulation of all five DRGs at both RNA and protein levels in RD-ES cells. Functional studies revealed that *NDUFA11* knockdown significantly suppressed ES cell proliferation, migration, and invasion.

**Conclusion:**

This study provides the first prognostic signature connected to disulfidptosis for ES. Functional validation of *NDUFA11* highlights its oncogenic role and potential as a therapeutic target. Single-cell analysis further elucidates the metabolic heterogeneity and microenvironmental interactions underlying ES progression.

## 1 Introduction

Ewing’s sarcoma (ES) is a malignant neoplasm characterized by high aggressiveness, which predominantly affects pediatric and adolescent populations (1). It usually manifests in the pelvis and the diaphysis of long bones (2). There are approximately 2.9 cases per million persons annually (3). The overall survival rate after five years for patients with metastatic disease is less than 30% (4), which poses a major threat to patient survival. Disulfidptosis is a form of regulated cell death that is very different from well-known ways like apoptosis, ferroptosis, and cuproptosis. Its unique metabolic regulation system makes it more important in cancer research (5–7).

Disulfidptosis is marked by disulfide stress resulting from excessive intracellular cystine accumulation. It is primarily governed by genes that facilitate cystine transport and regulate cellular energy metabolism. An imbalance in intracellular cystine metabolism is the fundamental mechanism (8). Under glucose deprivation or mitochondrial dysfunction, cystine accumulation triggers protein disulfide bond crosslinking and cell death (9). DRGs such as *LRPPRC* and *NDUFS1* are critical for maintaining mitochondrial homeostasis and redox balance (10, 11), but their roles in ES remain unexplored. Notably, although *SLC7A11* initiates disulfidptosis in other cancers (12–15), its lack of prognostic significance in ES suggests context-dependent roles. Instead, DRGs involved in core energy metabolism emerged as dominant predictors, highlighting that ES progression is more sensitive to mitochondrial metabolic vulnerabilities than cystine transport alone.

Consistent with the Warburg effect (16), ES cells exhibit significantly greater glycolytic activity and lactate production than nonmalignant cells do, while mitochondrial oxidative phosphorylation is suppressed (17–19). We hypothesize that disulfidptosis may influence ES survival given its reliance on metabolic reprogramming, although this requires experimental validation in ES models. We aimed to define the prognostic role of DRGs and the significance of disulfidptosis in ES. Our work, alongside other novel therapeutic strategies such as targeting the replication stress response (20), highlights the ongoing effort to identify vulnerabilities in this aggressive cancer.

## 2 Materials and methods

### 2.1 Data gathering and preprocessing

We sourced the transcriptomic data from the NCBI Gene Expression Omnibus (GEO) (https://www.ncbi.nlm.nih.gov/geo/query/acc.cgi), utilizing the GEOquery package for data acquisition and curation. The datasets utilized were GSE17674, GSE17618, GSE63155, and GSE63156. The GSE17674 dataset contains transcriptomic and clinical data for 44 ES tumor samples, along with RNA-sequencing data for 18 healthy skeletal muscle samples; in comparison, GSE17618 provides transcriptomic and clinical feature data for 44 ES samples. The datasets GSE17674 and GSE17618 were merged as the training dataset. GSE63155 with transcriptomic and clinical information for 46 ES samples, and GSE63156 with transcriptomic and clinical information for 39 ES samples were used as validation datasets. Based on a previous literature search (8), a total of nine DRGs expressed in ES samples were obtained.

### 2.2 Landscape of DRGs in ES

Differential expression of DRGs between tumor and normal tissues was assessed using the Wilcoxon rank-sum test. The R package igraph with the Pearson method was used to explore the correlations between all the DRGs, which are shown in a graph. Furthermore, the RCircos package was used to visualize the genomic positions of DRGs on the chromosomes. To investigate gene-gene relationships, we employed the corrplot package for visualization. Gene Ontology (GO) and Kyoto Encyclopedia of Genes and Genomes (KEGG) enrichment analyses were performed using the R packages scatterpie and org.Hs.eg.db. To further assess the activity of DRGs in metabolic reprogramming pathways, including oxidative phosphorylation and cystine metabolism, we performed a Gene Set Variation Analysis (GSVA).

### 2.3 Construction of the disulfidptosis-related risk score

We used univariate Cox regression analysis with the survival R package to look at the link between prognostic DRGs and overall survival. To control for potential confounding effects from clinical covariates, we subsequently incorporated age, sex, and disease stage as adjustment variables in a multivariate Cox regression model.

A prognostic signature was developed via LASSO regression. Using the coefficients generated by this algorithm, an individual risk score was calculated per patient. The optimal threshold for categorizing patients into high-risk and low-risk subgroups was then identified using the surv_cutpoint function from the R package survminer. The same risk score formula was employed to calculate risk scores in independent datasets for external validation.

### 2.4 Molecular cluster construction

To determine whether DRGs could define distinct molecular subtypes in ES, we performed unsupervised clustering on the PR-DRGs expression matrix using the ConsensusClusterPlus algorithm to identify distinct molecular patterns (21). This method made it easier to investigate DRGs’ role in ES. Additionally, we used PCA to confirm that these molecular groupings were distinct. We used Kaplan-Meier curves to examine survival differences among the molecular clusters that were discovered. The limma package was then used to compare the expression profiles of DRGs among the three molecularly defined clusters. Finally, we analyzed tumor immune infiltration to characterize the tumor microenvironment states within these three clusters.

### 2.5 Immune microenvironment and drug sensitivity analysis

We used the GSVA package to find enrichment scores of 28 immune cell groups and 13 canonical immunological pathways. This computational method for analyzing the tumor immune milieu is extensively utilized in cancer research and has proved pivotal in uncovering the immunosuppressive conditions linked to unfavorable outcomes (22). Reference gene sets were acquired from the ImmPort database, including standardized signatures for T-cell activation, macrophage polarization, and NK cell function. Differential cell subpopulations were identified using the Wilcoxon rank-sum test, with gene sets sourced from the ImmPort database.

Drug sensitivity prediction: We integrated GDSC2 drug response data using the pRRophetic package. Only clinically relevant anticancer agents were analyzed, including platinum compounds, kinase inhibitors, and microtubule-targeting drugs. The half-maximal inhibitory concentration (IC50), which was calculated from fitted dose-response curves, was used to measure drug sensitivity. Significant associations between DRGs and chemotherapeutic drugs were identified through Spearman correlation, and gene–drug interaction networks were visualized using circlize.

### 2.6 Molecular docking analysis

To investigate potential direct interactions between disulfidptosis-related proteins and small-molecule compounds, molecular docking simulations were performed. The three-dimensional structures of the five prognostic DRGs were obtained from the RCSB Protein Data Bank (PDB; https://www.rcsb.org/) where experimentally resolved structures were available. For proteins lacking crystallographic data, high-confidence structural predictions were retrieved from the AlphaFold Protein Structure Database (AF-DB; https://alphafold.ebi.ac.uk/). The chemical structures of candidate compounds were downloaded from the PubChem database (https://pubchem.ncbi.nlm.nih.gov/).

All protein and ligand structures were prepared using PDBfixer and AutoDockTools. Specifically, crystallographic water molecules and non-protein ligands were removed, polar hydrogen atoms were added, and Gasteiger charges were assigned prior to conversion to PDBQT format. Docking simulations were conducted using AutoDock Vina with a whole-protein surface search box and an exhaustiveness parameter of 32. For each protein-ligand pair, the conformation exhibiting the lowest binding energy was selected as the optimal docking pose.

Intermolecular interactions within the predicted complexes were characterized using the Protein–Ligand Interaction Profiler (PLIP; https://plip-tool.biotec.tu-dresden.de/) and the Protein Plus server (https://proteins.plus/), which generate detailed 2D and 3D interaction diagrams illustrating hydrogen bonds, hydrophobic contacts, π-stacking, and other non-covalent interactions.

### 2.7 Single-cell RNA sequencing data processing and analysis

Single-cell transcriptomic data of ES were obtained from the GEO database (GSE277083). Tumor samples were selected for analysis. The Seurat package was used for data processing. After quality control, data were normalized and scaled. Batch effects were corrected using Harmony. Principal component analysis was performed, and the top principal components were used for graph-based clustering and UMAP visualization. Cell types were manually annotated based on marker genes from the literature (23).

For functional investigation, ES tumor cells were stratified into NDUFA11 high and low subgroups based on median expression. Differential expression analysis was performed using the Wilcoxon test. Enrichment analysis of GO terms and KEGG pathways was conducted using clusterProfiler. Intercellular communication networks were reconstructed and compared between the two subgroups using the CellChat package.

### 2.8 Validation of hub DRGs by RT-PCR and Western blotting

#### 2.8.1 Cell culture

RD-ES cells, a human Ewing sarcoma cell line, were acquired from the American Type Culture Collection (ATCC), while Cyagen (Guangzhou, China) supplied mesenchymal stem cells (MSCs).

#### 2.8.2 Real-time PCR

Using Trizol reagent (Sigma, USA), we got total RNA from cells that had been grown in culture. Then, a commercial kit (Takara, Japan) was used to turn RNA into complementary DNA (cDNA). We performed quantitative real-time PCR (qPCR) using SYBR Premix Ex Taq (Takara, Japan) according to the instructions from the company. **Table 1** shows all of the primer sequences that were made using the PrimerBank database. We used the 2^-△△CT^ method to calculate relative gene expression levels. Every experiment was conducted in a minimum of three independent replicates.

**Table 1:** Sequences of the primers used for RT–PCR validation.

Gene symbol	Forward (5’ to 3’)	Reverse (5’ to 3’)
*LRPPRC*	ATCATGGCGGAGAGATTGGC	AAATCGGGGTTTGTTCAGCA
*NDUFA11*	GCCTACAGCACCACCAGTAT	GTCCAACCTTAGCCACTCCT
*OXSM*	CTCACCTGGTTTGGGATCGT	TTGGCACATAAGCAGCAACAC
*NDUFS1*	GGAAGAACCCTCCCAAGGTG	TGGCAAATCCTGTCGTGTGA
*NUBPL*	TGTGATTGTCTCCACGCCC	GTTTCCTTGCACCATCAGCAC

#### 2.8.3 Western blot analysis

In order to isolate the total protein, protease inhibitors (Biosharp, BL507A) were incorporated into RIPA lysate buffer (Wuhan Bioruler, BF0003). Protein samples were separated on 10% SDS-PAGE gels (Wuhan Bioruler, BF0006) and electrophoretically transferred to PVDF membranes (Baitaibio, W8040) at 300 mA for 1.5 hours. 5% skim milk (Biosharp, BS102) was used to block the membranes for 30 minutes in TBST buffer (Wuhan Bioruler, B0065). The membranes were incubated overnight at 4°C with the following primary antibodies: anti-NDUFS1 (DF7041; 1:1000), anti-OXSM (16642-1-AP; 1:1000), anti-LRPPRC (21175-1-AP; 1:1000), anti-NDUFA11 (17879-1-AP; 1:1000), anti-NUBPL (ER1914-17; 1:1000), and anti-GAPDH (abcam, ab9485; 1:1000). After rinsing, the membranes were kept at room temperature for 30 minutes with an HRP-conjugated goat anti-rabbit secondary antibody (Jackson, 111-035-003; 1:5000). An ECL substrate (Wuhan Bioruler, BF0023) was used to visualize the protein bands, and Image-Pro Plus software was used to quantify them.

### 2.9 Functional validation of NDUFA11 in RD-ES cells

#### 2.9.1 siRNA transfection

Small interfering RNAs (siRNAs) targeting *NDUFA11* and negative control siRNAs (si-NC) were designed and synthesized by GenePharma (Shanghai, China). RD-ES cells were seeded in 6-well plates at a density of 2 × 10^5^ cells per well and cultured overnight to reach 60–70% confluence. Transfection was performed using Lipofectamine 3000 (Invitrogen, USA) according to the manufacturer’s protocol. The final concentration of siRNA was 50 nM. After 48 h of transfection, knockdown efficiency was assessed by RT-qPCR and Western blotting.

#### 2.9.2 Colony formation assay

RD-ES cells were seeded into 6-well plates at a density of 1x10^3^ cells per well. Cells were cultured for 10–14 days in complete medium until visible colonies formed. Colonies were fixed with 4% paraformaldehyde for 20 min and stained with 0.1% crystal violet for 15 min. Colonies containing more than 50 cells were counted under a microscope. Each experiment was performed in triplicate.

#### 2.9.3 Wound healing assay

RD-ES cells were seeded into 6-well plates and cultured to full confluence. A sterile 200 μL pipette tip was used to create a linear scratch across the cell monolayer. Debris was removed by washing with PBS, and cells were cultured in serum-free medium for 24 h. Wound closure was observed and photographed at 0 h and 24 h using an inverted microscope.

#### 2.9.4 Transwell migration and invasion assays

Cell migration and invasion were assessed using 24-well Transwell chambers with 8 μm pore size polycarbonate membrane inserts (Corning, USA). For migration assays, 1×10^4^ RD-ES cells suspended in 200 μL serum-free medium were seeded into the upper chamber. The lower chamber was filled with 600 μL medium containing 10% FBS as a chemoattractant. For invasion assays, the upper chambers were pre-coated with Matrigel (Corning, USA) diluted in serum-free medium. After incubation for 24 h at 37 °C, cells remaining on the upper surface were removed with a cotton swab. Migrated or invaded cells on the lower surface were fixed with methanol, stained with 0.1% crystal violet, and photographed under an inverted microscope. Five random fields per well were counted. Each assay was performed in triplicate.

### 2.10 Statistics and visualization

We used the statistical program R version 4.4.3 (https://www.r-project.org/) for all of the analyses, and we set the significance level at *p* < 0.05. Spearman’s rank correlation was used to look at relationships between variables, and the Wilcoxon rank-sum test was used to compare groups.

## 3 Results

### 3.1 Landscape of DRGs in ES

**Figure 1:**
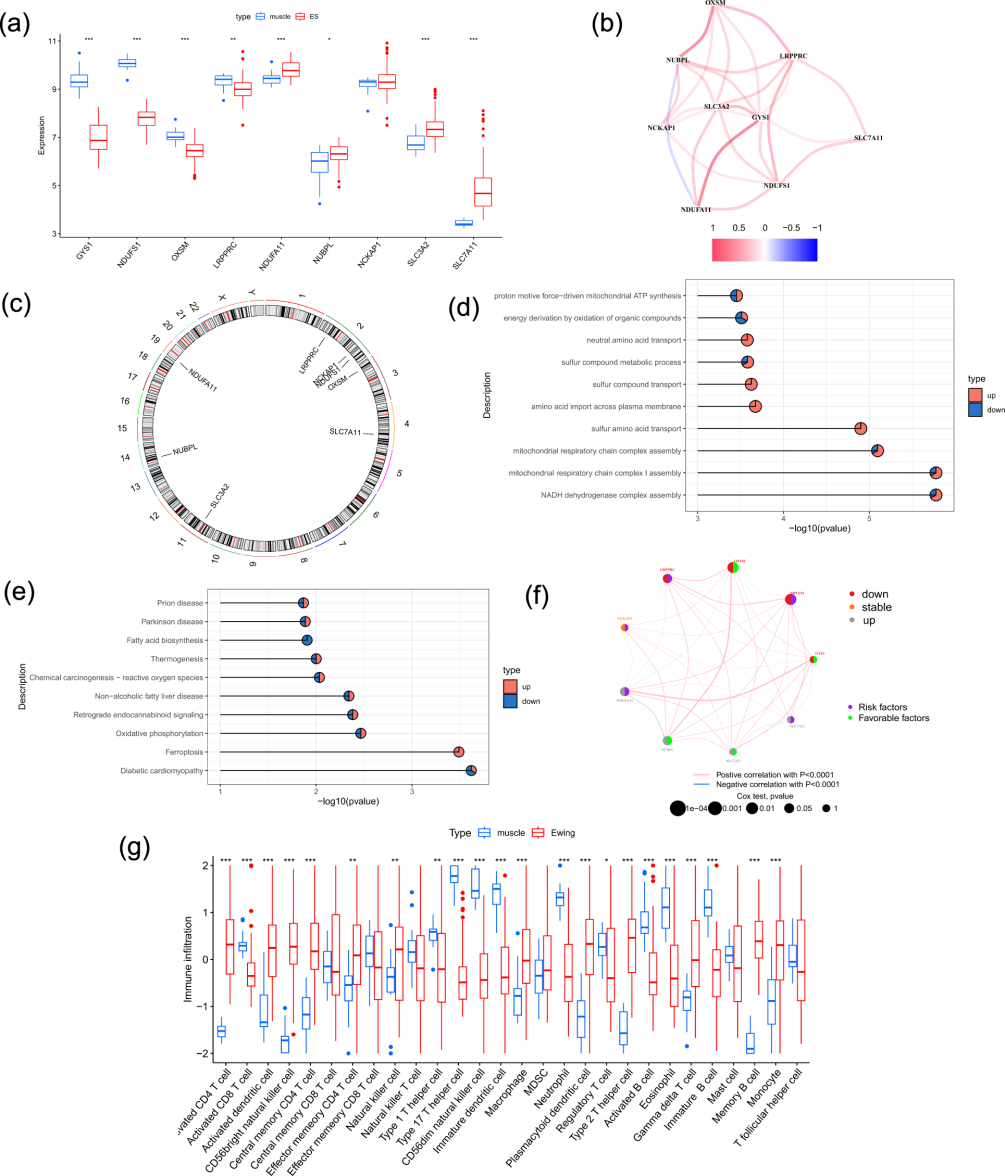
DRGs expression landscape and functional enrichment in Ewing’s Sarcoma. **(a)** Boxplot illustrating differences in the expression of nine DRGs between tumor and healthy muscle tissues (*p < 0.05; **p < 0.01; ***p < 0.001; ns, not significant). **(b)** A Pearson correlation coefficient-based co-expression network analysis of the nine DRGs that shows both negative (blue edges) and positive (red edges) correlations. **(c)** An RCircos map that indicates the locations of the nine DRGs on the chromosomes and suggesting possible clustering. **(d)** The findings of a GO enrichment analysis for biological processes that are intimately associated with the DRGs are displayed in a bar plot. **(e)** Enriched KEGG pathways for DRGs, illustrated using a bar plot. **(f)** The interaction of the DRGs. **(g)** Immune cell infiltration profile in ES tumor versus normal muscle tissue, quantified by ssGSEA enrichment scores (*p < 0.05, **p < 0.01, ***p < 0.001; ns, not significant). **(h)** Immune pathway activity comparison between ES and normal muscle tissues, based on ssGSEA scores.

Initially, differential gene expression analysis was used to investigate nine DRGs associated with the disease within the training dataset. Our analysis showed that DRGs were significantly differentially expressed in tumor and normal tissues that matched. In particular, *NDUFA11*, *NUBPL*, *SLC3A2*, and *SLC7A11* presented increased expression, whereas *GYS1*, *NDUFS1*, *OXSM*, and *LRPPRC* presented decreased expression (**Figure 1a**).

We used Co-expression network analysis to construct a network with nine nodes. The findings revealed that *NDUFA11* and *GYS1*, as well as *NUBPL* and *OXSM*, exhibited strong positive associations, whereas *NUBPL* and *NDUFA11* showed a substantial negative correlation (**Figure 1b**). Additionally, a cluster on chr2p16.1 that includes *LRPPRC*, *NCKAP1*, and *NDUFS1* was found by chromosomal localization analysis, suggesting potential genomic colocalization (**Figure 1c**).

Significant correlations between DRGs and amino acid metabolism and mitochondrial bioenergetics were found by GO enrichment analysis. Sulfur amino acid transport, NADH dehydrogenase complex assembly, mitochondrial complex I assembly, and mitochondrial respiratory chain complex assembly were the biological activities with the highest levels of enrichment (**Figure 1d**). This finding implies that DRGs play a vital role in oxidative phosphorylation within the mitochondria. Additionally, the findings demonstrated a downregulation of sulfur amino acid transport, potentially supporting the high proliferative state in tumor tissues by conserving energy for biosynthesis. Oxidative phosphorylation, retrograde endocannabinoid signaling, diabetic cardiomyopathy, non-alcoholic fatty liver disease, and ferroptosis were the most enriched pathways, according to KEGG pathway analysis, which also revealed that DRGs were enriched in pathways linked to cellular redox and metabolic stress (**Figure 1e**). These results indicate that DRGs balance a metabolic program of high energy production and stress resilience, which is of great significance for maintaining high proliferative activity in tumors (24, 25). The analysis of the gene regulatory network elucidates the interaction profiles of DRGs in ES (**Figure 1f**). To examine immune infiltration in ES, we performed ssGSEA to compare ES tumor tissues and control tissues. Most immune cell types were differentially abundant in tumor and normal tissues (**Figure 1g**).

### 3.2 Identification of consensus clusters, survival analysis, and immune infiltration

**Figure 2:**
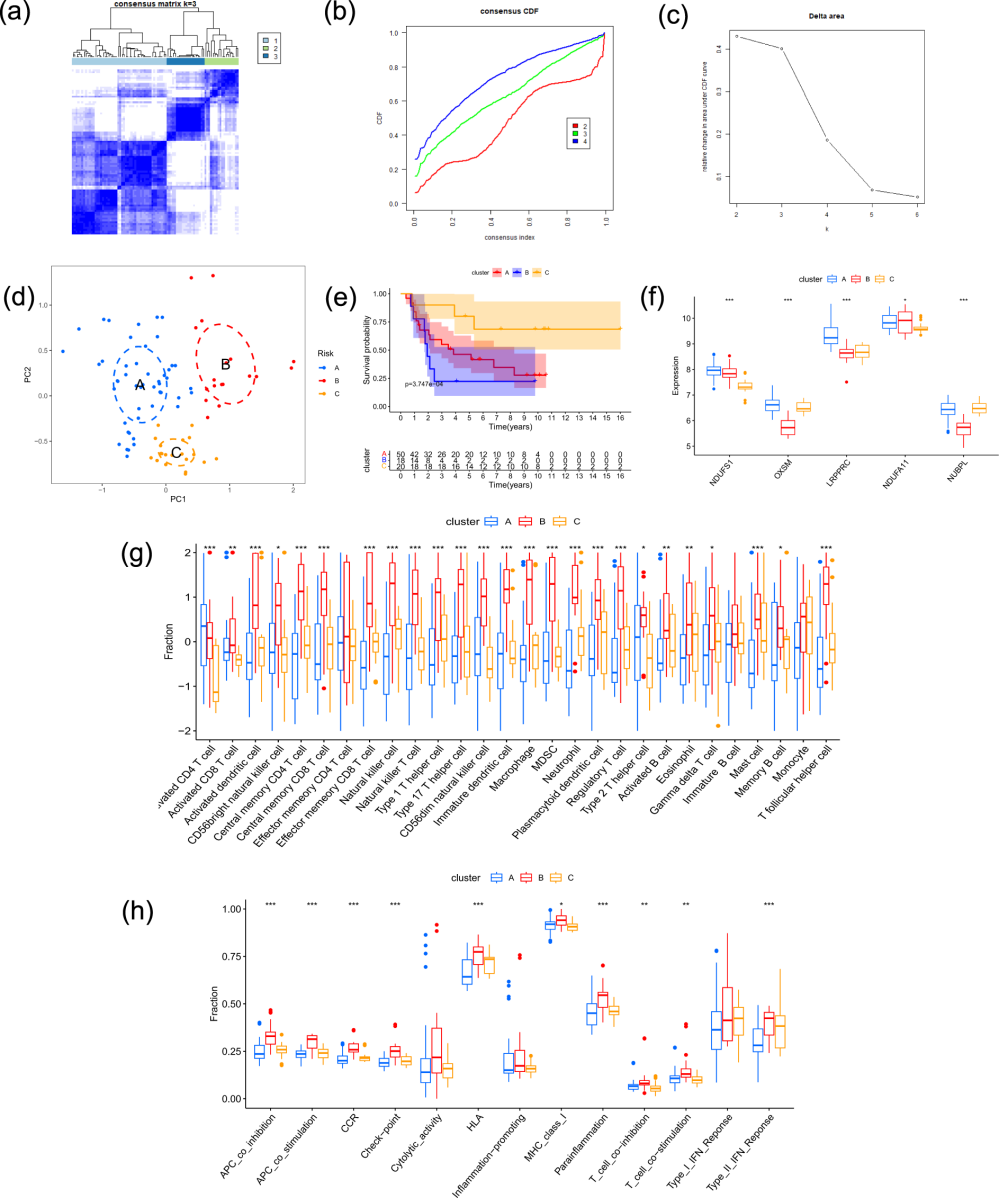
Identification of molecular clusters based on DRGs expression and their clinical correlations. **(a–c)** Consensus clustering analysis identified three distinct molecular subtypes in the training dataset based on the expression of PR-DRGs. **(d)** A PCA plot confirming the three molecular clusters’ separation. **(e)** Kaplan-Meier survival curves demonstrating that the three molecular clusters’ overall survival differs significantly. **(f)** Boxplots displaying the three molecular clusters’ expression levels of the five PR-DRGs (*p < 0.05; **p < 0.01; ***p < 0.001; ns, not significant). **(g)** The three molecular clusters’ variations in immune cell infiltration are depicted in a heatmap. **(h)** Boxplots illustrating how the three molecular clusters differ in immune-related pathway activity.

To identify unique patterns, unsupervised clustering analysis based on DRGs was carried out. We used the consensus clustering approach to estimate the optimal number of clusters. As a result, three clusters were identified from the ES samples in the training dataset: cluster A, cluster B, and cluster C (**Figures 2a–c**). PCA was subsequently employed to show that these clusters were clearly separated from one another (**Figure 2d**). Kaplan-Meier analysis was employed to evaluate prognostic differences between the patient clusters. The results revealed notable variations in survival rates (**Figure 2e**), with cluster C showing a longer survival duration. Additionally, we examined the differences in DRGs expression among these three clusters, as shown by box plots, which revealed differences in DRGs expression across the three clusters. The expression of DRGs varied significantly among the three clusters (**Figure 2f**). In particular, *NDUFA11* exhibited a substantial difference, whereas *NDUFS1*, *OXSM*, *LRPPRC*, and *NUBPL* exhibited highly significant differences. The majority of immune cell signatures in cluster B exhibited higher enrichment scores in ssGSEA than in cluster A, suggesting a an association between DRGs expression patterns and immune microenvironment characteristics (**Figure 2g**). Furthermore, additional immune pathway analysis revealed that cluster B exhibited significantly greater enrichment than clusters A and C in multiple aspects, including APC co-stimulation, APC co-inhibition, HLA, CCR, checkpoint and parainflammation (**Figure 2h**).

### 3.3 Prognosis-related DRGs in ES

**Figure 3:**
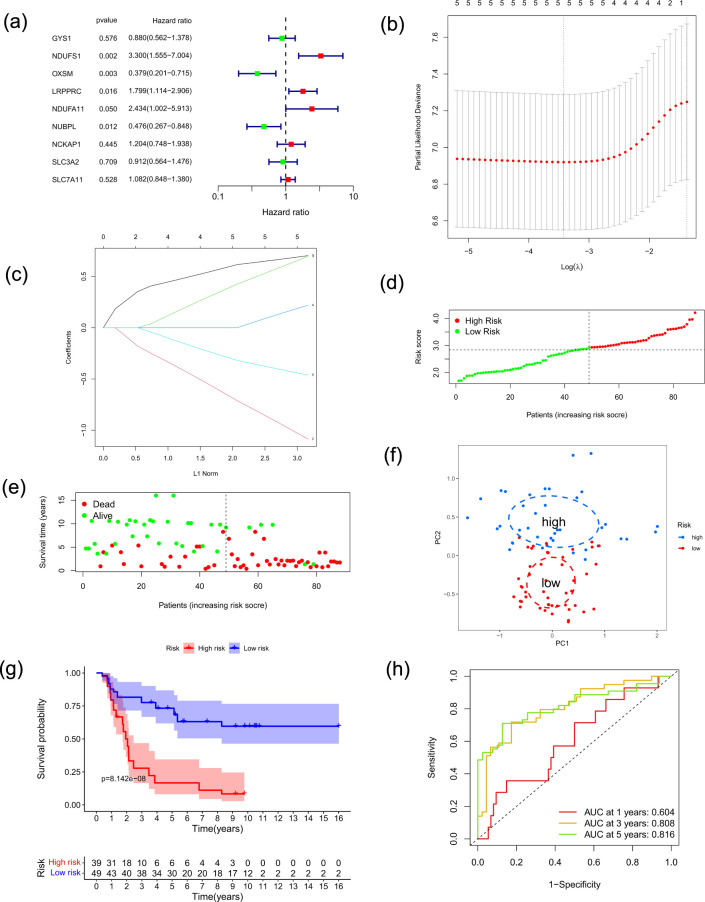
Construction and validation of a PR-DRGs signature. **(a)** Forest plot of the five PR-DRGs selected through univariate Cox regression analysis for model construction. **(b)** The LASSO coefficient profiles for the five PR-DRGs. **(c)** Choosing the best penalty parameter (λ) for the LASSO regression, which is done through ten-fold cross-validation. **(d)** Distribution of patient risk scores in the training group. **(e)** A scatter plot showing how higher risk scores are linked to shorter patient survival times. **(f)** The five-PR-DRGs signature effectively discriminated between high- and low-risk patients in the PCA. **(g)** The Kaplan–Meier analysis showed that there was a significant difference in overall survival between the risk-stratified groups (Log-rank *p* = 8.142×10^-1^). **(h)** Time-dependent ROC curves evaluating the risk model’s precision in forecasting 1-year, 3-year, and 5-year overall survival, accompanied by the respective AUC values.

For each gene, we employed univariate Cox analysis to determine hazard ratios (HR) and p values. Five DRGs with *p* < 0.05 were then chosen as prognosis-related DRGs (PR-DRGs), and forest plots were generated to display the findings. According to the analysis, *OXSM* and *NUBPL* had HR < 1, indicating a better prognosis, but *NDUFS1*, *LRPPRC*, and *NDUFA11* had HR > 1, indicating a worse prognosis (**Figures 3a, b**). These five genes (*NDUFS1*, *LRPPRC*, *NDUFA11*, *OXSM*, and *NUBPL*) were subsequently identified as predictive signatures via LASSO (**Figure 3c**).

Subsequently, we implemented the surv_cutpoint function to divide the ES samples comprising the training dataset into two risk categories. Patients classified in the high-risk group demonstrated a markedly inferior prognosis relative to those in the low-risk group, as evidenced by the survival status and risk score analysis (**Figures 3d, e**). Principal component analysis (PCA) provided additional verification of these results, as evidenced by the clear separation between groups (**Figure 3f**). Kaplan-Meier survival analysis (**Figure 3g**) disclosed substantial disparities in survival rates among the risk categories. Additionally, the risk model’s sensitivity and specificity were verified by time-dependent ROC curves, demonstrating high prognostic accuracy for overall survival at 1-year, 3-year, and 5-year (**Figure 3h**).

**Figure 4:**
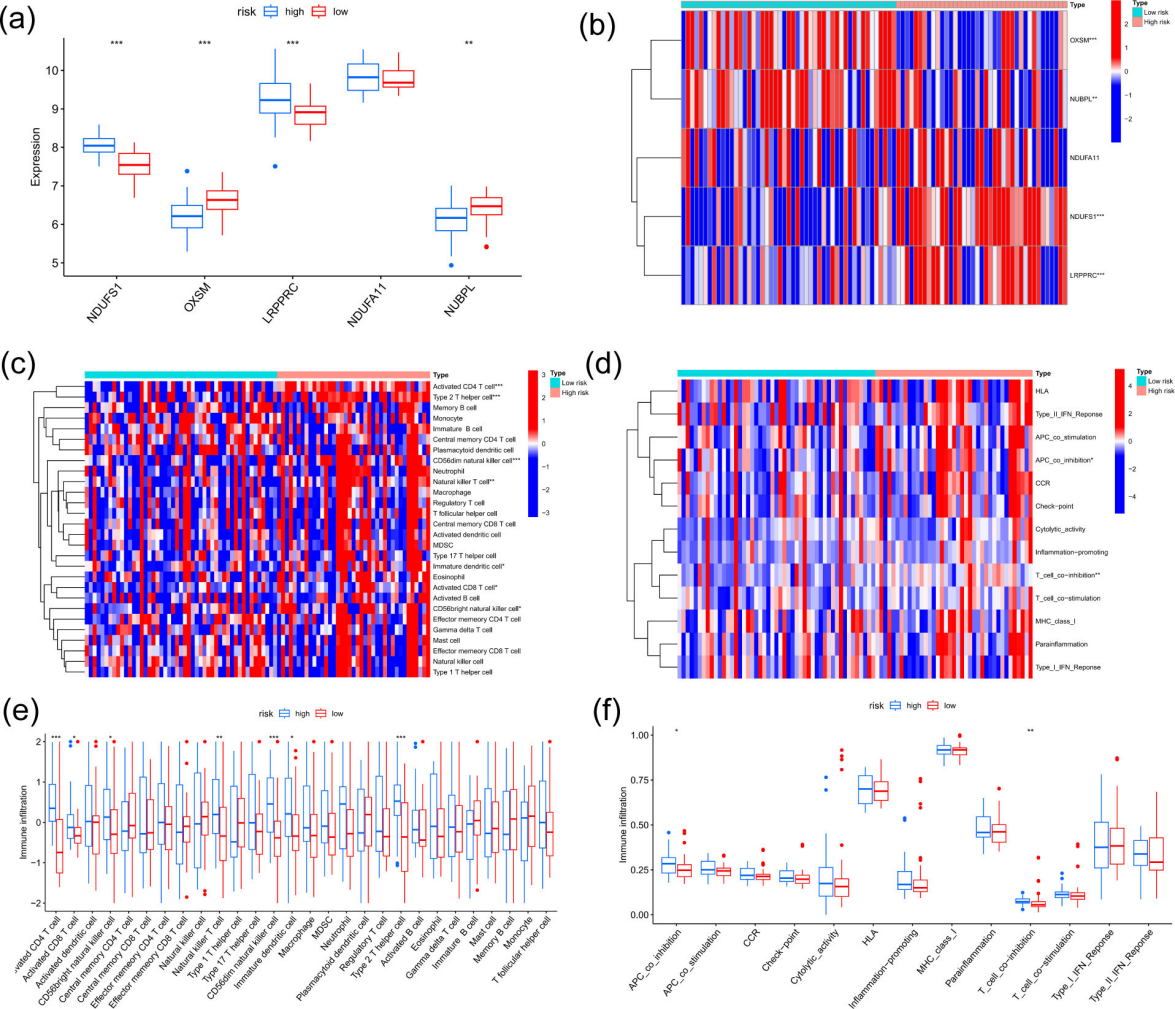
Characteristics of the high-risk and low-risk datasets in the training dataset. **(a)** Five PR-DRGs were expressed differently in high-risk and low-risk groups (*p < 0.05; **p < 0.01; ***p < 0.001; ns, not significant). **(b)** A composite heatmap that incorporates the five PR-DRGs’ expression patterns, continuous risk scores, and patient survival status. **(c)** The landscape of immune cell infiltration across risk groups, as measured by enrichment scores. **(d)** Activity of immune-related pathways in high-risk and low-risk groups. **(e)** Immune cell infiltration abundance across risk-stratified groups. **(f)** Differential activity of immune-related pathways between high-risk and low-risk groups.

To visualize the distinct signature profiles between the two groups, we generated a heatmap and a box plot (**Figures 4a, b**). In order to investigate the immunological mechanisms and prognostic stratification in ES, we employed ssGSEA to compare immune-related pathway activity and immune cell infiltration characteristics between the low-risk and high-risk groups. This was necessary due to the substantial impact of the immune microenvironment on tumor behavior. The high-risk group exhibited a substantial enrichment of natural killer T cells, CD56bright natural killer cells, CD56dim natural killer cells, Th2 cells, activated CD4 T cells, and iDCs (**Figures 4c, e**). Simultaneously, this group exhibited substantially increased activity in the T-cell and APC coinhibitory pathways (**Figures 4d, f**).

### 3.4 Validation of the risk model

**Figure 5:**
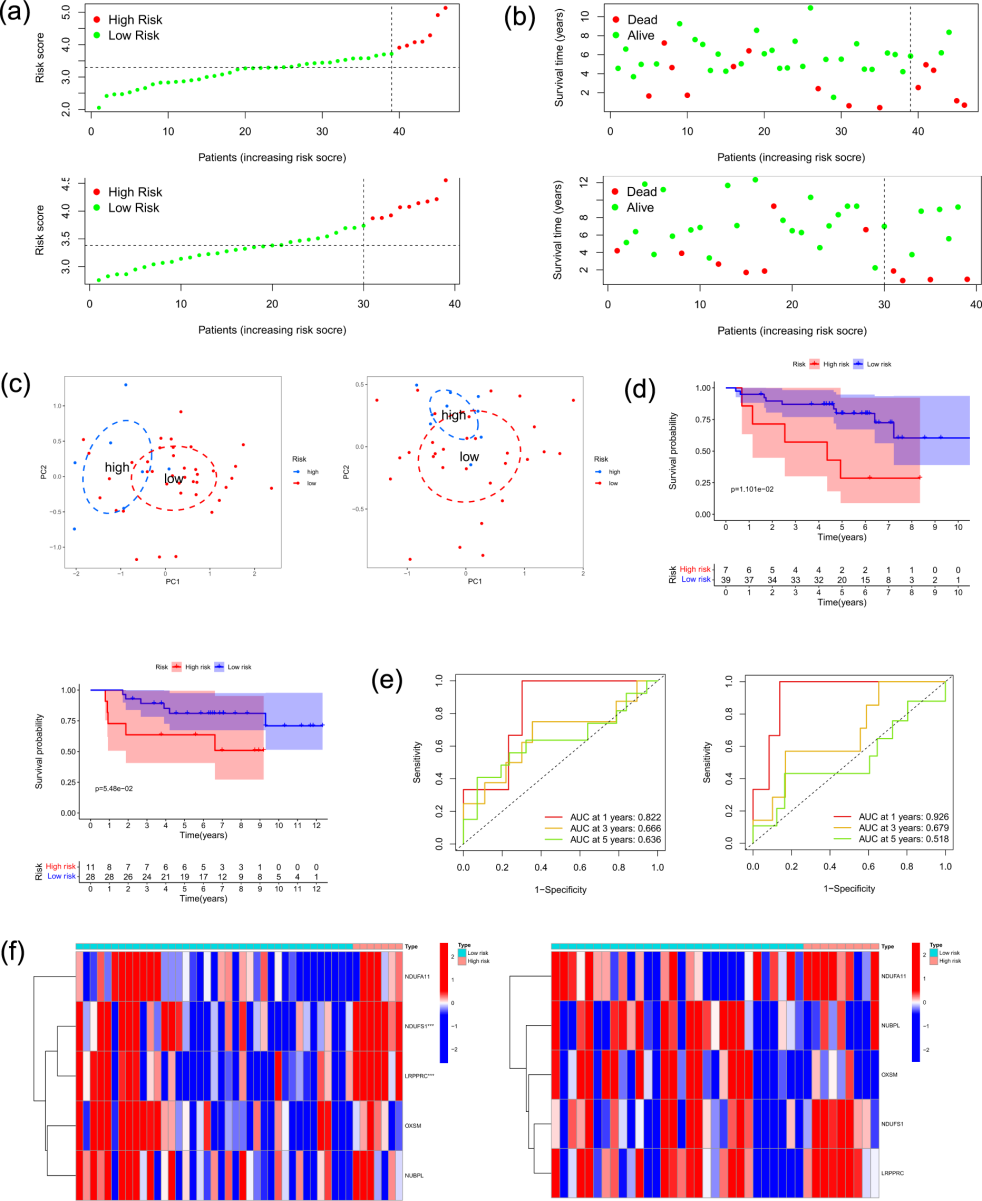
Validation of the Prognostic Risk Model in External Datasets GSE63155 and GSE63156. **(a)** Distribution of patient risk scores in validation datasets GSE63155 and GSE63156. **(b)** Scatter plots showing the association between continuous risk scores and survival status in GSE63155 and GSE63156. **(c)** PCA plots demonstrating separation between high-risk and low-risk groups based on the 5-PR-DRGs signature in GSE63155 and GSE63156. **(d)** Kaplan–Meier survival curves for GSE63155 and GSE63156. **(e)** Time-dependent ROC curves evaluating the predictive accuracy of the risk model for 1-year, 3-year, and 5-year overall survival in GSE63155 and GSE63156. **(f)** Heatmaps displaying differential expression of the five prognostic genes between high-risk and low-risk patients in GSE63155 and GSE63156.

We used two external datasets (GSE63155 and GSE63156) to validate the accuracy of our risk prediction model. Visualizations of risk scores and survival status confirmed that high-risk patients had worse prognosis (**Figures 5a, b**). PCA further supported distinct separation between risk groups (**Figure 5c**). Time-dependent ROC curves demonstrated the model’s discriminative ability (**Figure 5d**). Kaplan–Meier analysis indicated significantly poorer survival for high-risk patients in GSE63155, and a consistent though statistically non-significant trend in GSE63156, possibly due to its smaller sample size (**Figure 5d**). Nonetheless, the model exhibited robust predictive performance in both independent datasets, with consistently high AUC values at 1-year, 3-year, and 5-year intervals (**Figure 5e**). Heatmaps illustrated expression patterns of the five prognostic genes across risk groups (**Figure 5f**).

### 3.5 Clinical features linked to the risk model

**Figure 6:**
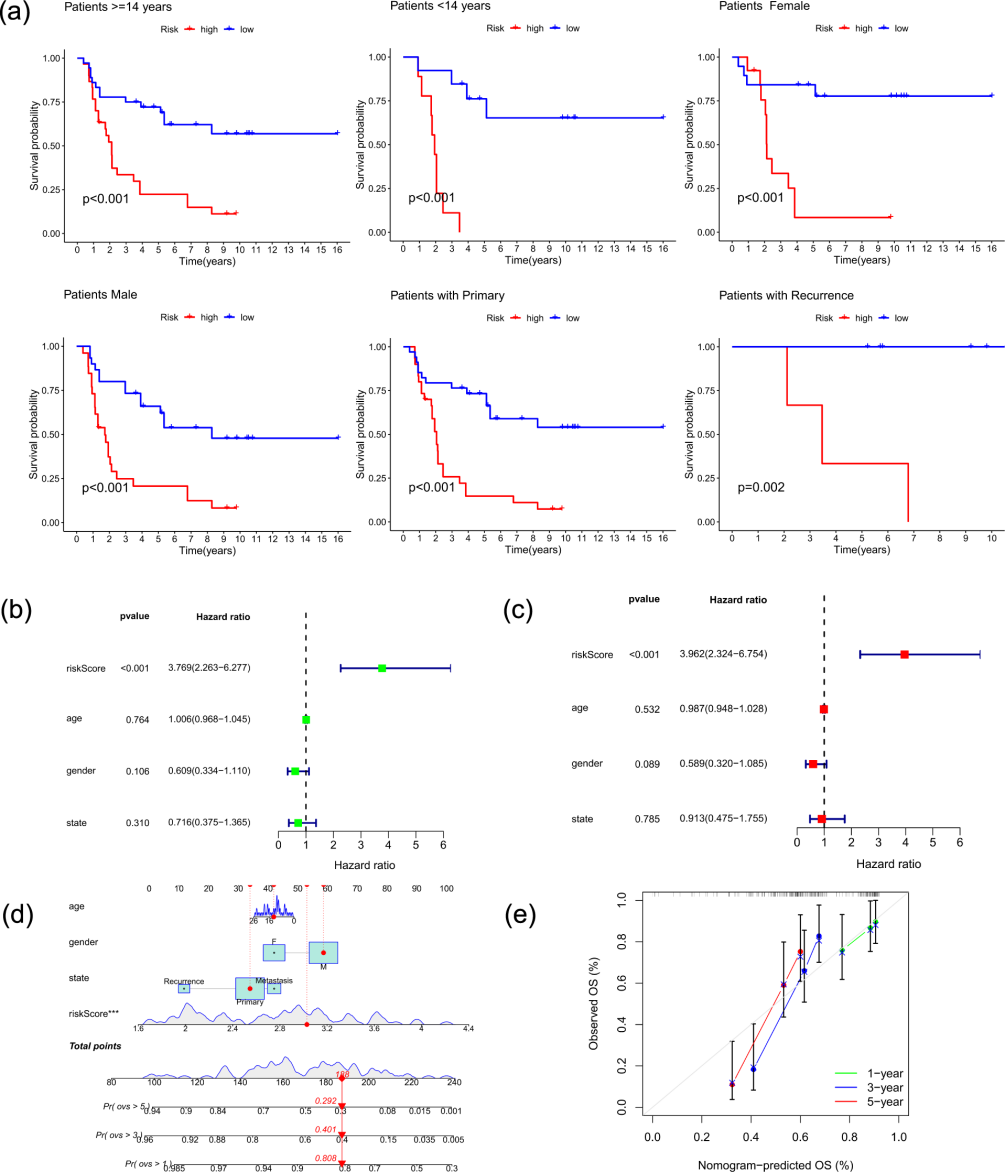
Prognostic stratification and nomogram validation. **(a)**. Kaplan–Meier survival analysis of the risk model in different clinical subgroups, including age, sex, and disease status. **(b)** Univariate Cox regression analysis evaluating the association of the risk score and clinical features with overall survival. **(c)** Multivariate Cox regression analysis confirming the independent prognostic value of the risk score after adjusting for clinical covariates. **(d)** Nomogram integrating the risk score and clinical variables for predicting 1-, 3-, and 5-year overall survival. **(e)** Calibration plots showing the agreement between nomogram-predicted and observed survival probabilities at 1, 3, and 5 years.

Kaplan-Meier survival analysis was used o validate that the risk model effectively predicted outcomes. The findings showed that the risk model successfully differentiated patient outcomes across a number of categories (**Figure 6a**). Notably, significant separation was observed in the age-stratified groups and sex subgroups. It also effectively distinguished outcomes in patients in the primary stage and recurrent stage.

### 3.6 Nomogram construction and validation

We first evaluated the prognostic performance of the risk signature across clinical subgroups. Kaplan–Meier analysis showed that the risk model effectively stratified patients by age, sex, and disease status (**Figure 6a**). Univariate Cox regression identified the risk score as a significant prognostic factor (**Figure 6b**). After adjusting for clinical covariates, multivariate analysis confirmed that the risk score remained an independent predictor (*p* < 0.001), whereas clinical characteristics were not significant (**Figure 6c**). A nomogram integrating the risk score and clinical factors was constructed to predict 1-, 3-, and 5-year overall survival (**Figure 6d**). Calibration plots demonstrated good agreement between predicted and observed survival probabilities (**Figure 6e**).

### 3.7 Immune infiltration analysis in the training dataset

**Figure 7:**
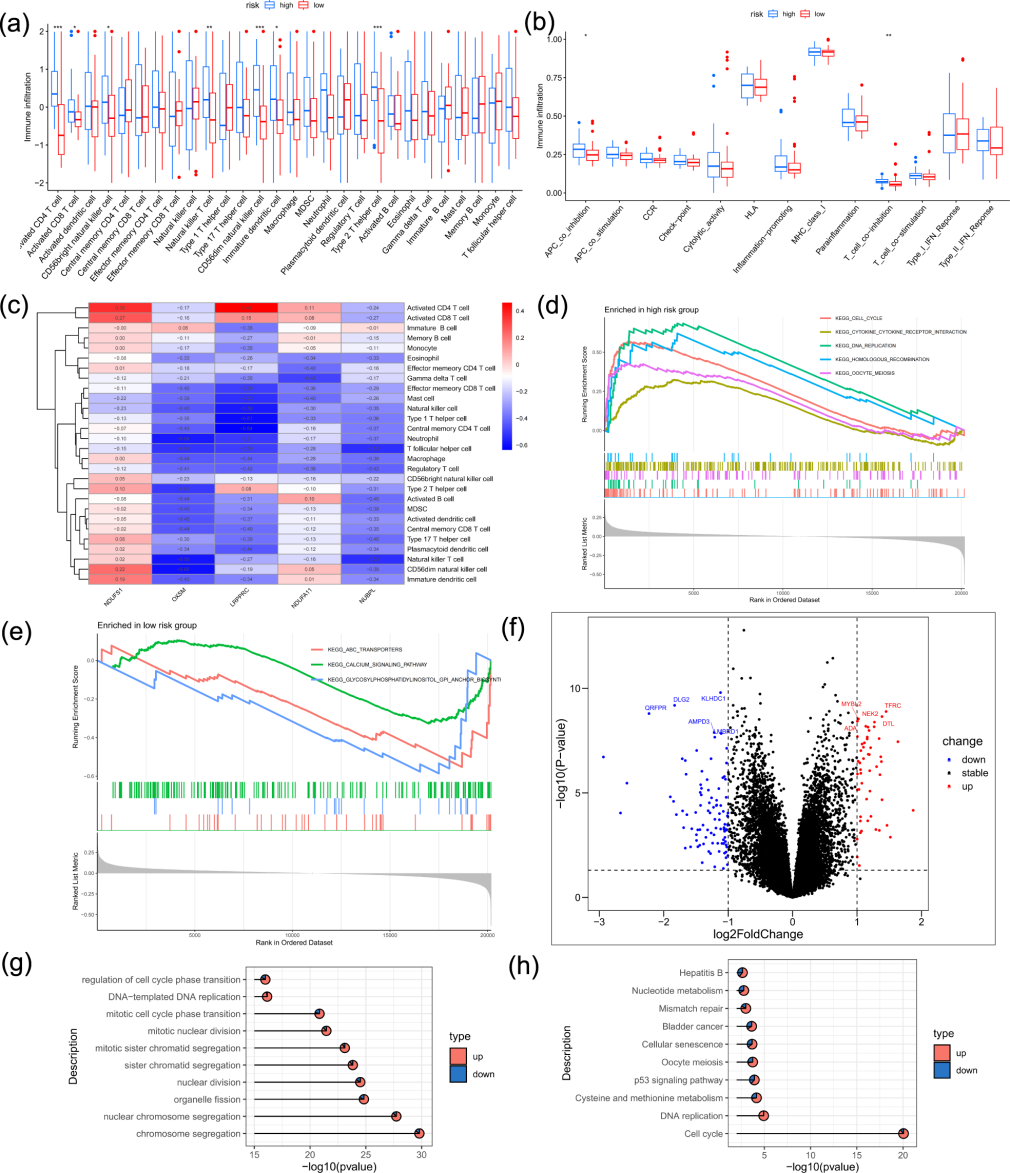
Tumor immune microenvironment and functional enrichment across risk groups. **(a)** Assessment of immune cell infiltration levels comparing high-risk and low-risk subgroups within the training dataset. Significance codes: *p < 0.05, **p < 0.01, ***p < 0.001; ns, not significant. **(b)** Expression patterns of major immune checkpoint molecules stratified by risk group. **(c)** Diagram illustrating the five PR-DRGs included in the prognostic signature. **(d)** GSEA results highlighting KEGG pathways significantly activated in high-risk patients. **(e)** Enrichment profiles of KEGG pathways markedly upregulated in the low-risk subgroup. **(f)** Volcano plot of DEGs between high-risk and low-risk groups. g-h. Bar plots displaying the top significantly enriched KEGG pathways and GO Biological Process terms.

Based on the prognostic risk scores, the training dataset was categorized into high-risk and low-risk subgroups. We then applied ssGSEA to examine variations in immune microenvironment features and immune-related pathway activation between these subgroups (**Figures 7a, b**).

Clustering analysis based on DRG expression levels identified distinct immune infiltration patterns associated with the five prognostic genes (**Figure 7c**). The genes in the low-risk group were enriched in pathways such as the calcium signaling pathway, ABC transporters, and glycosylphosphatidylinositol anchor biosynthesis, whereas those in the high-risk group were enriched in pathways related to the cell cycle, cytokine–cytokine receptor interactions, homologous recombination, DNA replication, and oocyte meiosis (**Figures 7d, e**).

### 3.8 Functional enrichment of differentially expressed genes between risk groups

Differential gene expression between high-risk and low-risk groups was visualized in a volcano plot (**Figure 7f**). Pathway enrichment analysis showed that the DEGs were predominantly linked to processes such as chromosome segregation, mitotic sister chromatid separation, nuclear division, mitotic nuclear division, nuclear chromosome segregation, and the transition between mitotic and cytoplasmic cell cycle phases (**Figure 7g**). Moreover, cellular functional enrichment analysis indicated a substantial role of these genes in the cell cycle (**Figure 7h**).

### 3.9 Chemotherapeutic drug sensitivity

**Figure 8:**
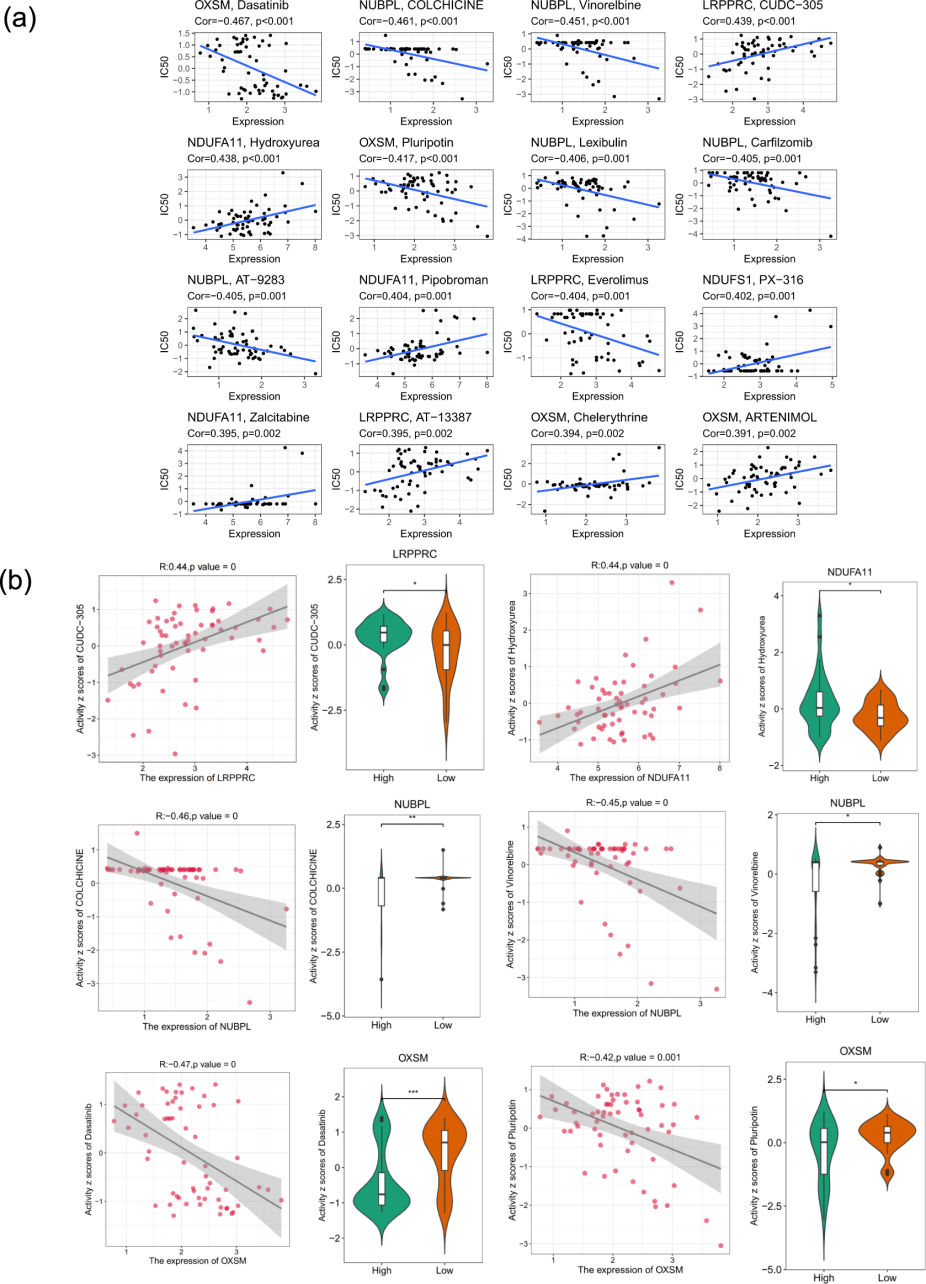
Prognostic DRGs expression and chemotherapeutic drug sensitivity are linked. **(a)** Circos plot illustrating the gene-drug interaction network between the 5 prognostic DRGs and sensitivity (IC50 values) to various chemotherapeutic agents. For important interactions, significant p-values and Spearman correlation coefficients are displayed. **(b)** Violin plots with inner boxplots showed that the predicted IC50 values for targeted agents and microtubule inhibitors were much lower in the low-risk group than in the high-risk group (p < 0.001). *p < 0.05, **p < 0.01, ***p < 0.001.

The drug–gene interaction network revealed significant correlations between five prognostic DRGs and specific chemotherapeutic agents (**Figure 8a**). IC50-based group comparisons confirmed significantly greater drug sensitivity to microtubule inhibitors and targeted agents in the group with a low risk than in the group with a high risk (**Figure 8b**).

### 3.10 Molecular docking

**Figure 9:**
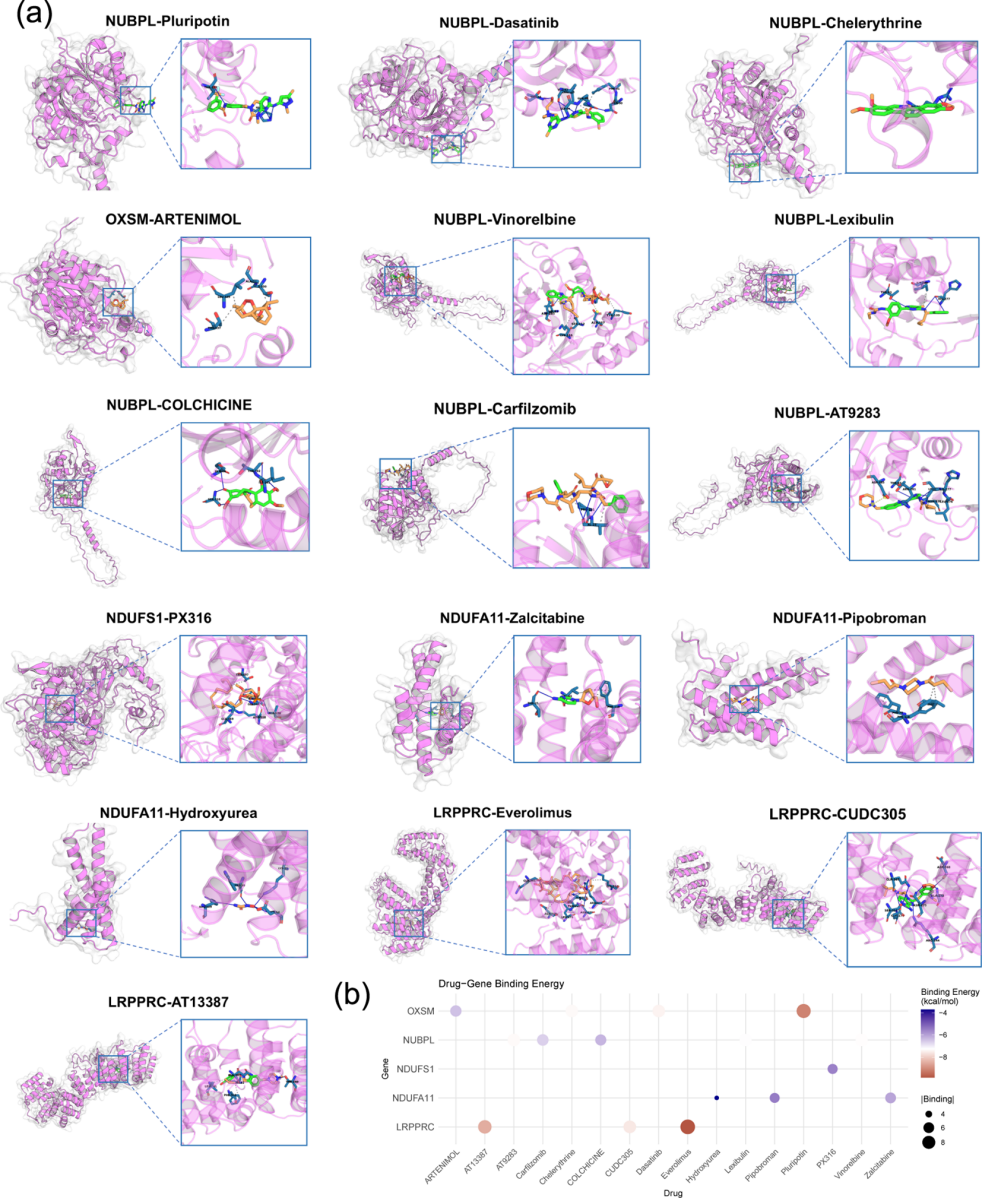
Molecular docking analysis. **(a)** Visualization of representative docking poses. **(b)** Dot plot summarizing docking scores for the five DRGs with chemotherapeutic compounds.

To evaluate the potential binding between the five prognostic DRGs and chemotherapeutic agents, molecular docking was performed. All five proteins exhibited stable binding with multiple compounds, indicating favorable interactions (**Figures 9a, b**).

### 3.11 Single−cell transcriptomic landscape of ES

**Figure 10:**
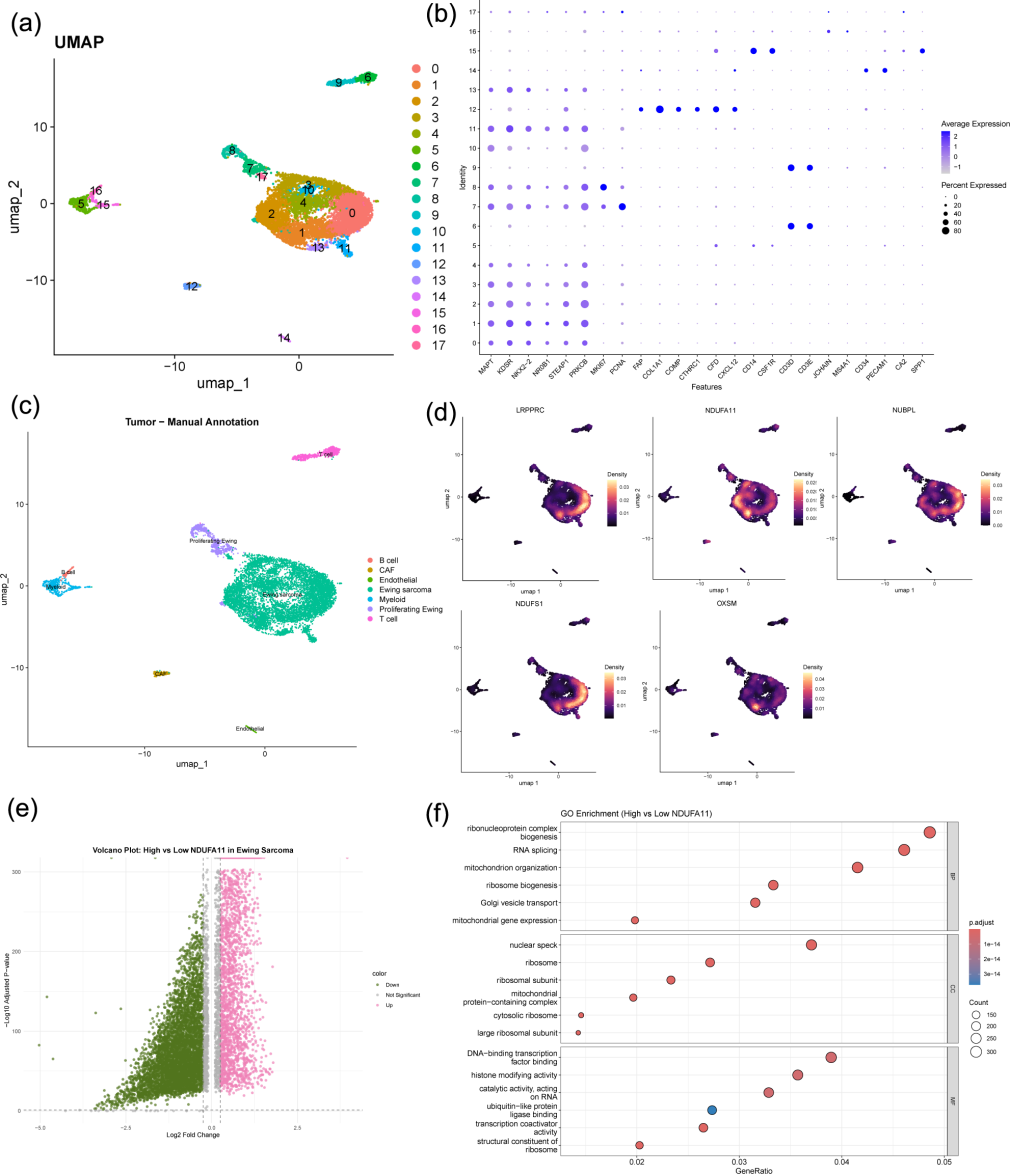
Single−cell transcriptomic landscape of Ewing sarcoma. **(a–c)** UMAP visualization and marker gene expression patterns used for cell type annotation. **(d)** Enrichment of five disulfidptosis–related prognostic genes in the malignant ES cell compartment. **(e)** Volcano plot of differentially expressed genes between NDUFA11-high and -low subgroups. **(f)** GO enrichment analysis of DEGs.

Single−cell analysis of ES tumor samples revealed diverse cell populations, including malignant ES cells, T cells, myeloid cells, cancer−associated fibroblasts (CAFs), endothelial cells, and B cells, as defined by marker genes. The UMAP visualization and marker gene expression patterns used for annotation are shown (**Figures 10a, b**). The five previously identified disulfidptosis−related prognostic genes were predominantly enriched in the malignant ES cell compartment (**Figure 10c**).

**Figure 11:**
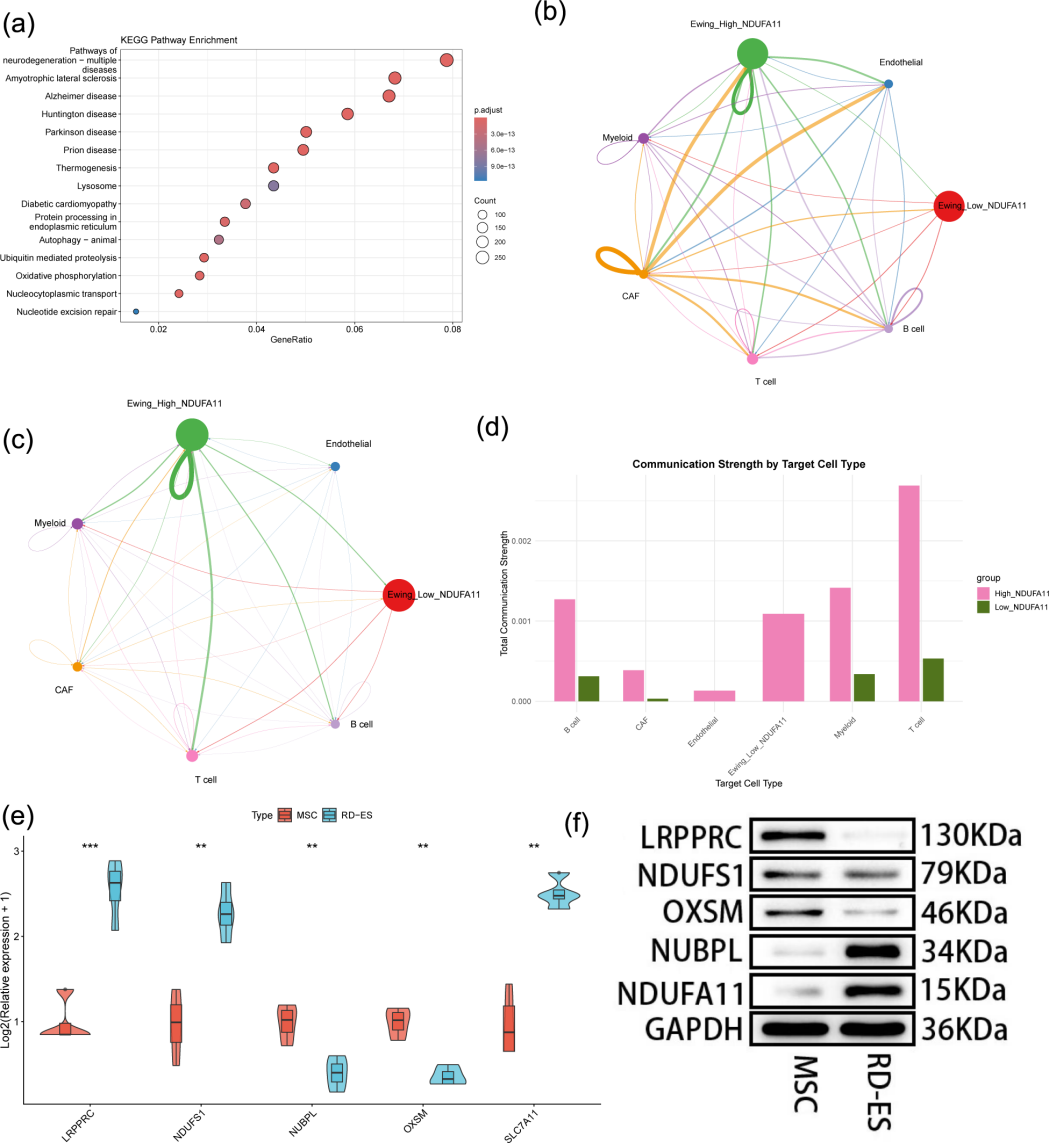
Single−cell functional analysis and experimental validation. **(a)** KEGG pathway enrichment analysis of DEGs between *NDUFA11*-high and -low subgroups. b-d. Cell−cell communication analysis: **(b)** interaction counts; **(c)** interaction weights; **(d)** total interaction strength per cell type. **(e)** RT−qPCR validation of five prognostic DRGs expression levels in RD-ES cells compared to MSCs. **(f)** Western blot validation of five prognostic DRGs protein levels in RD-ES cells compared to MSCs. **p < 0.01, ***p < 0.001.

To explore functional heterogeneity associated with *NDUFA11*, ES cells were dichotomized into high− and low−expression subgroups. Differential expression analysis revealed distinct transcriptional profiles, and a volcano plot of the top DEGs is shown (**Figure 10d**). GO enrichment analysis of DEGs showed significant enrichment in biological processes such as mitochondrial gene expression, ribosome biogenesis, mitochondrion organization, and RNA splicing. Cellular component terms included ribosomal subunit, mitochondrial protein-containing complex, and nuclear speck, while molecular functions involved structural constituent of ribosome, transcription coactivator activity, and ubiquitin-like protein ligase binding (**Figure 10e**). KEGG pathway analysis revealed significant enrichment in oxidative phosphorylation, Ubiquitin mediated proteolysis, Autophagy, Protein processing in endoplasmic reticulum, as well as several neurodegeneration-related pathways (**Figure 11a**).

Intercellular communication analysis using CellChat demonstrated that the *NDUFA11*-high subgroup exhibited stronger interactions with all cell types compared to the low subgroup, including T cells, myeloid cells, CAFs, endothelial cells, and B cells (**Figures 11b–d**). These findings indicate that *NDUFA11* expression level modulates not only the metabolic state of ES cells but also their microenvironmental crosstalk.

### 3.12 Experimental validation of key regulators

RT–qPCR analysis of the Ewing sarcoma cell line RD-ES confirmed significant dysregulation of all five prognostic risk genes compared to MSCs. The expression of *OXSM* and *NUBPL* was significantly decreased, whereas the expression of *LRPPRC*, *NDUFS1*, and *NDUFA11* was significantly increased (**Figure 11e**).

Western blot analysis further validated these findings at the protein level. Consistent with the RNA results, *LRPPRC, NDUFS1, and NDUFA11* presented increased protein abundance, whereas *OXSM* and *NUBPL* presented decreased expression in RD-ES cells (**Figure 11f**).

### 3.13 Functional validation of *NDUFA11* in RD-ES cells

**Figure 12:**
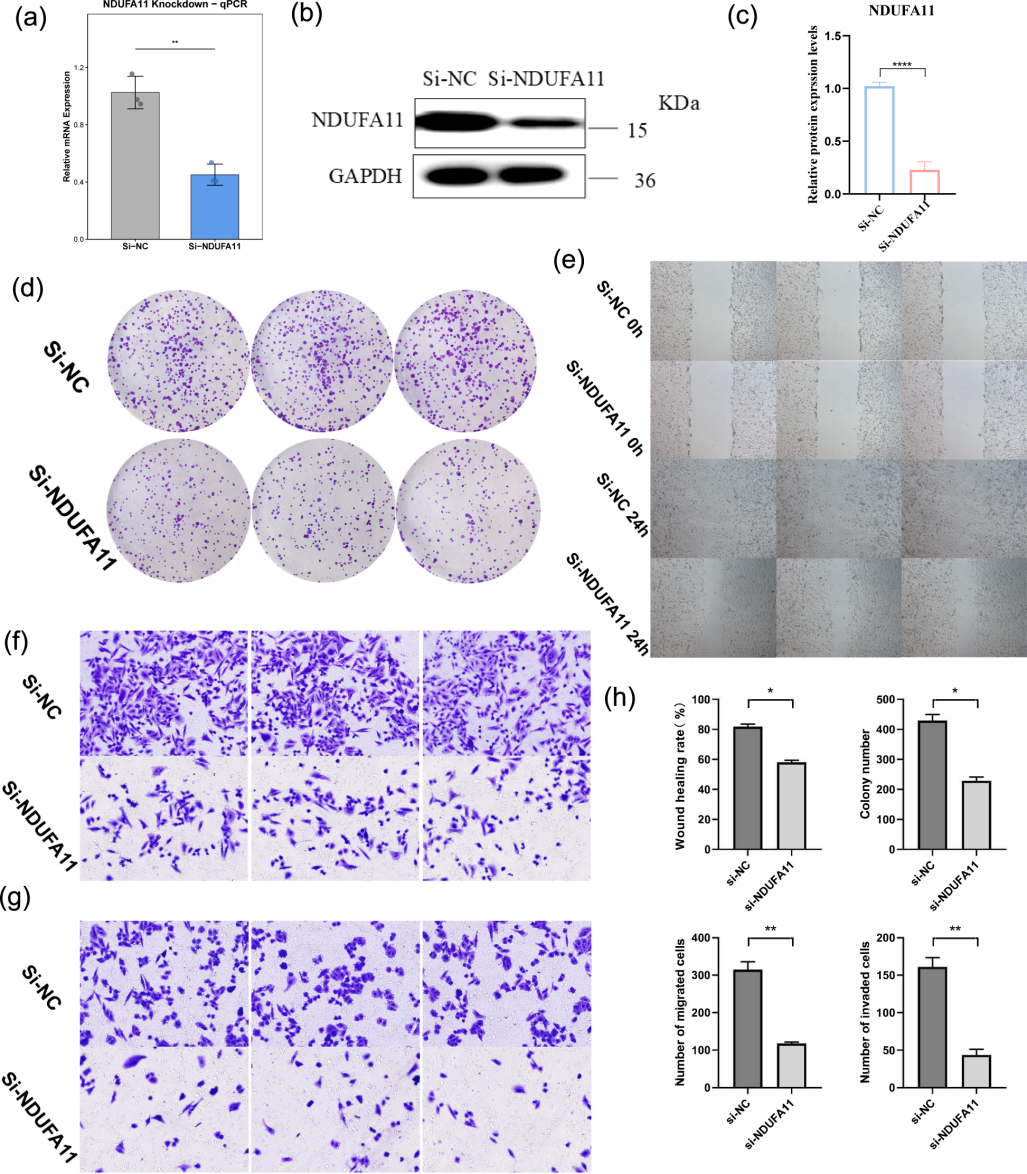
Functional validation of NDUFA11 knockdown in RD-ES cells. **(a)** RT−qPCR confirmation of *NDUFA11* mRNA knockdown efficiency. **(b)** Western blot images of *NDUFA11* protein knockdown. **(c)** Quantification of Western blot results. **(d)** Representative images of colony formation assays. **(e)** Representative images of wound healing assays at 0 h and 24 h. **(f)** Representative images of Transwell migration assays. **(g)** Representative images of Transwell invasion assays. **(h)** Summary bar plot showing quantification of colony formation, wound healing, migration, and invasion assays. *p < 0.05, **p < 0.01, ***p < 0.001.

To further investigate the biological role of *NDUFA11* in Ewing’s sarcoma progression, we performed loss-of-function experiments in RD-ES cells using siRNA-mediated knockdown. RT-qPCR and Western blot analysis confirmed efficient silencing of NDUFA11 at both mRNA and protein levels (**Figures 12a–c**).

Colony formation assays revealed that knockdown of *NDUFA11* significantly suppressed the proliferative capacity of RD-ES cells. The number of colonies formed in the si-*NDUFA11* group was markedly reduced compared to the si-NC group (**Figure 12d**).

Wound healing assays demonstrated that *NDUFA11* depletion impaired cell migration. The wound closure rate at 24 h was significantly lower in si-*NDUFA11*-treated cells than in control cells (**Figure 12e**). Consistently, Transwell migration assays showed that the number of migrated cells per field was substantially decreased upon *NDUFA11* knockdown (**Figure 12f**). Invasion assays further revealed that *NDUFA11* silencing significantly reduced the invasive capacity of RD-ES cells (**Figure 12g**). Quantification of all four functional assays is summarized in **Figure 12h**.

These results indicate that *NDUFA11* promotes both proliferation and migration of ES cells, supporting its oncogenic role and reinforcing its potential as a therapeutic target in Ewing’s sarcoma.

## 4 Discussion

Despite advances in multimodal therapeutic approaches, the prognosis of ES remains unsatisfactory (4). Proto-oncogenes and tumor suppressor genes undergo a spectrum of genetic and epigenetic alterations during ES pathogenesis (26). Disulfidptosis, a novel type of controlled cell death, has recently garnered increasing attention. It differs mechanistically from classical apoptosis, ferroptosis, and cuproptosis and is brought on by intracellular cystine metabolic imbalance and disulfide stress (8). According to earlier research, focusing on pathways linked to disulfidptosis may inhibit cancer cell proliferation and survival (19). Furthermore, a number of treatment approaches targeting important molecules or metabolic nodes implicated in disulfidptosis are currently under development (9). This study intends to thoroughly examine the expression patterns, prognostic significance, and possible therapeutic implications of DRGs in ES in light of the growing role of disulfidptosis in cancer metabolic reprogramming.

This study is the first transcriptomic analysis to link the progression of ES to DRGs. Our results demonstrate that genes involved in energy metabolism and mitochondrial function, including *LRPPRC*, *NDUFS1*, *NDUFA11*, *OXSM*, and *NUBPL*, are essential to the prognostic signature of ES. This finding is in contrast to reports that *SLC7A11* is the primary regulator of disulfidptosis in certain other cancers. Because of its dual function in stress adaptation and metabolic vulnerability in cancer cells, early research has suggested that the disulfidptosis pathway could be a therapeutic target. Additionally, a number of studies have documented the creation of therapeutic approaches that target this pathway specifically (27–30).

We methodically demonstrated the dysregulated expression of DRGs in ES using integrated transcriptomic analysis. Eight of the nine core DRGs that were analyzed demonstrated a noteworthy difference in expression in ES tissues; this pattern was further supported by cellular models. This persistent dysregulation implies that one of the hallmarks of ES is the disruption of metabolic pathways mediated by DRGs.

While there was an antagonistic relationship between *NDUFA11* and *NUBPL*, co-expression network analysis showed functional synergies between *NDUFA11*–*GYS1* and *NUBPL*–*OXSM*. Physical clustering of *LRPPRC*, *NCKAP1*, and *NDUFS1* within the chr2p16.1 region was found by chromosomal localization analysis, indicating possible co-regulation.

The role of DRGs in biological processes like disulfidptosis, oxidative phosphorylation, mitochondrial complex I assembly, and sulfur amino acid transport was validated by functional enrichment analysis. It is possible that the observed downregulation of sulfur amino acid transport in tumor tissues is an adaptive strategy used by ES cells to meet the demands of high-energy biosynthesis.

Three molecular clusters with different survival outcomes were identified through unsupervised clustering based on DRGs expression profiles. These clusters showed distinct immune cell infiltration patterns and notable survival differences, according to Kaplan-Meier analysis. Compared to muscle tissue, ES tumors showed reduced levels of regulatory T cells, activated CD4 T cells, Th1 cells, and neutrophils, along with elevated infiltration of memory B cells, CD56bright cells, and macrophages. These results highlight the possible contribution of immune microenvironment variability to the prognosis of ES.

We used five DRGs (*NDUFS1*, *LRPPRC*, *NDUFA11*, *OXSM*, and *NUBPL*) to create a prognostic signature. By catalyzing the first condensation step necessary for the synthesis of lipoic acid, *OXSM*, which encodes mitochondrial 3-oxoacyl-ACP synthase, acts as a crucial rate-limiting enzyme in the mitochondrial fatty acid synthesis pathway (31). High *OXSM* expression was found to be substantially linked to a better prognosis in this study for Ewing’s sarcoma, indicating a protective function that is most likely due to the preservation of mitochondrial metabolic homeostasis. Remarkably, *OXSM* has been demonstrated to function as a tumor suppressor in ovarian cancer and displays context-dependent functions (32). *NDUFS1* is a core catalytic subunit of mitochondrial complex I (33). Its overexpression indicates a poor prognosis for Ewing sarcoma, and *NDUFS1* promotes tumorigenesis by stabilizing complex I through the *PHB2* interaction in colorectal cancer (34). The main functions of the mitochondrial mRNA chaperone protein *LRPPRC* are translational coordination, polyadenylation, and transcript stabilization (35–37). According to this study, *LRPPRC* expression was considerably elevated in ES tissues, and a poor patient prognosis was strongly linked to its high expression. *NDUFA11*, a key subunit of mitochondrial complex I (38), is linked to a poor prognosis in Ewing’s sarcoma, indicating that it functions as an oncogenic driver in this situation rather than a suppressor. An assembly factor called *NUBPL* is necessary for mitochondrial complex I biogenesis (39). According to our research, a positive prognosis for ES was linked to high *NUBPL* expression, indicating a protective function. This contrasts with its role in encouraging colorectal cancer metastases (40), underscoring its context-dependent roles in cancer progression.

A risk scoring model was developed using five prognostic DRGs to efficiently classify ES patients into high-risk and low-risk categories, resulting in markedly different survival outcomes. The multivariable analysis validated the risk score as an independent prognostic indicator. Additionally, a nomogram that combined the risk score and clinical variables showed high predictive accuracy for survival at 1-year, 3-year, and 5-year, as shown by good calibration curves.

The high-risk group displayed a unique immune profile, marked by increased infiltration of activated CD4 T cells, CD56bright cells, CD56dim cells, NK cells, NKT cells, Th2 cells, and immature dendritic cells. At the same time, this group exhibited enhanced immunosuppressive mechanisms, such as T-cell co-inhibition and antigen-presenting cell (APC) co-inhibition. This discovery aligns with recent single-cell research that linked a subset of lipid-metabolizing macrophages to bone tumor tissue remodeling and immune suppression (41).

We also observed a strong link between the chemotherapeutic reaction and the DRGs expression patterns. For instance, hydroxyurea resistance was linked to high expression of *NDUFS1* and *NDUFA11*, while dasatinib sensitivity was negatively correlated with *OXSM* expression. The low-risk group had heightened sensitivity to microtubule inhibitors, according to risk-based stratification.

Molecular docking simulations further supported these associations, demonstrating stable binding between all five DRGs proteins and multiple chemotherapeutic compounds, suggesting potential direct interactions that may modulate drug sensitivity.

Single-cell RNA sequencing analysis provided critical insights into the cellular context of these DRGs. The five prognostic genes were predominantly enriched in malignant ES cells, confirming their tumor-intrinsic roles. Notably, *NDUFA11*-high malignant cells exhibited distinct transcriptional programs enriched in mitochondrial function, ribosome biogenesis, and oxidative phosphorylation, indicating enhanced metabolic activity. Furthermore, intercellular communication analysis revealed that *NDUFA11*-high ES cells engaged in stronger crosstalk with various tumor microenvironment components, including T cells, myeloid cells, and CAFs. These findings suggest that *NDUFA11* not only drives malignant cell metabolism but also shapes the immunosuppressive tumor microenvironment, contributing to poor prognosis.

In accordance with transcriptomic data from tumor tissues, experimental validation in the RD-ES cell line verified the dysregulation of all five prognostic DRGs at the RNA and protein levels. The discrepancy between *LRPPRC* protein and mRNA levels likely reflects post-transcriptional regulation. mRNA levels do not always correlate with protein abundance due to differences in translation efficiency, protein stability, or degradation rates. LRPPRC is known to be involved in complex regulatory networks, making such discordance biologically plausible.Additionally, technical factors such as antibody specificity, detection sensitivity, or sample timing may contribute to the observed differences.

To further investigate the functional significance of *NDUFA11* in ES, we performed siRNA-mediated knockdown in RD-ES cells. Colony formation assays revealed that silencing *NDUFA11* significantly suppressed the proliferative capacity of ES cells, suggesting that *NDUFA11* may act as an oncogenic driver promoting tumor growth. Moreover, wound healing and Transwell migration assays demonstrated that *NDUFA11* knockdown markedly reduced the migratory potential of RD-ES cells, indicating its involvement in ES metastasis. These functional results are consistent with its identification as a high-risk gene in our prognostic model: elevated *NDUFA11* expression predicts poor clinical outcomes, and its silencing attenuates malignant phenotypes, thereby reinforcing the biological plausibility of the signature.

The main limitations of this study are its reliance on bioinformatic analysis and the limited sample size, rather than providing direct evidence of disulfidptosis in ES cells or the functional roles of DRGs in controlling this process. To confirm disulfidptosis in ES and clarify the regulatory mechanisms of DRGs *in vitro* and *in vivo*, future research should combine morphological and biochemical assays with induction models.

## 5 Conclusion

We identified five DRGs (*NDUFS1*, *LRPPRC*, *NDUFA11*, *OXSM*, *NUBPL*) prognostic for ES. A risk model based on these genes correlated with patient survival and immune features. Single−cell analysis showed enrichment in malignant cells, with *NDUFA11* driving metabolic heterogeneity and intercellular communication. Functional assays confirmed *NDUFA11* knockdown suppressed ES cell proliferation, migration, and invasion, supporting its oncogenic role and therapeutic potential. These findings provide a basis for targeted therapies, though direct validation of disulfidptosis mechanisms is needed.

## Data Availability

The original contributions presented in the study are included in the article/**Supplementary Material**. Further inquiries can be directed to the corresponding author.
