# Supplementary figures and images for: Disulfidptosis-related genes define a prognostic signature and novel therapeutic targets in Ewing’s sarcoma through transcriptomic analysis and experimental validation
Source: Front Immunol. 2026 Apr 15;17:1722729. doi: 10.3389/fimmu.2026.1722729 (PMC13125098; doi:10.3389/fimmu.2026.1722729)

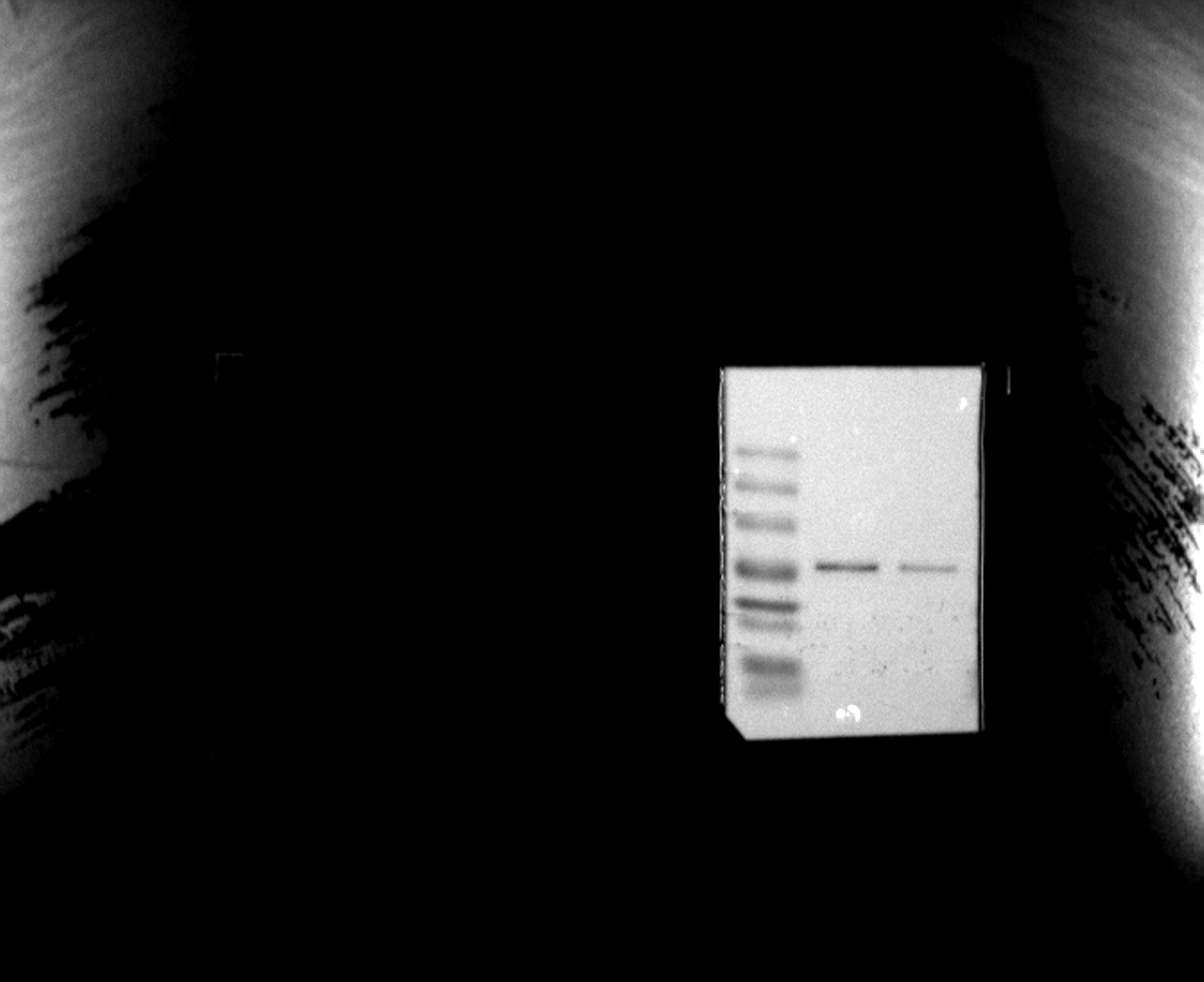

Supplement: Supplementary file 1 [file DataSheet1.zip › original.gel1/NDUFS1 1-2.Tif]

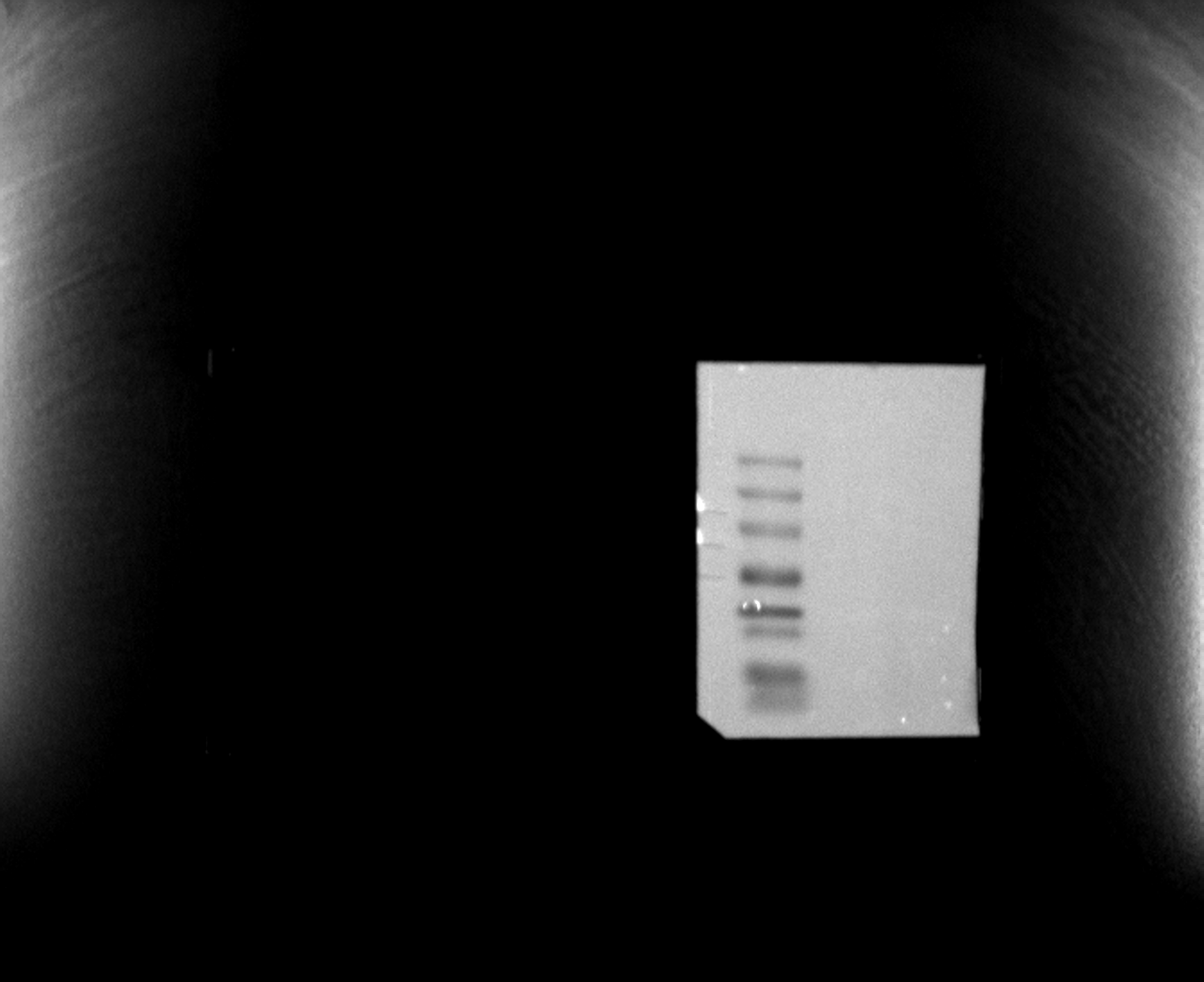

Supplement: Supplementary file 1 [file DataSheet1.zip › original.gel1/LRPRC 1-1.Tif]

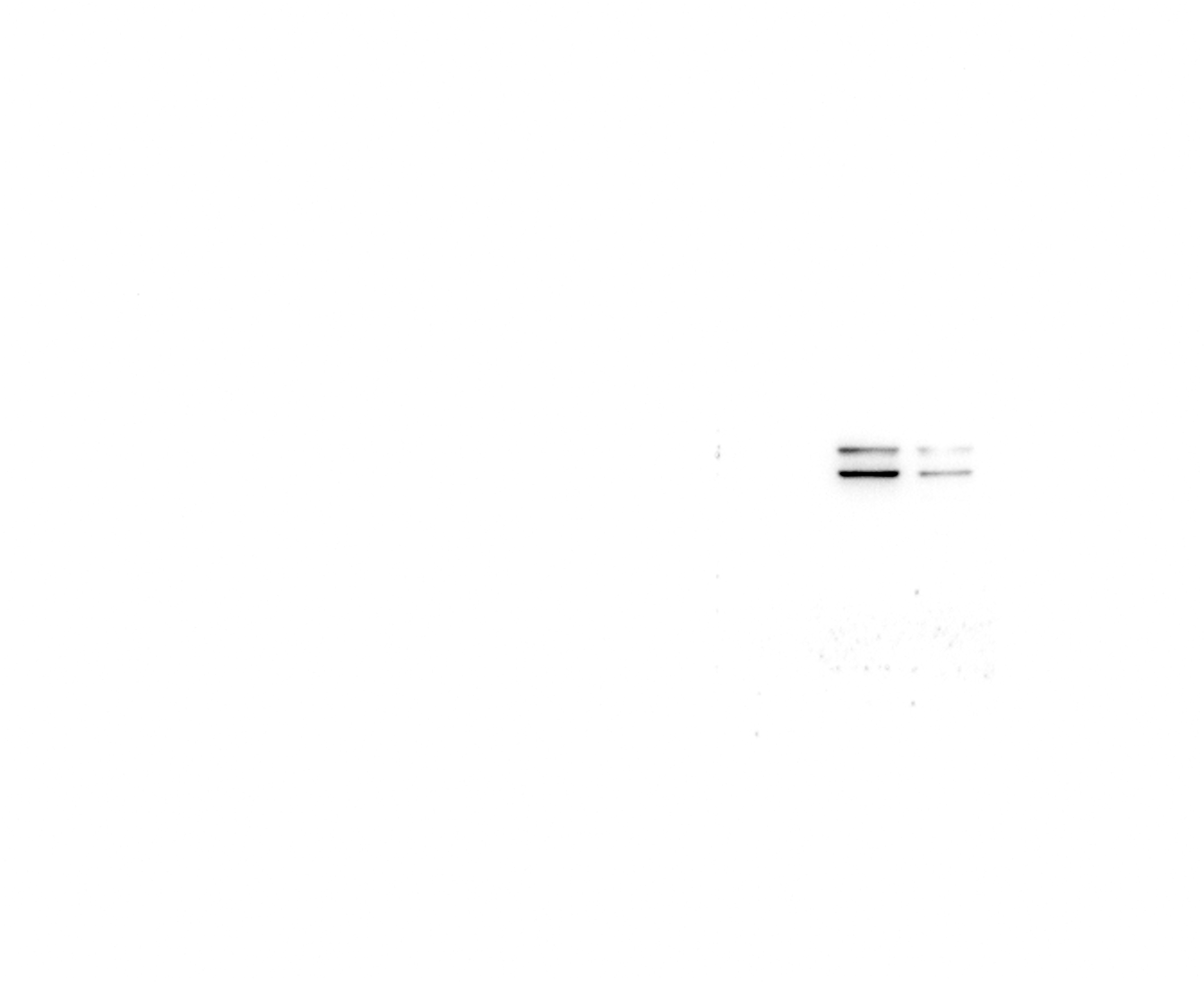

Supplement: Supplementary file 1 [file DataSheet1.zip › original.gel1/OXSM 1.Tif]

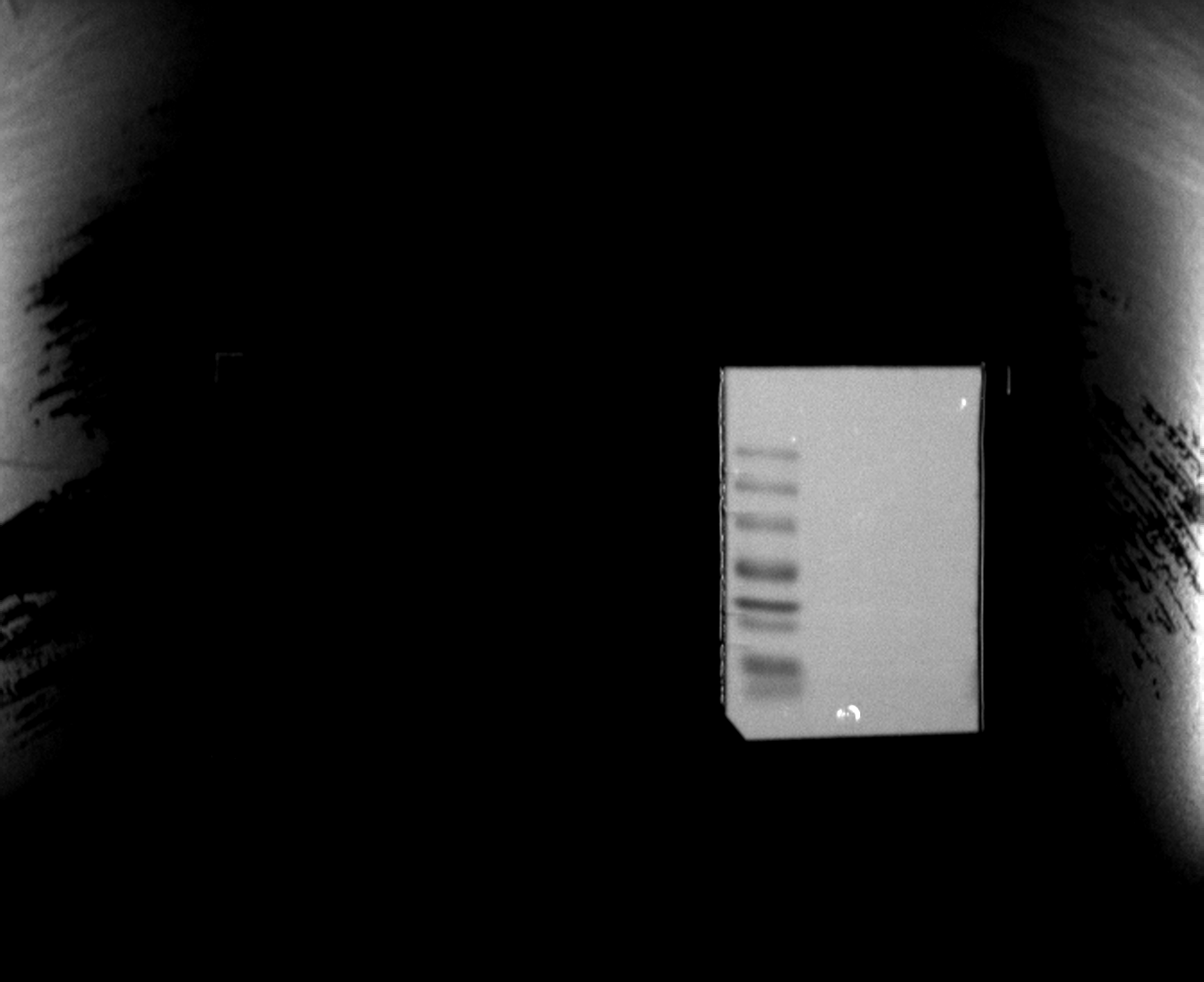

Supplement: Supplementary file 1 [file DataSheet1.zip › original.gel1/NDUFS1 1-1.Tif]

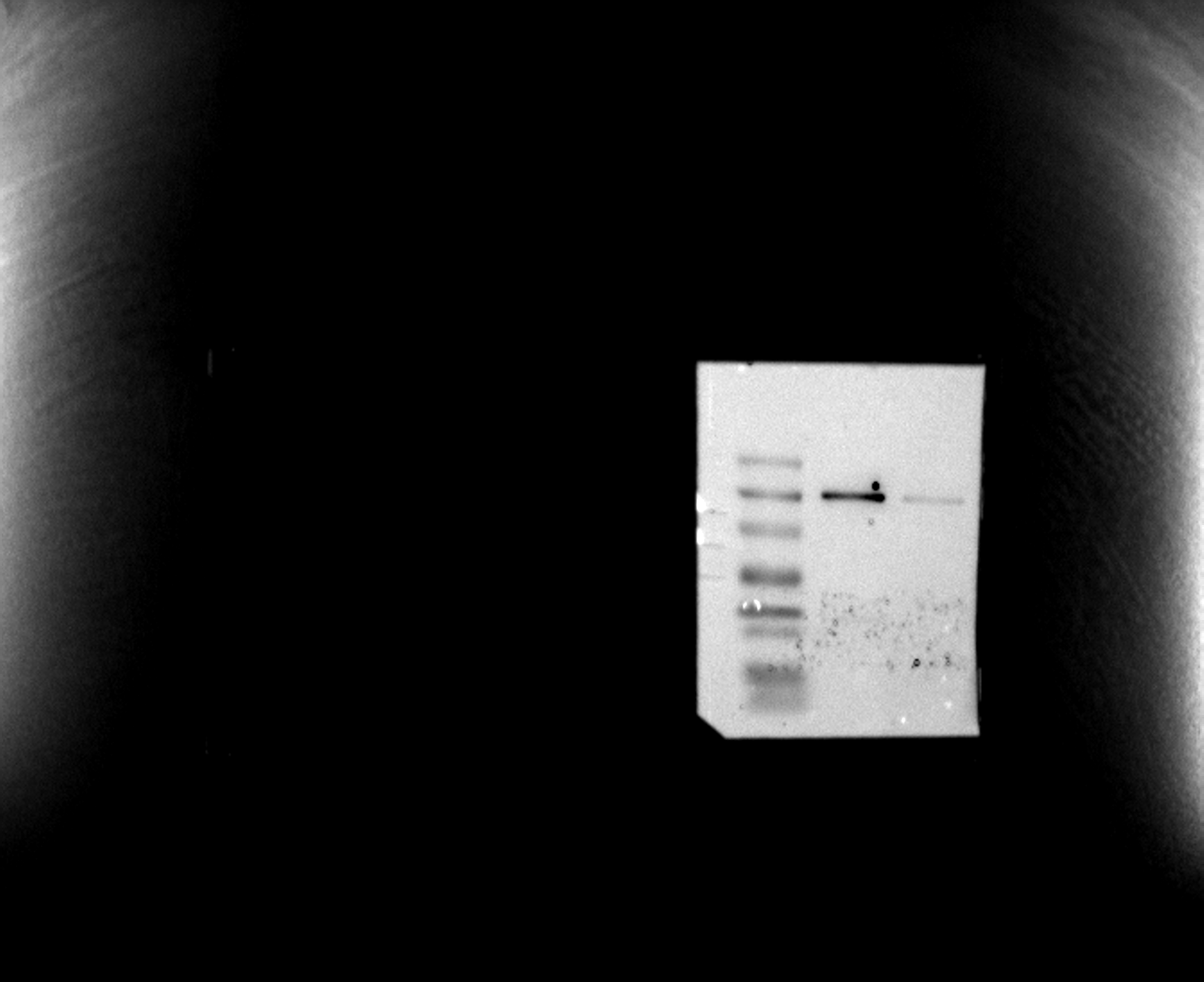

Supplement: Supplementary file 1 [file DataSheet1.zip › original.gel1/LRPRC 1-2.Tif]

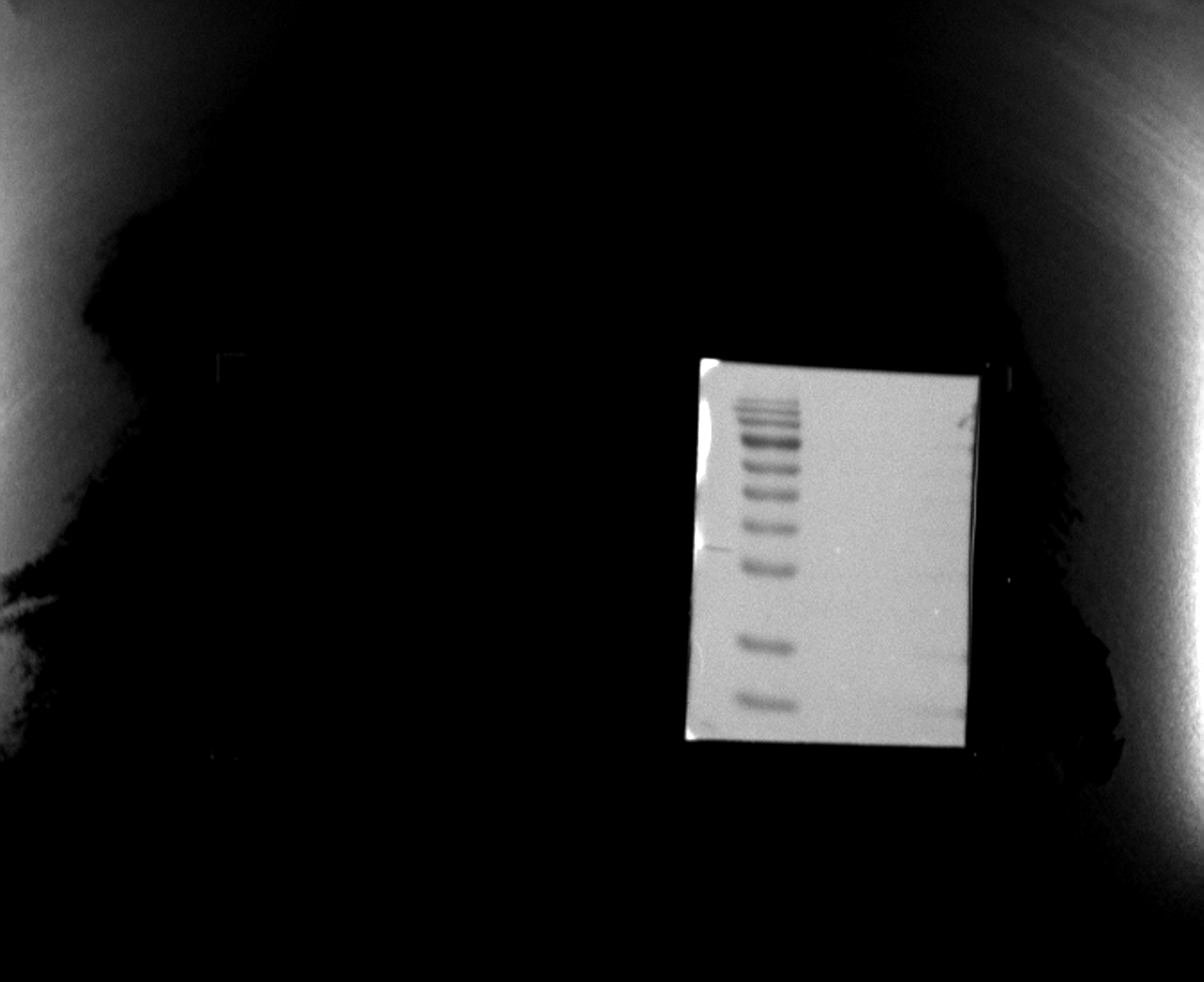

Supplement: Supplementary file 1 [file DataSheet1.zip › original.gel1/GAPDH 1-1.Tif]

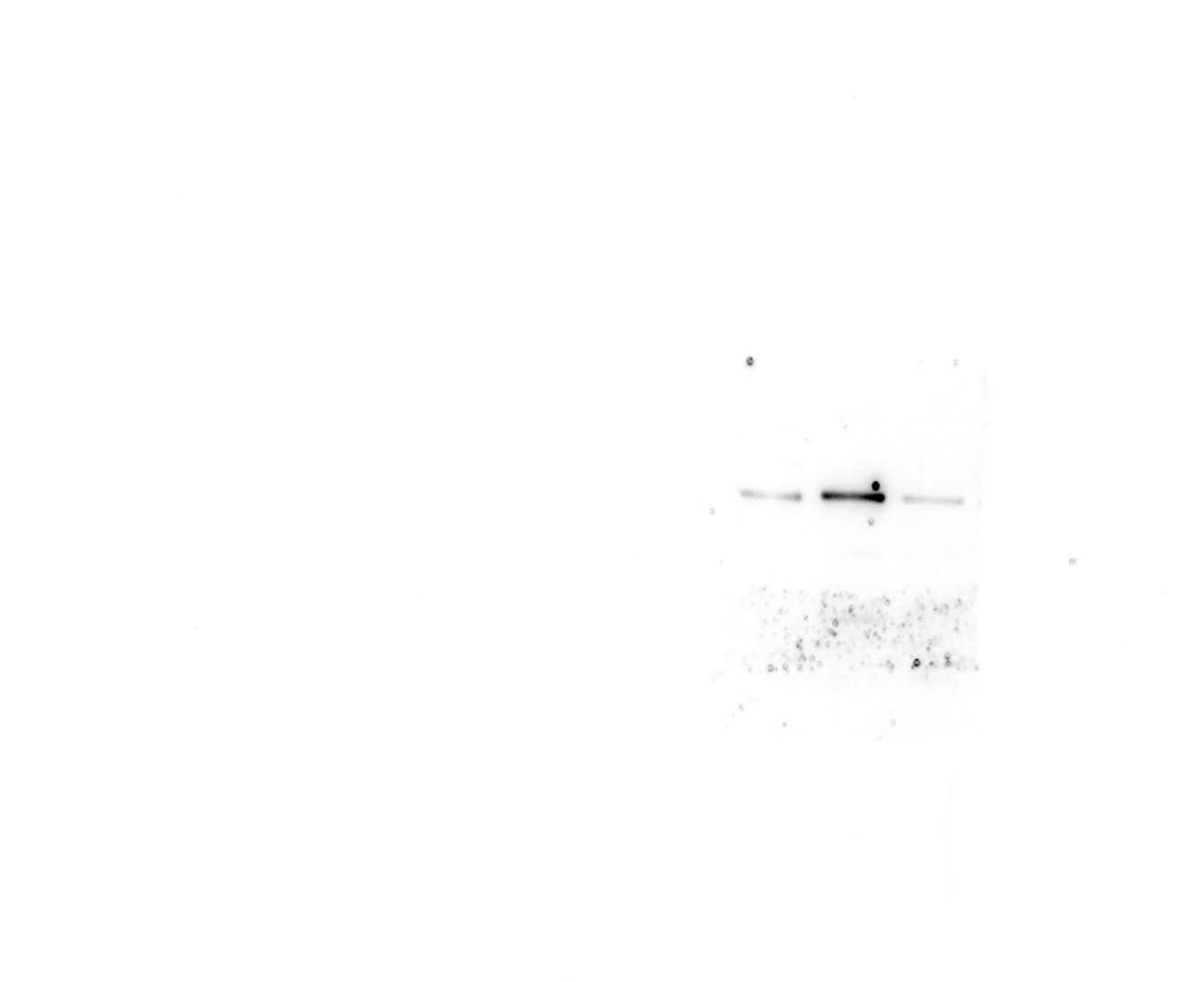

Supplement: Supplementary file 1 [file DataSheet1.zip › original.gel1/LRPRC 1.Tif]

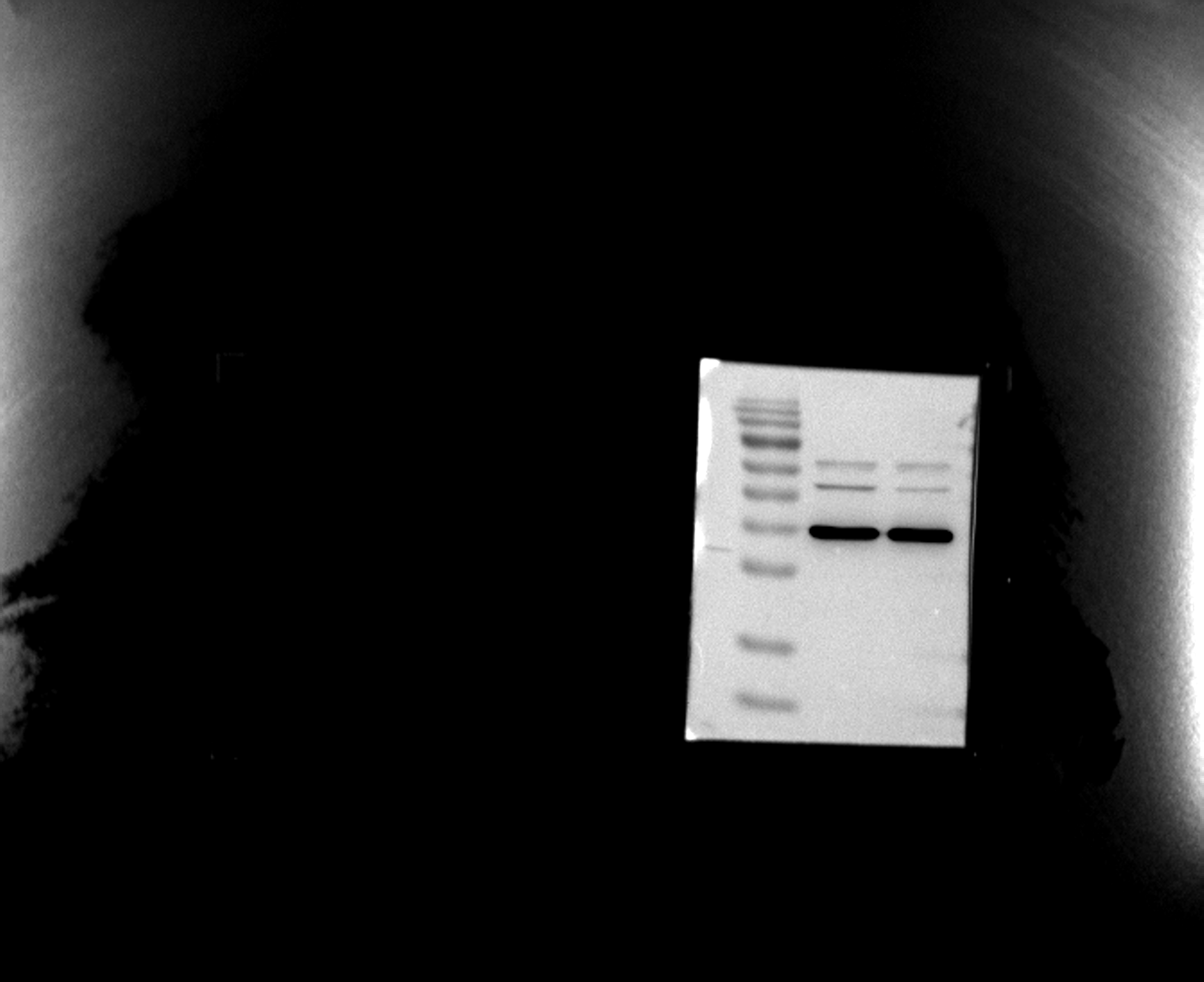

Supplement: Supplementary file 1 [file DataSheet1.zip › original.gel1/GAPDH 1-2.Tif]

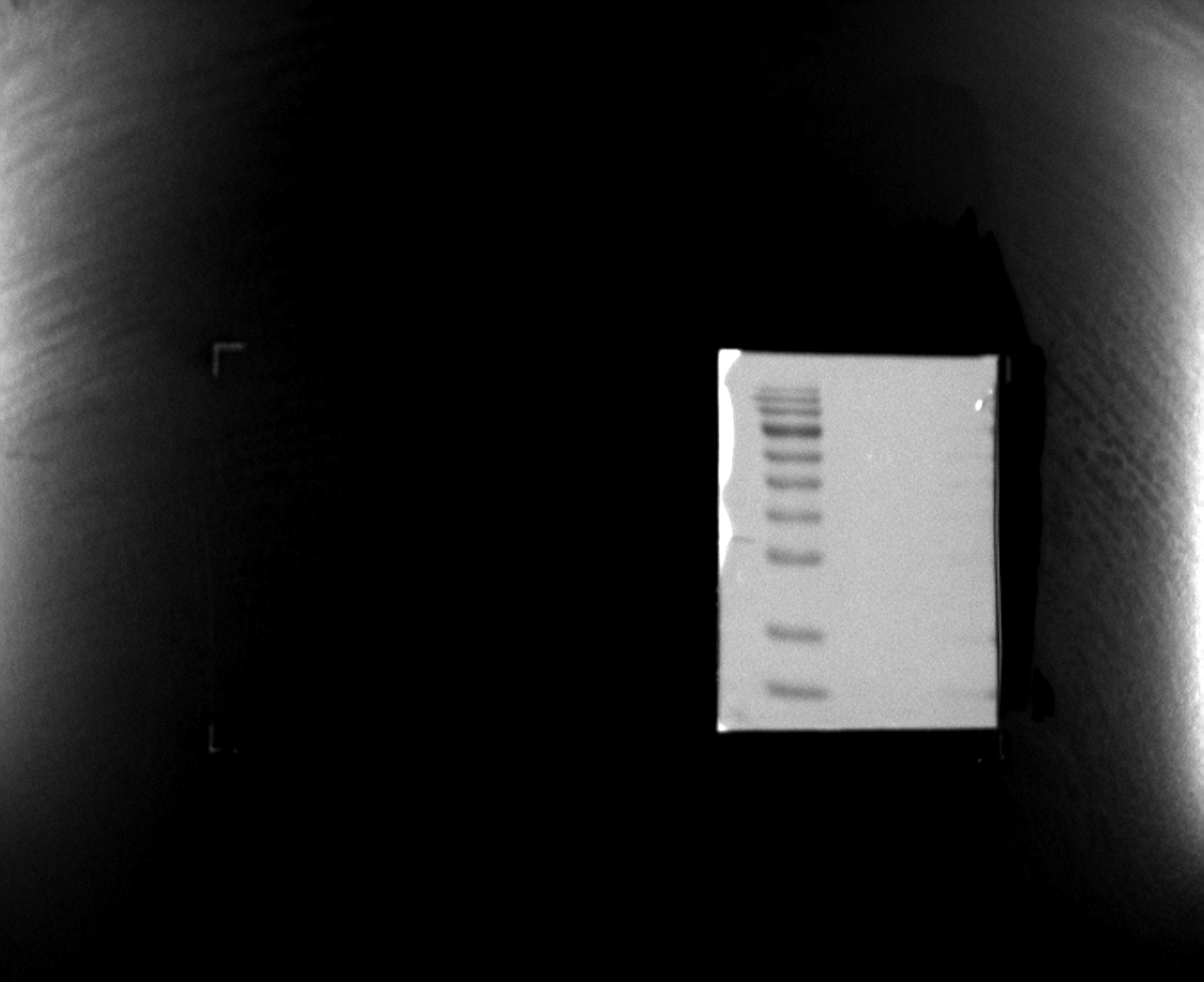

Supplement: Supplementary file 1 [file DataSheet1.zip › original.gel1/OXSM 1-1.Tif]

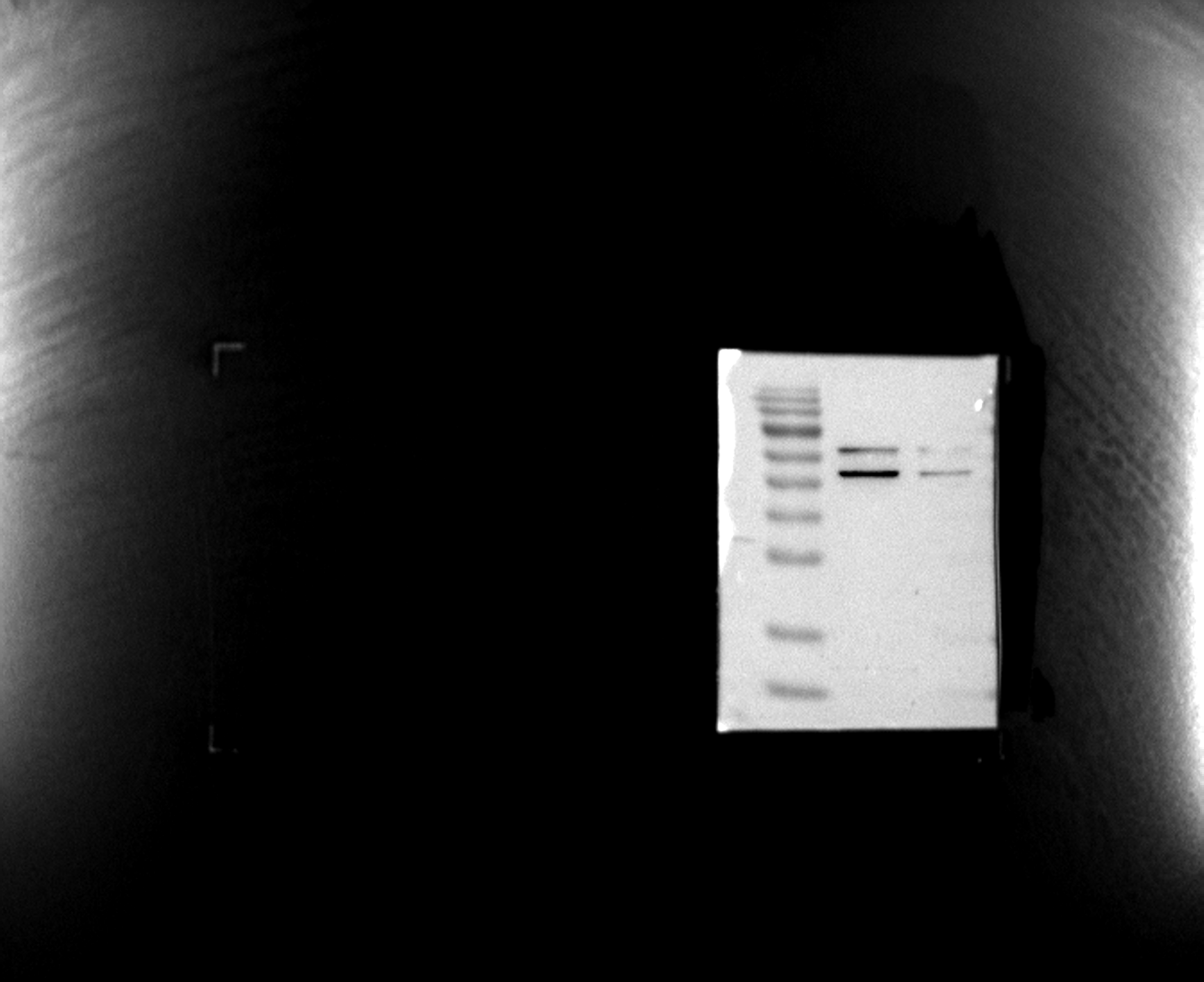

Supplement: Supplementary file 1 [file DataSheet1.zip › original.gel1/OXSM 1-2.Tif]

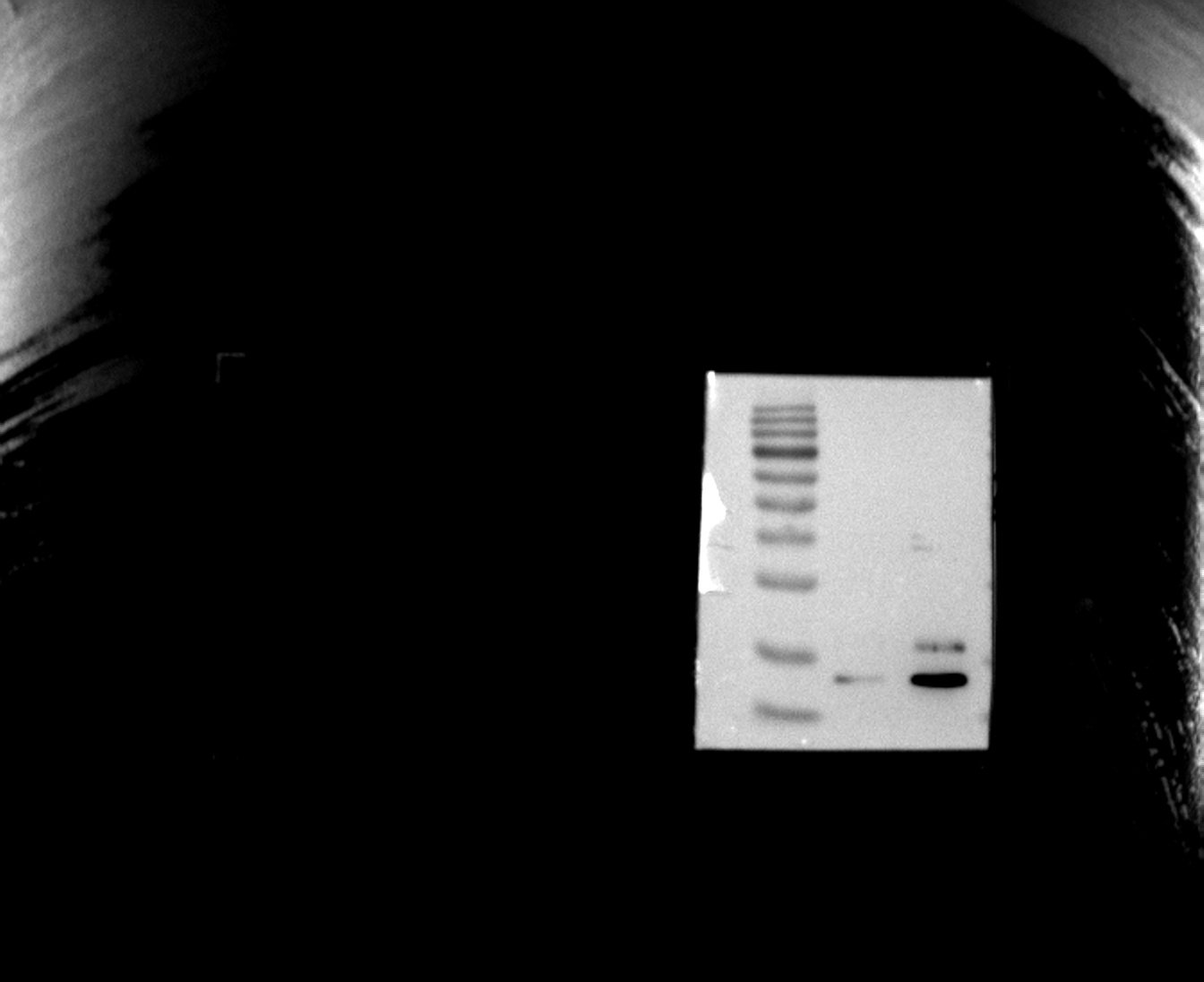

Supplement: Supplementary file 1 [file DataSheet1.zip › original.gel1/NDUFA11 1-2.Tif]

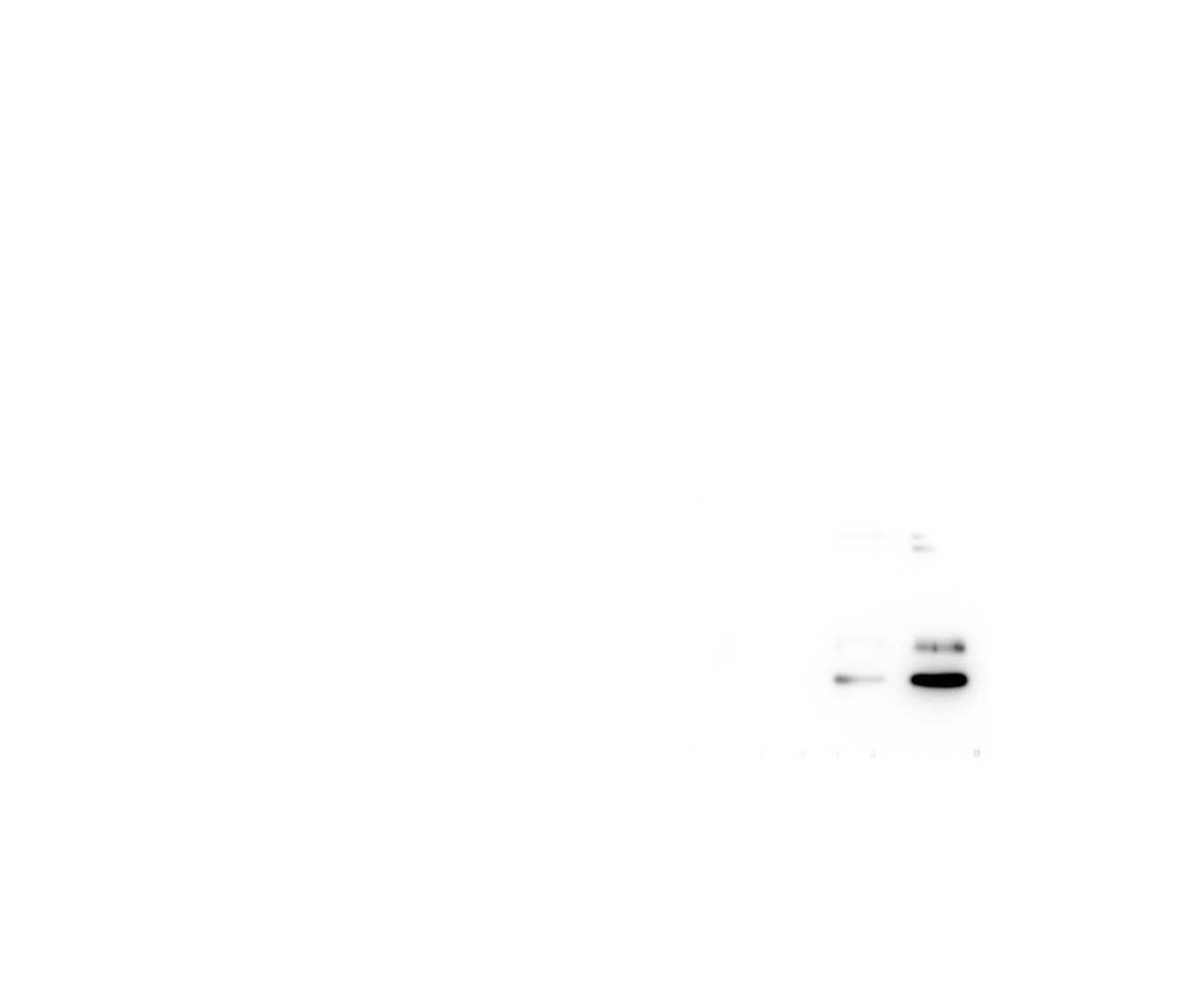

Supplement: Supplementary file 1 [file DataSheet1.zip › original.gel1/NDUFA11 1.Tif]

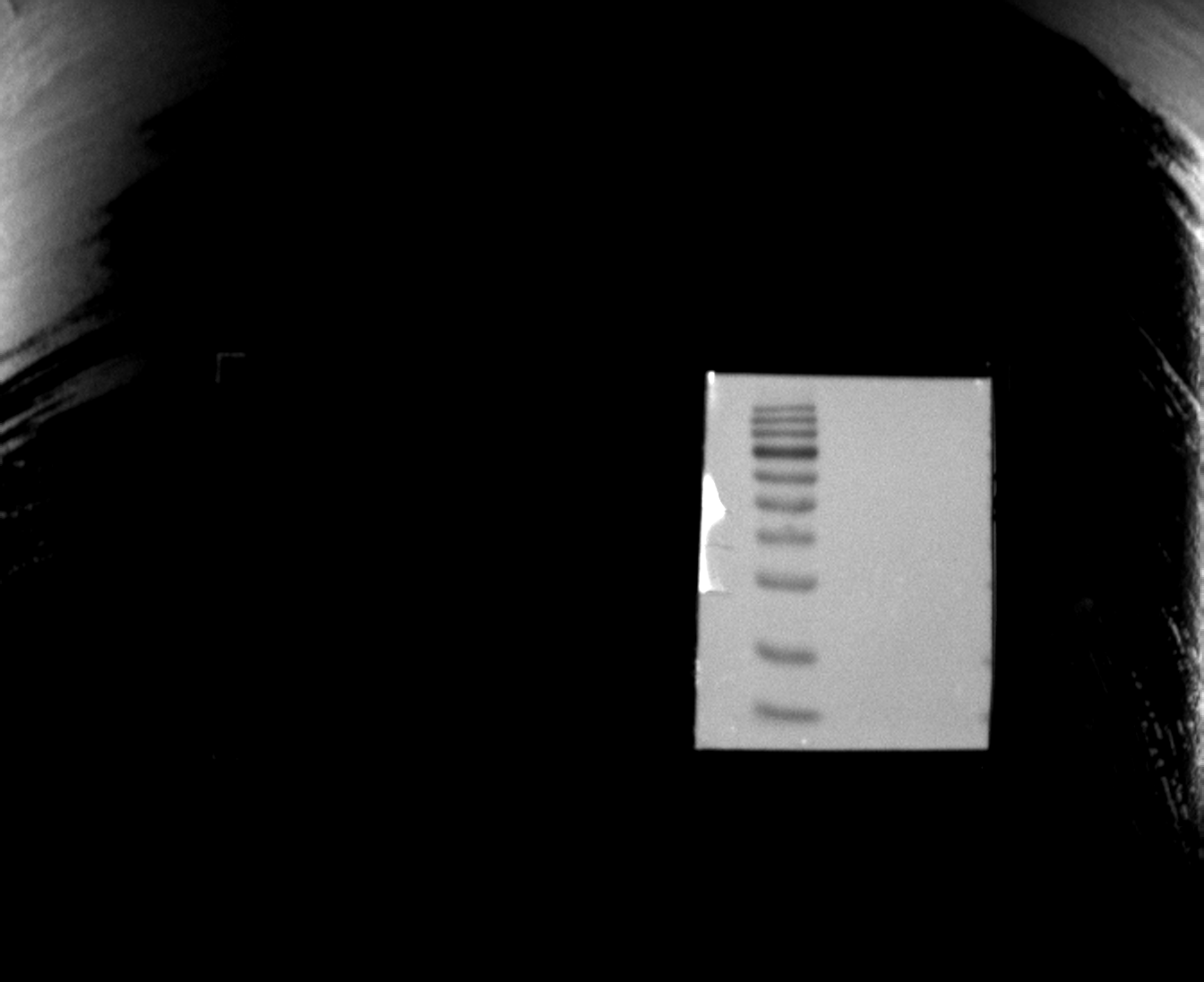

Supplement: Supplementary file 1 [file DataSheet1.zip › original.gel1/NDUFA11 1-1.Tif]

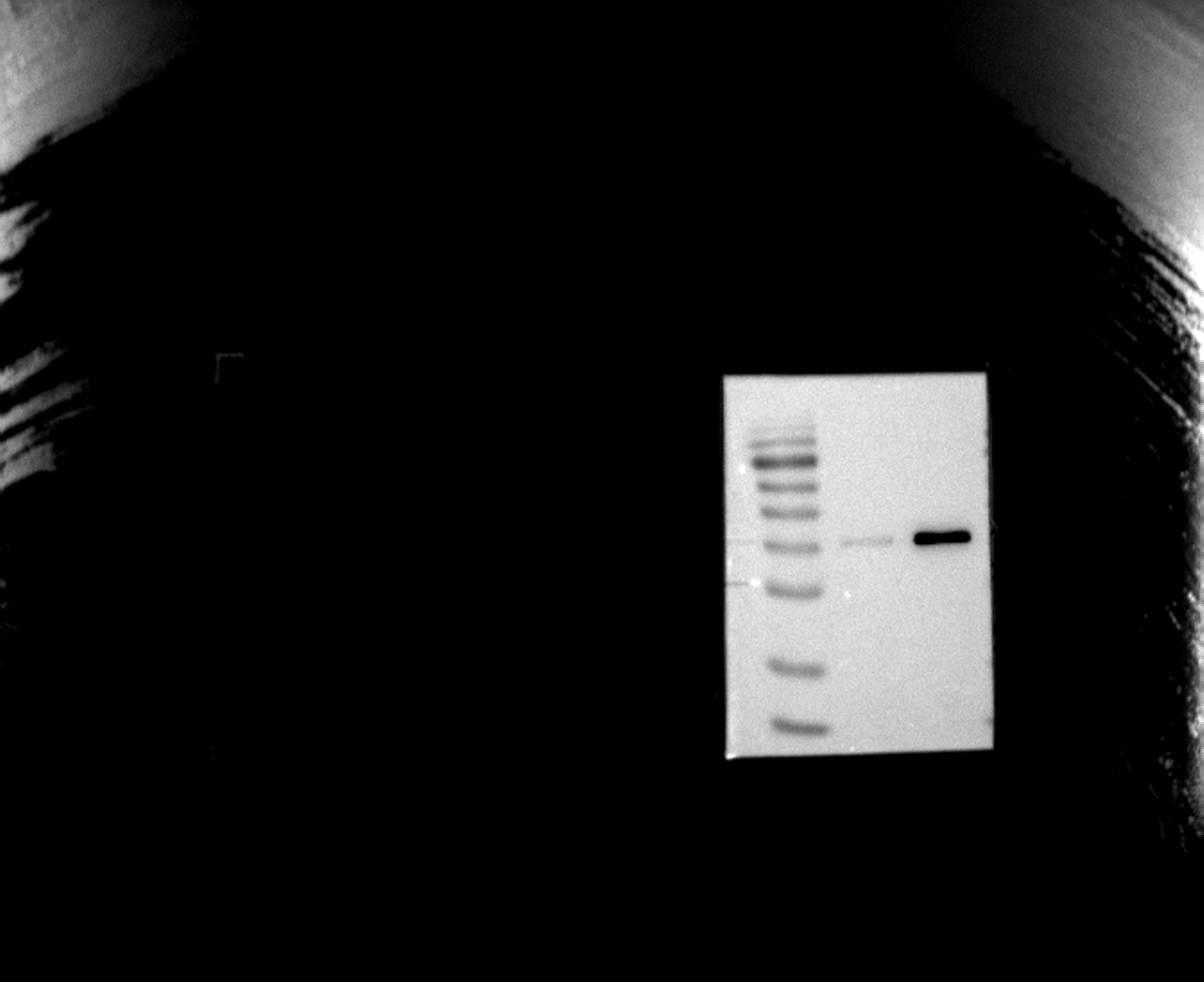

Supplement: Supplementary file 1 [file DataSheet1.zip › original.gel1/NUBPL 1-2.Tif]

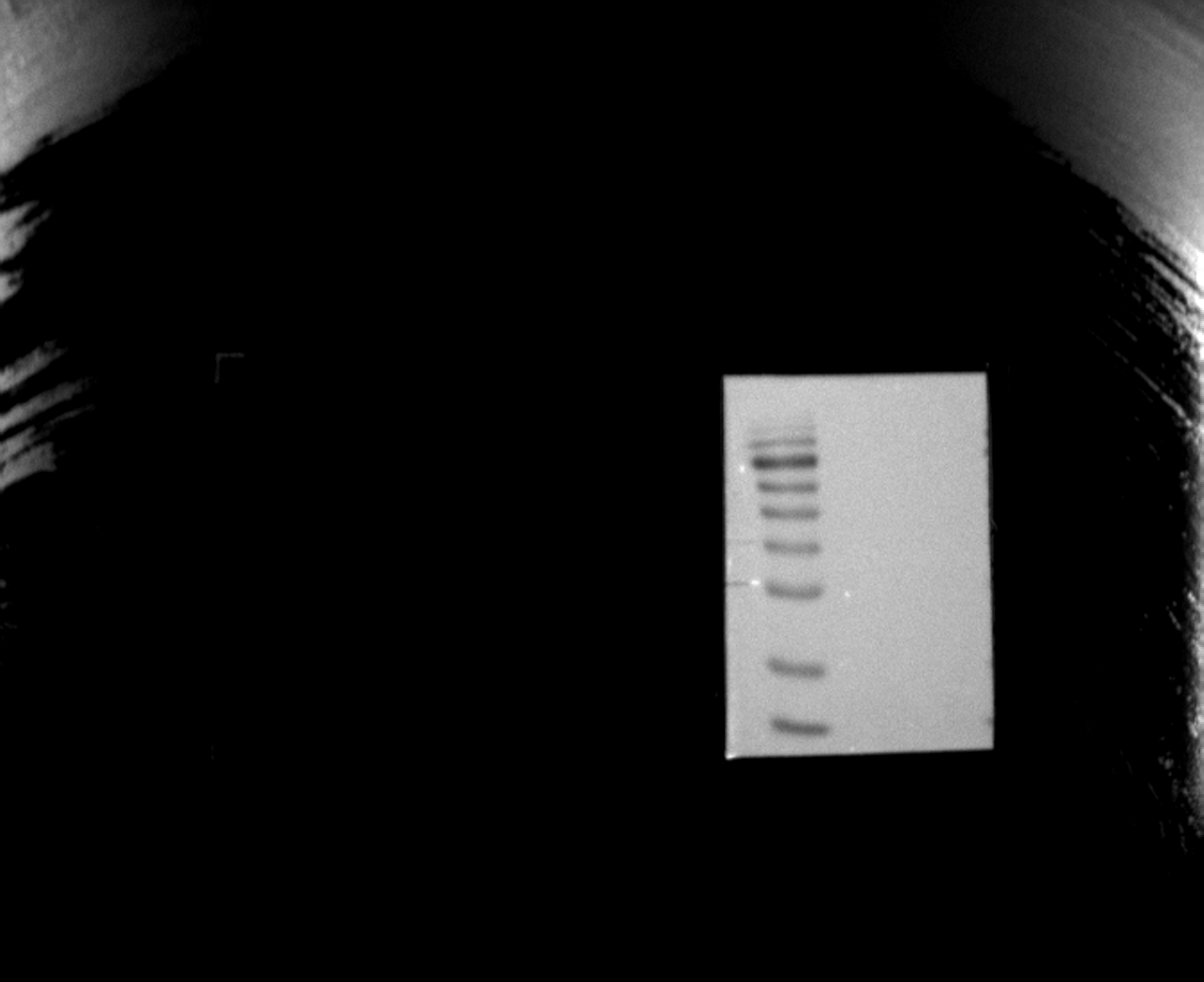

Supplement: Supplementary file 1 [file DataSheet1.zip › original.gel1/NUBPL 1-1.Tif]

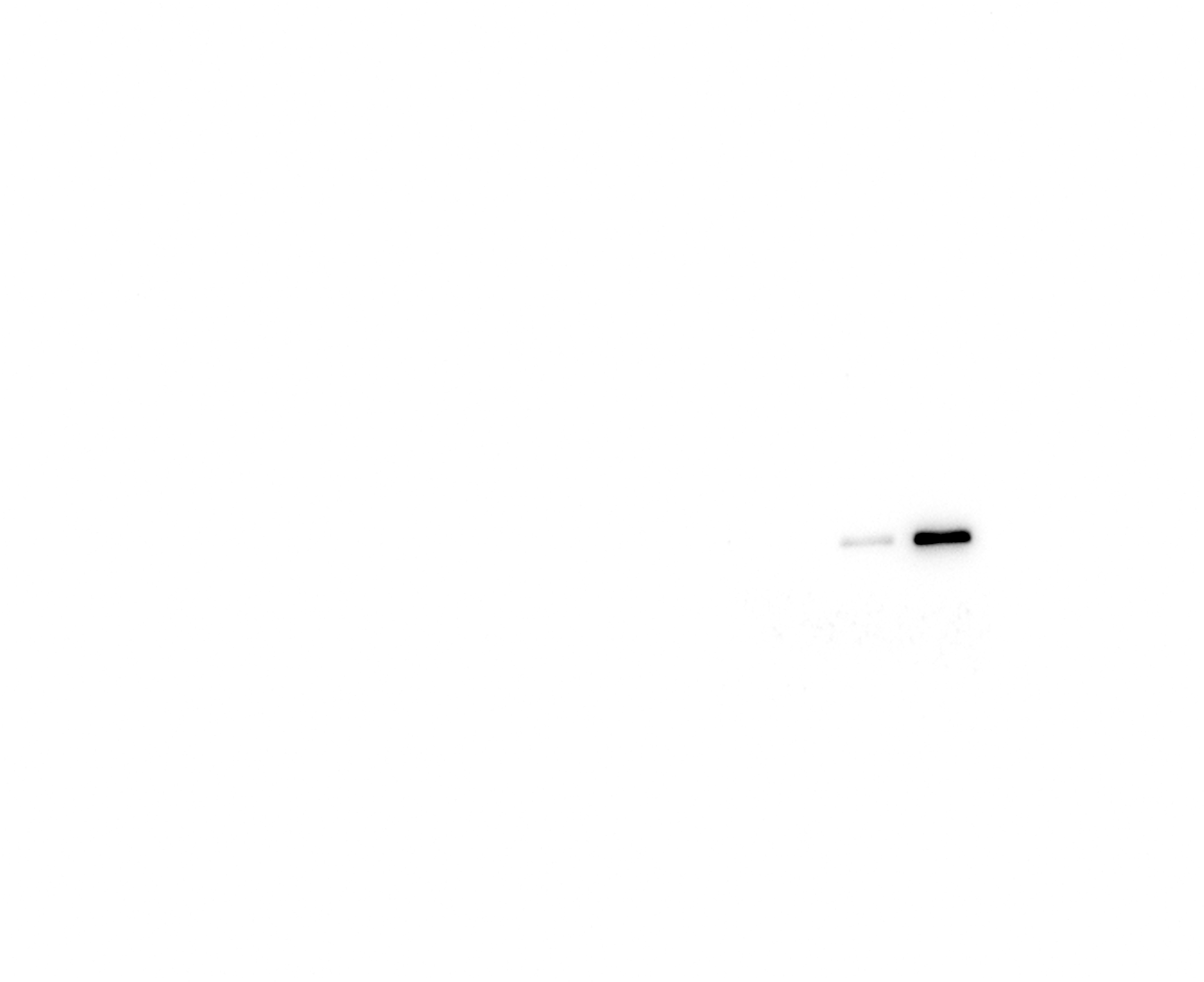

Supplement: Supplementary file 1 [file DataSheet1.zip › original.gel1/NUBPL 1.Tif]

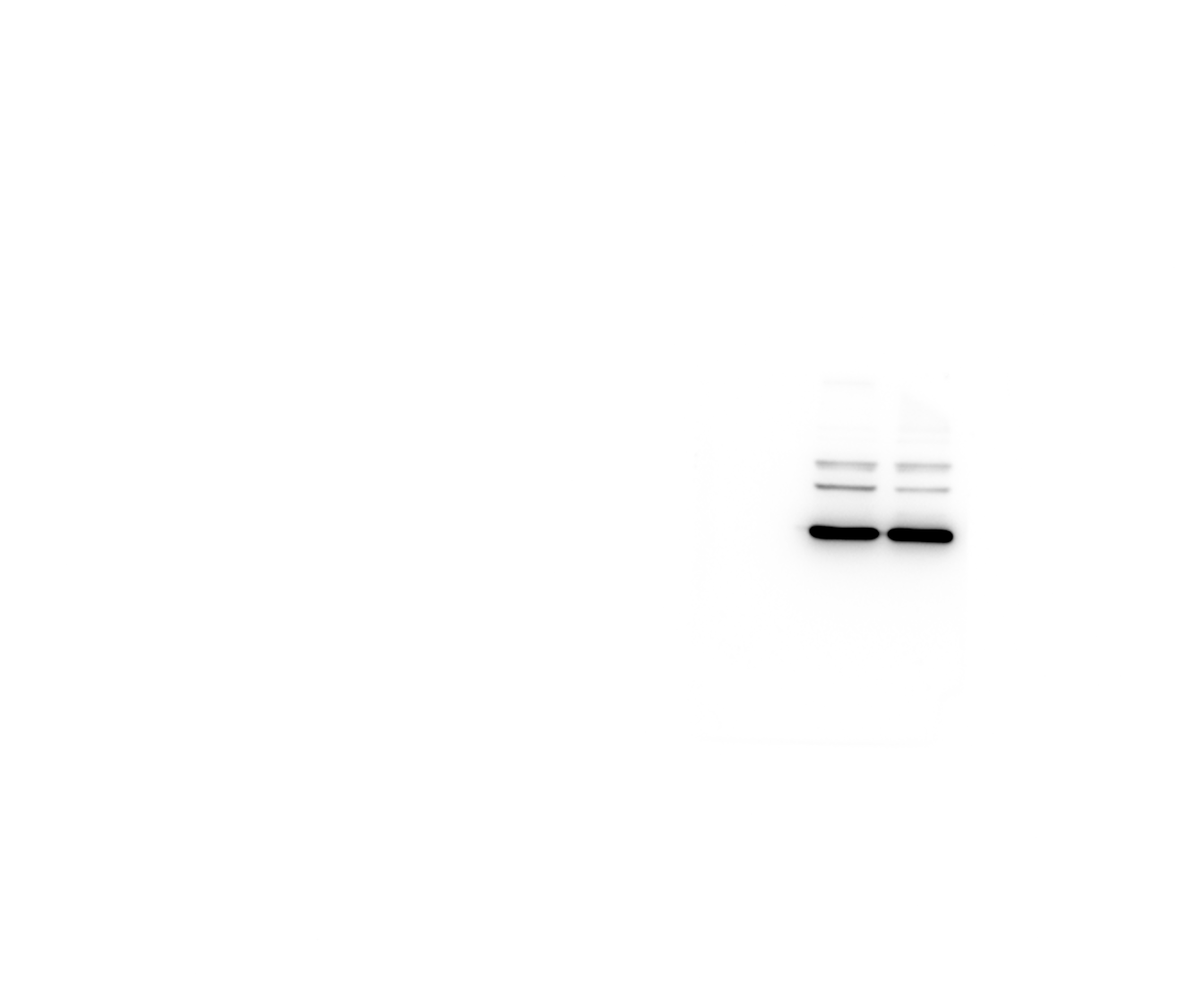

Supplement: Supplementary file 1 [file DataSheet1.zip › original.gel1/GAPDH 1.Tif]

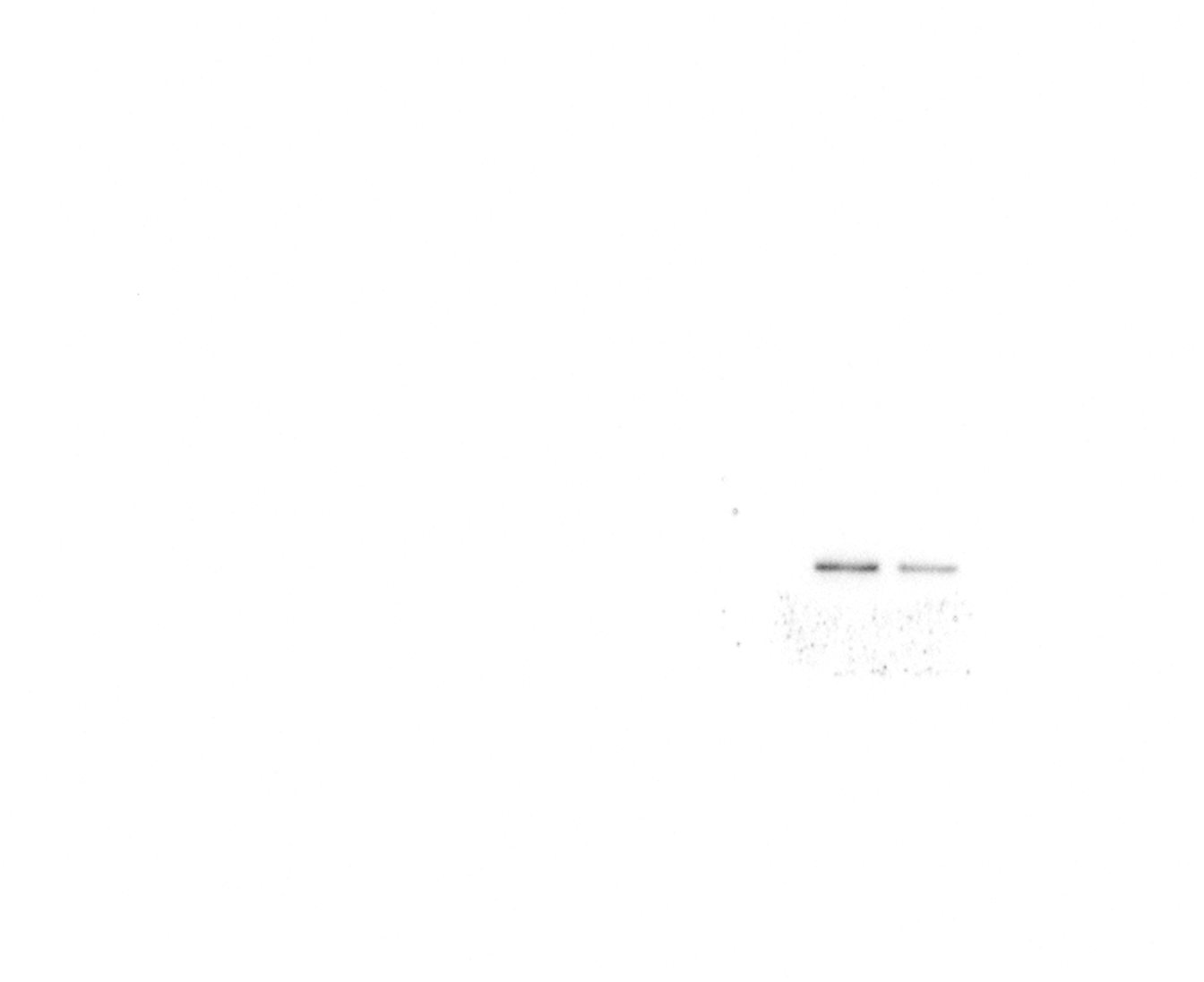

Supplement: Supplementary file 1 [file DataSheet1.zip › original.gel1/NDUFS1 1.Tif]

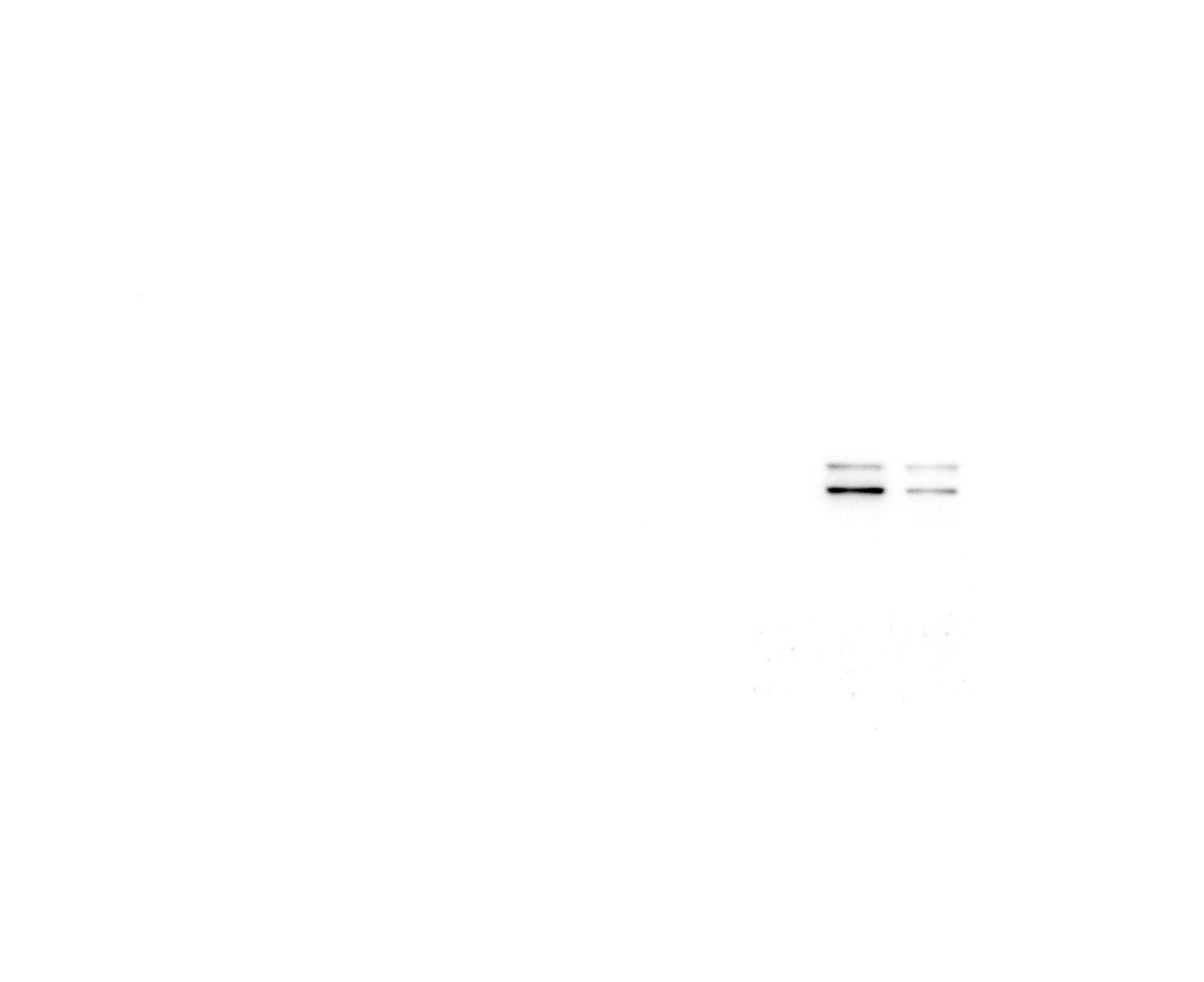

Supplement: Supplementary file 2 [file DataSheet2.zip › original.gel2/OXSM 2.Tif]

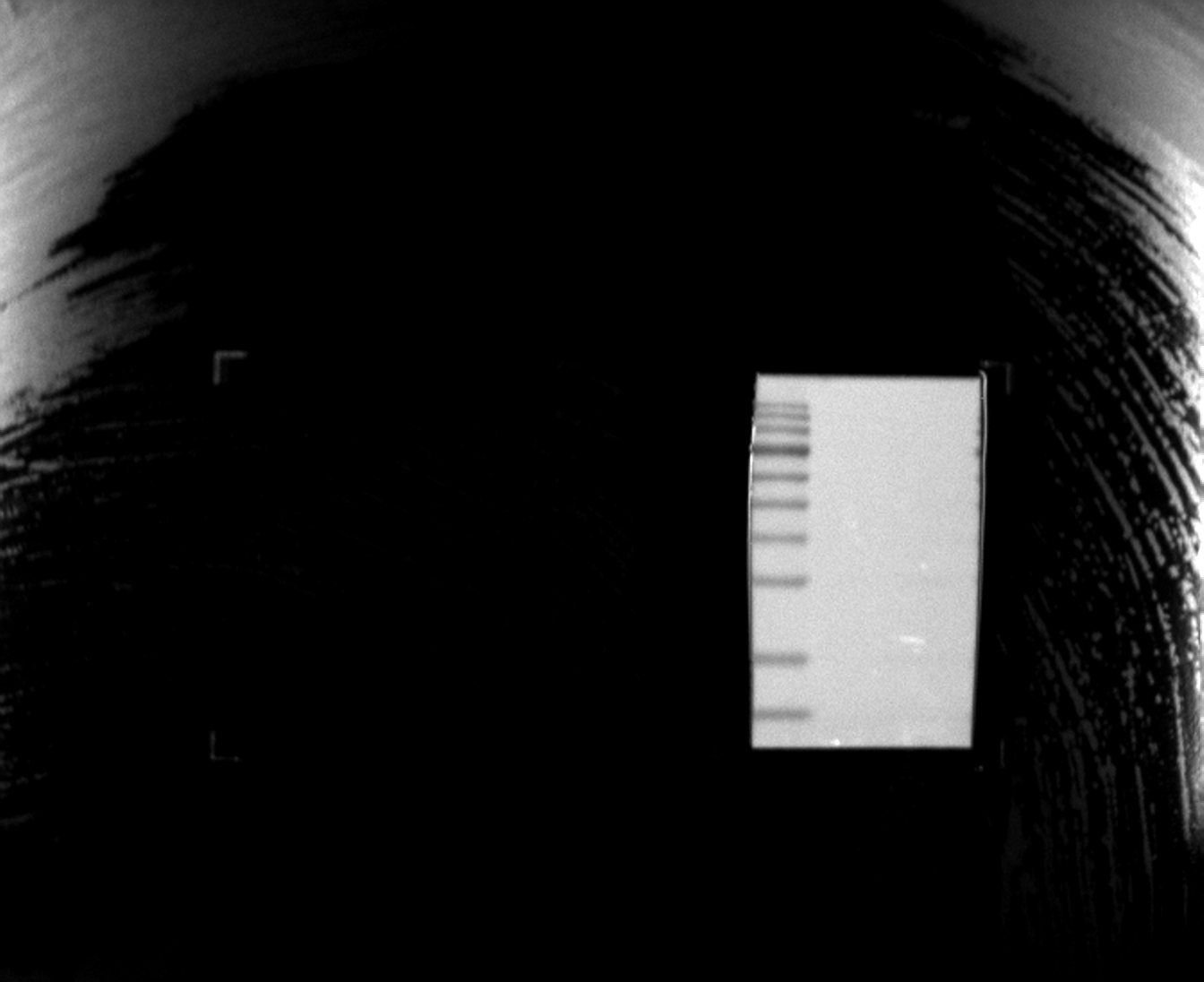

Supplement: Supplementary file 2 [file DataSheet2.zip › original.gel2/OXSM 2-1.Tif]

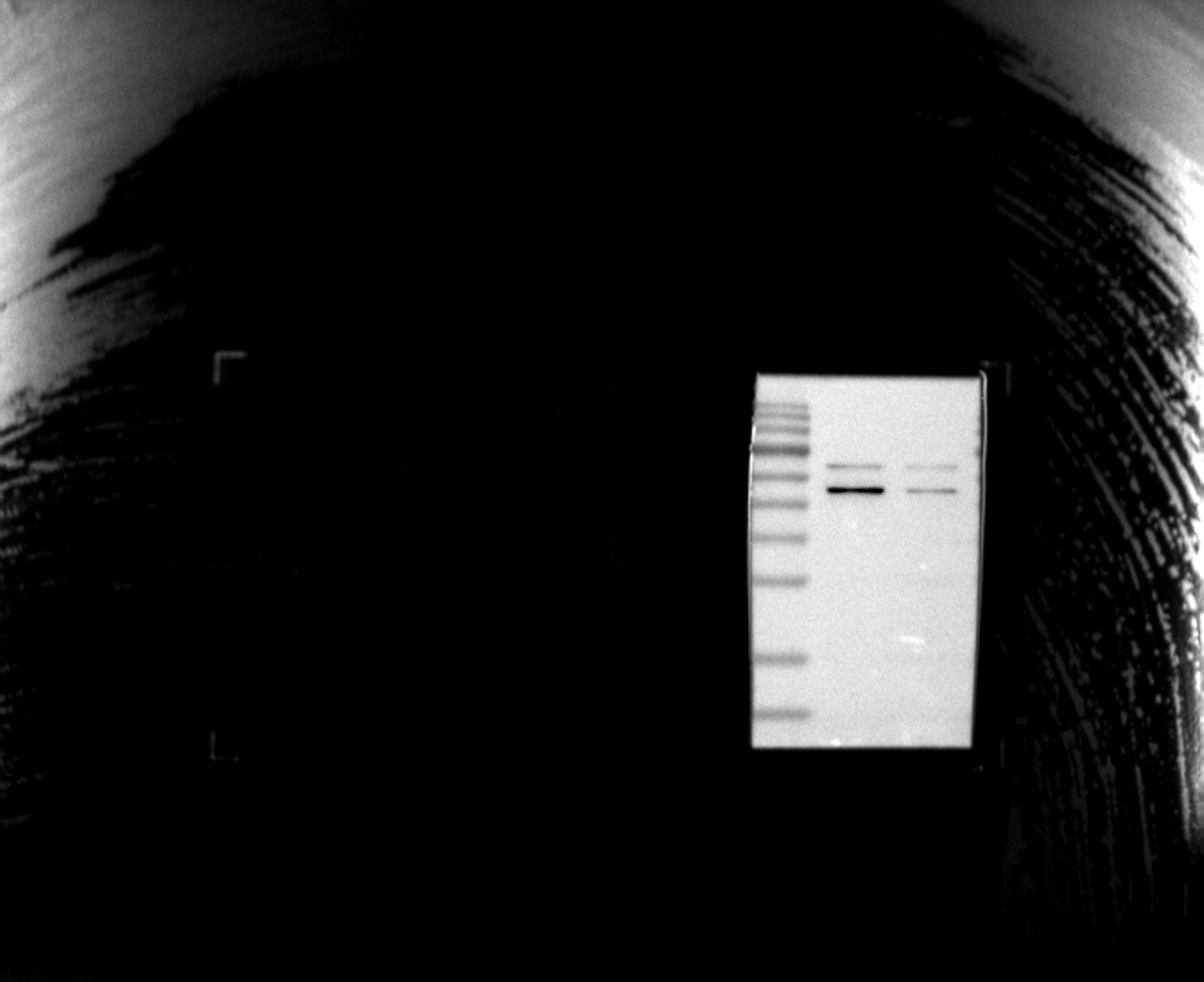

Supplement: Supplementary file 2 [file DataSheet2.zip › original.gel2/OXSM 2-2.Tif]

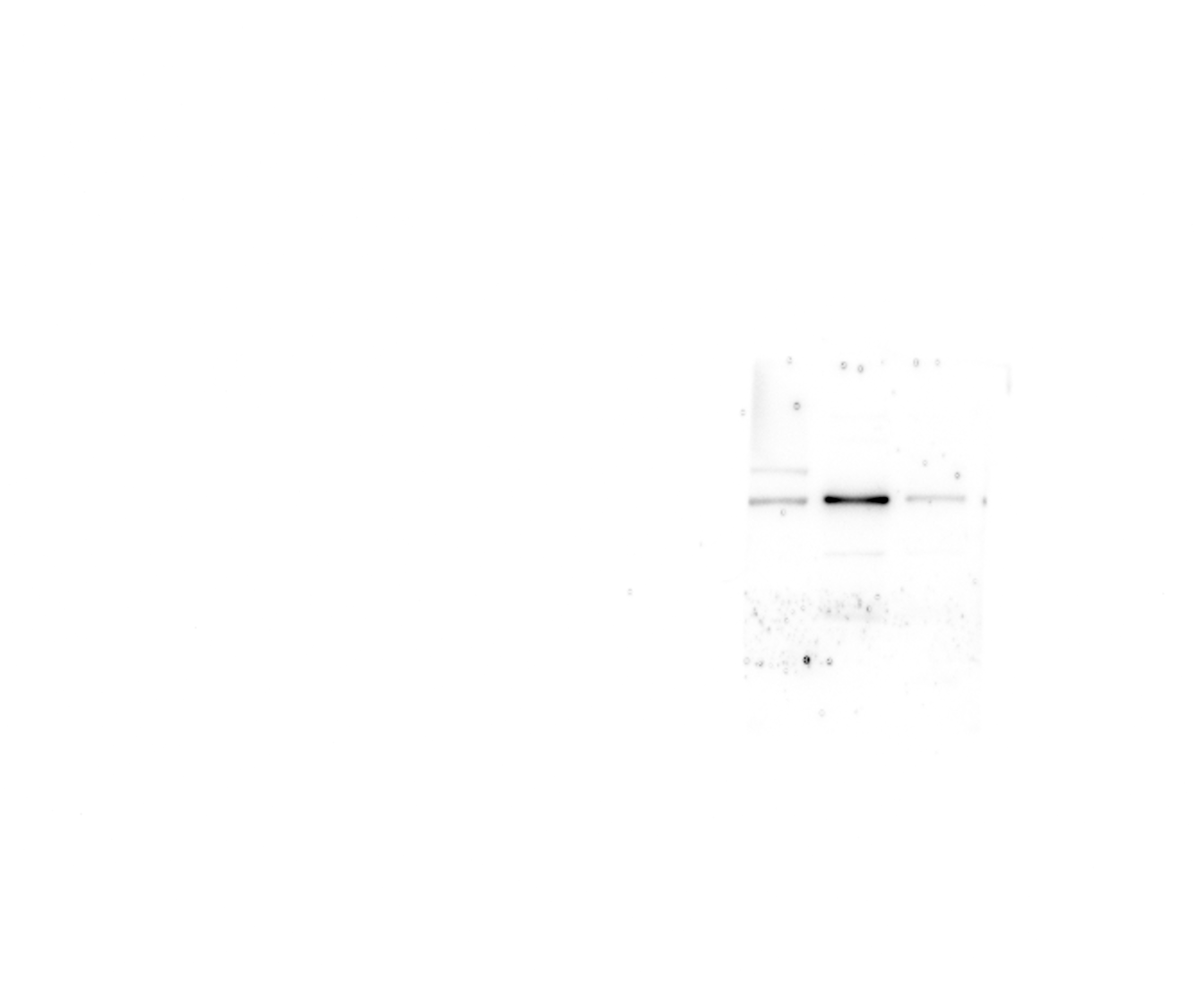

Supplement: Supplementary file 2 [file DataSheet2.zip › original.gel2/LRPRC 2.Tif]

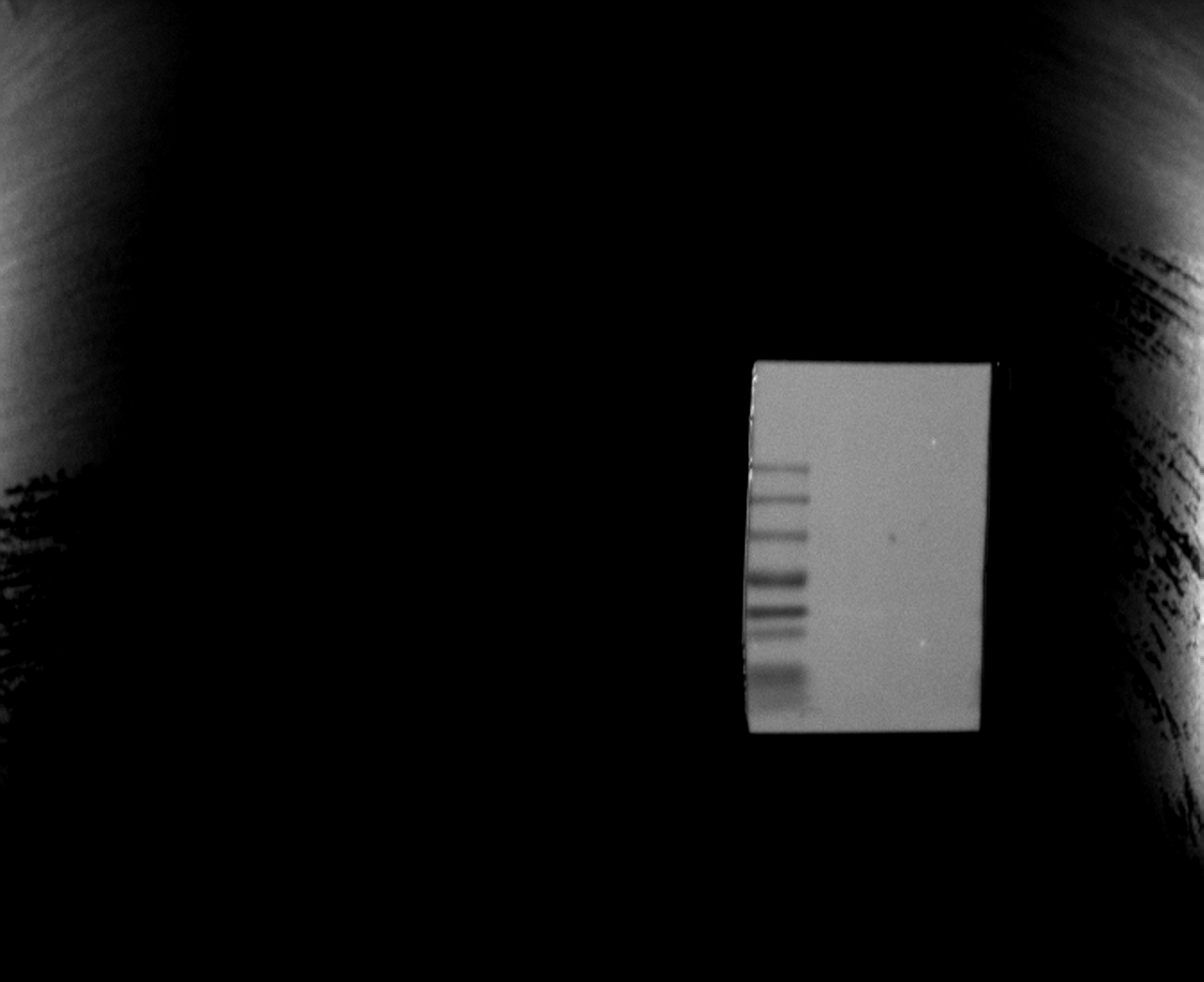

Supplement: Supplementary file 2 [file DataSheet2.zip › original.gel2/LRPRC 2-1.Tif]

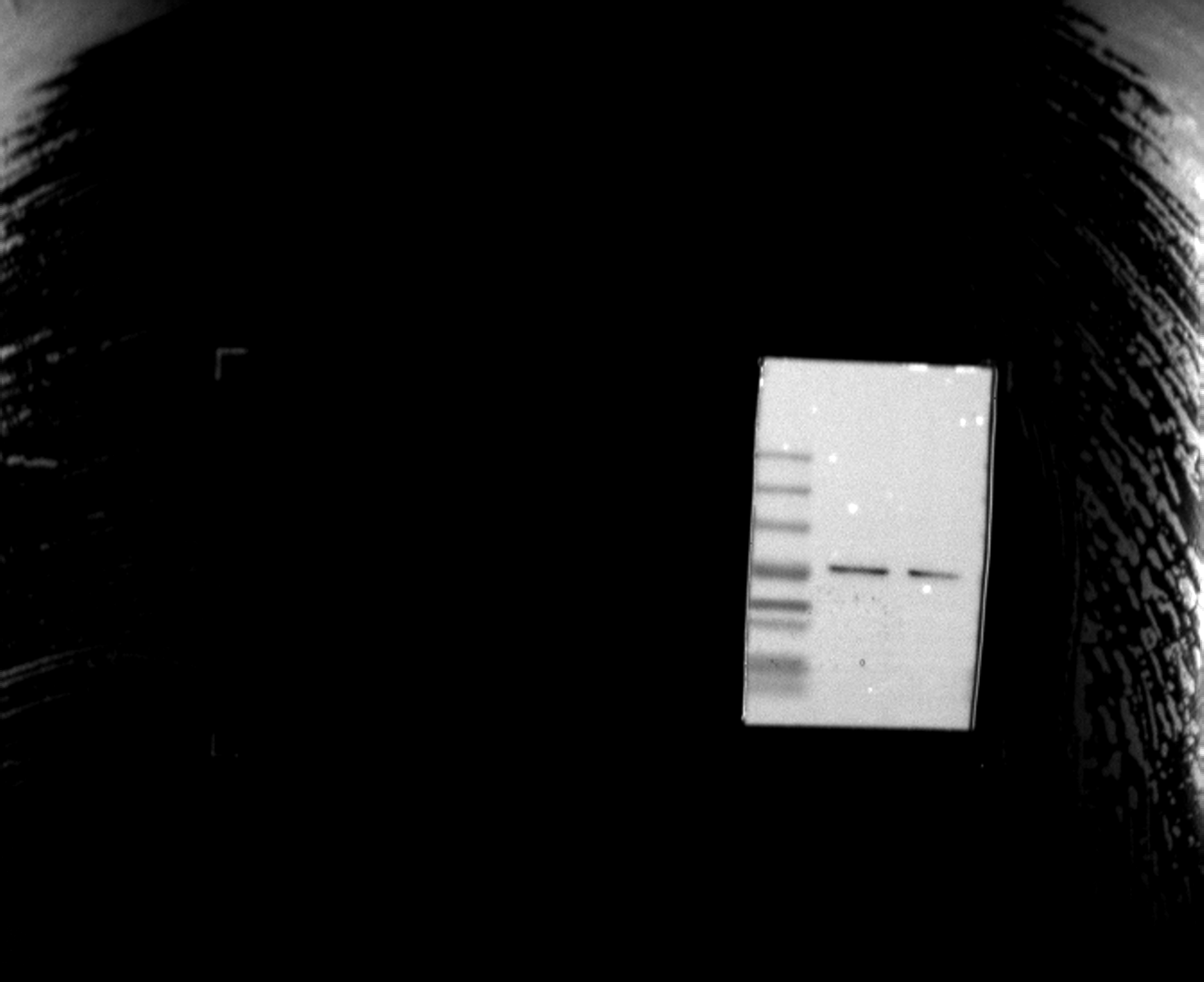

Supplement: Supplementary file 2 [file DataSheet2.zip › original.gel2/NDUFS1 2-2.Tif]

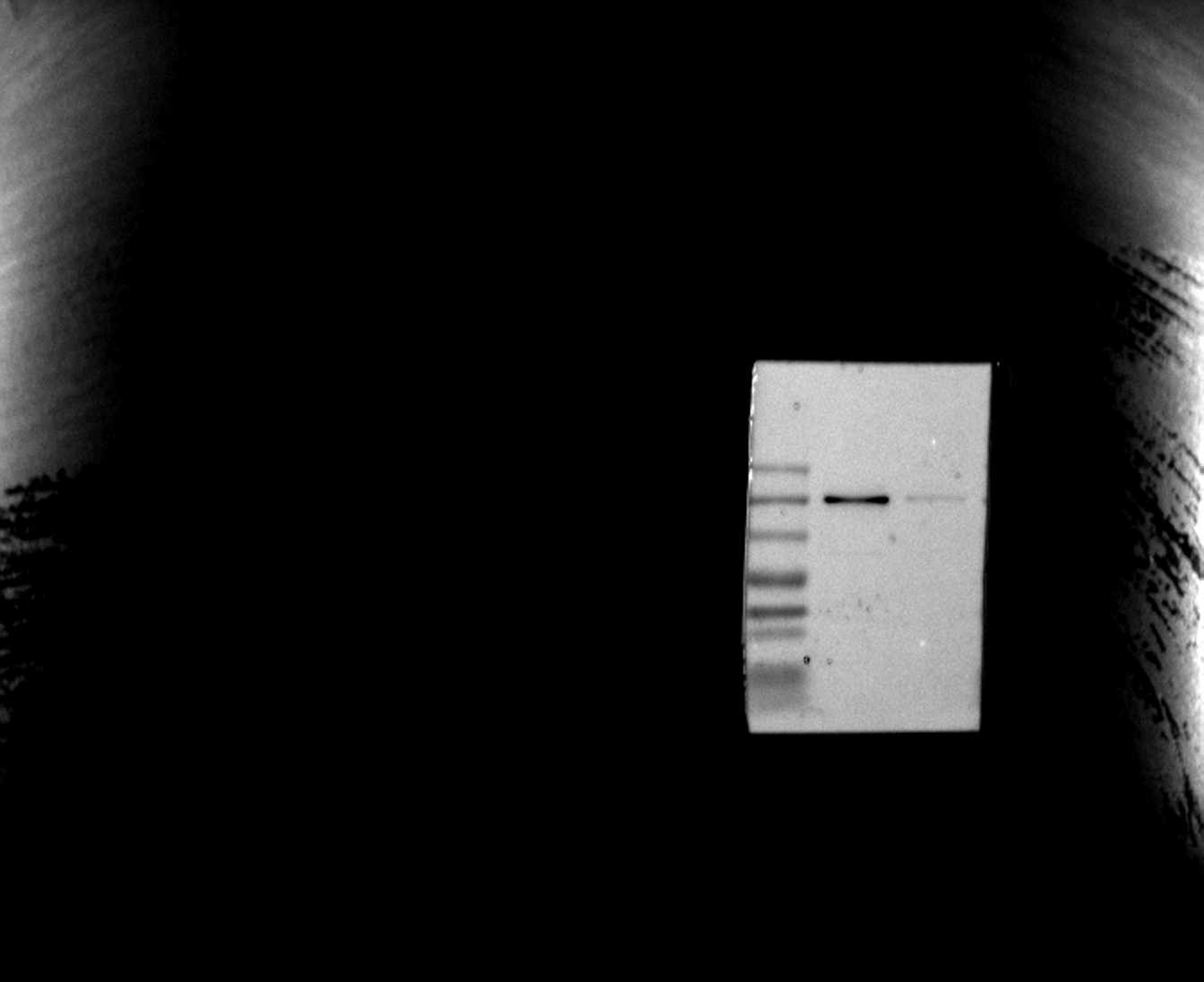

Supplement: Supplementary file 2 [file DataSheet2.zip › original.gel2/LRPRC 2-2.Tif]

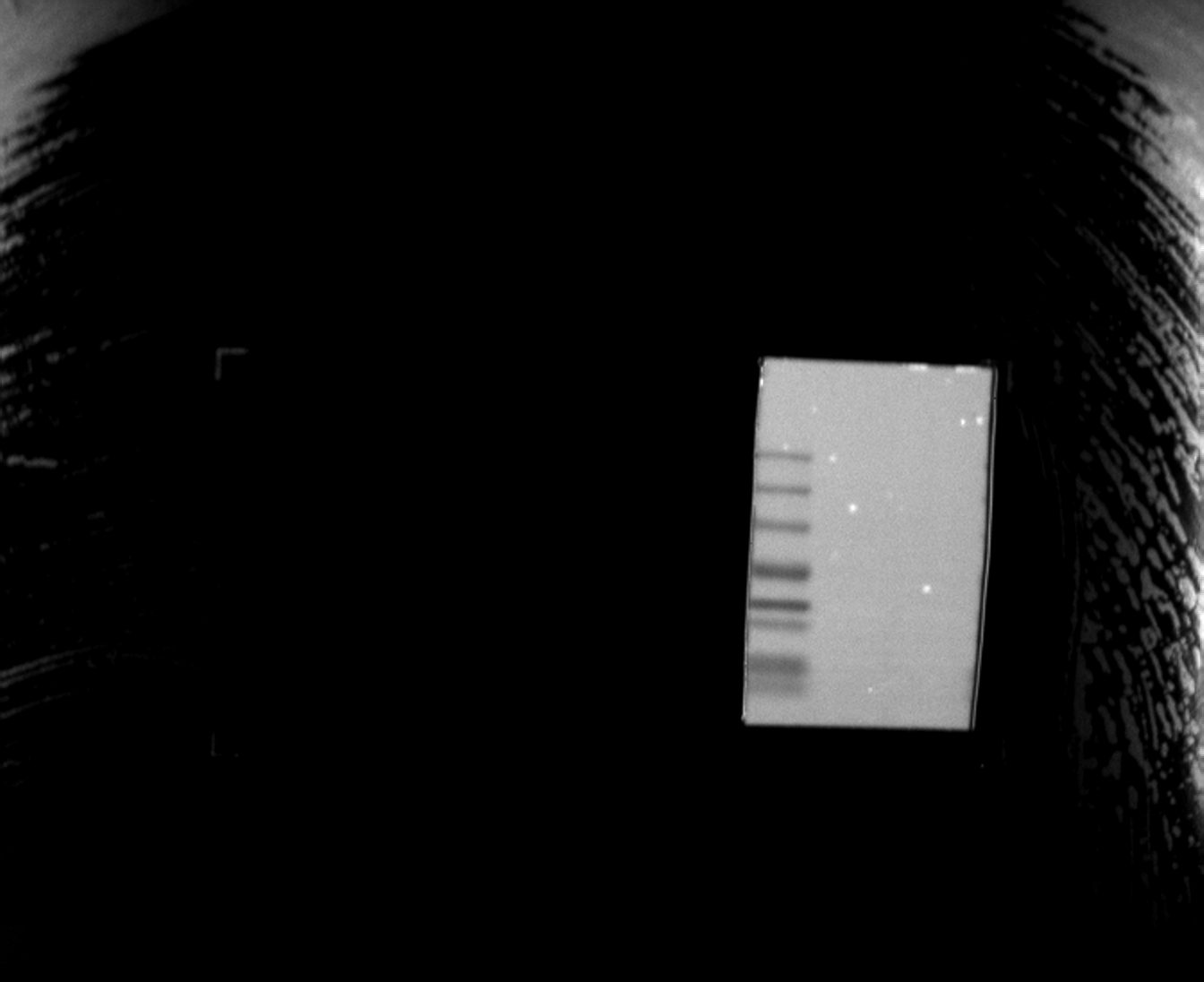

Supplement: Supplementary file 2 [file DataSheet2.zip › original.gel2/NDUFS1 2-1.Tif]

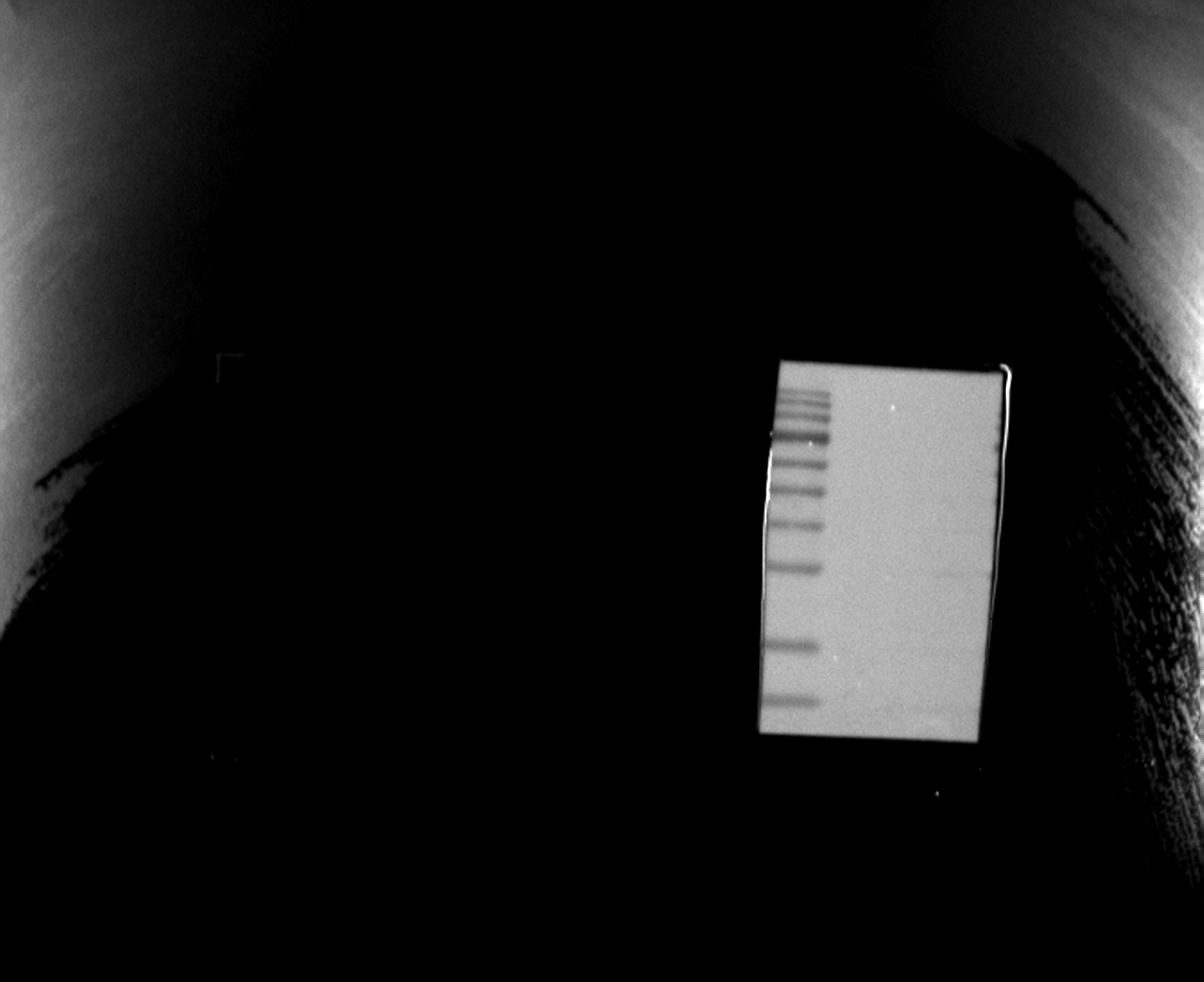

Supplement: Supplementary file 2 [file DataSheet2.zip › original.gel2/GAPDH 2-1.Tif]

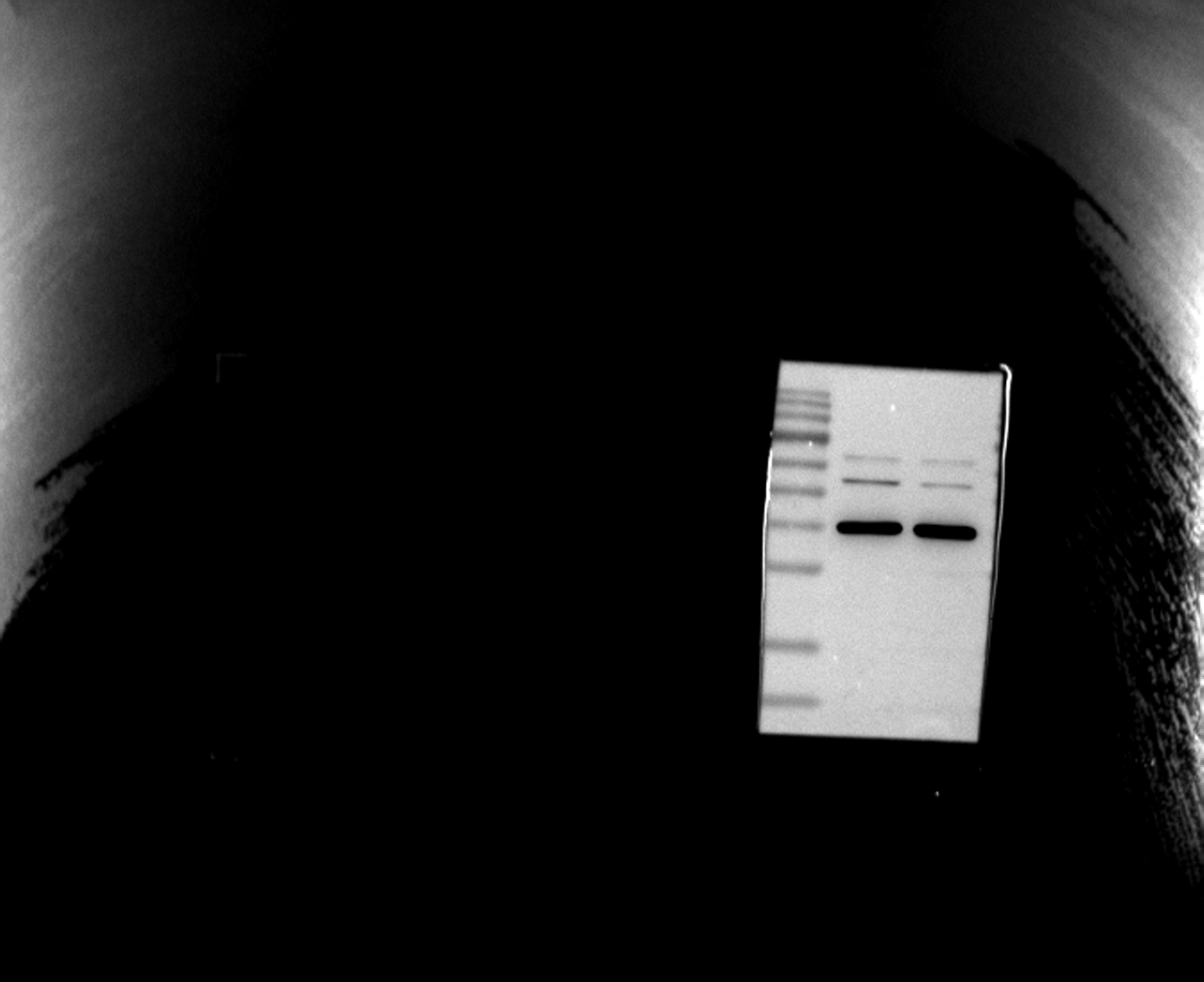

Supplement: Supplementary file 2 [file DataSheet2.zip › original.gel2/GAPDH 2-2.Tif]

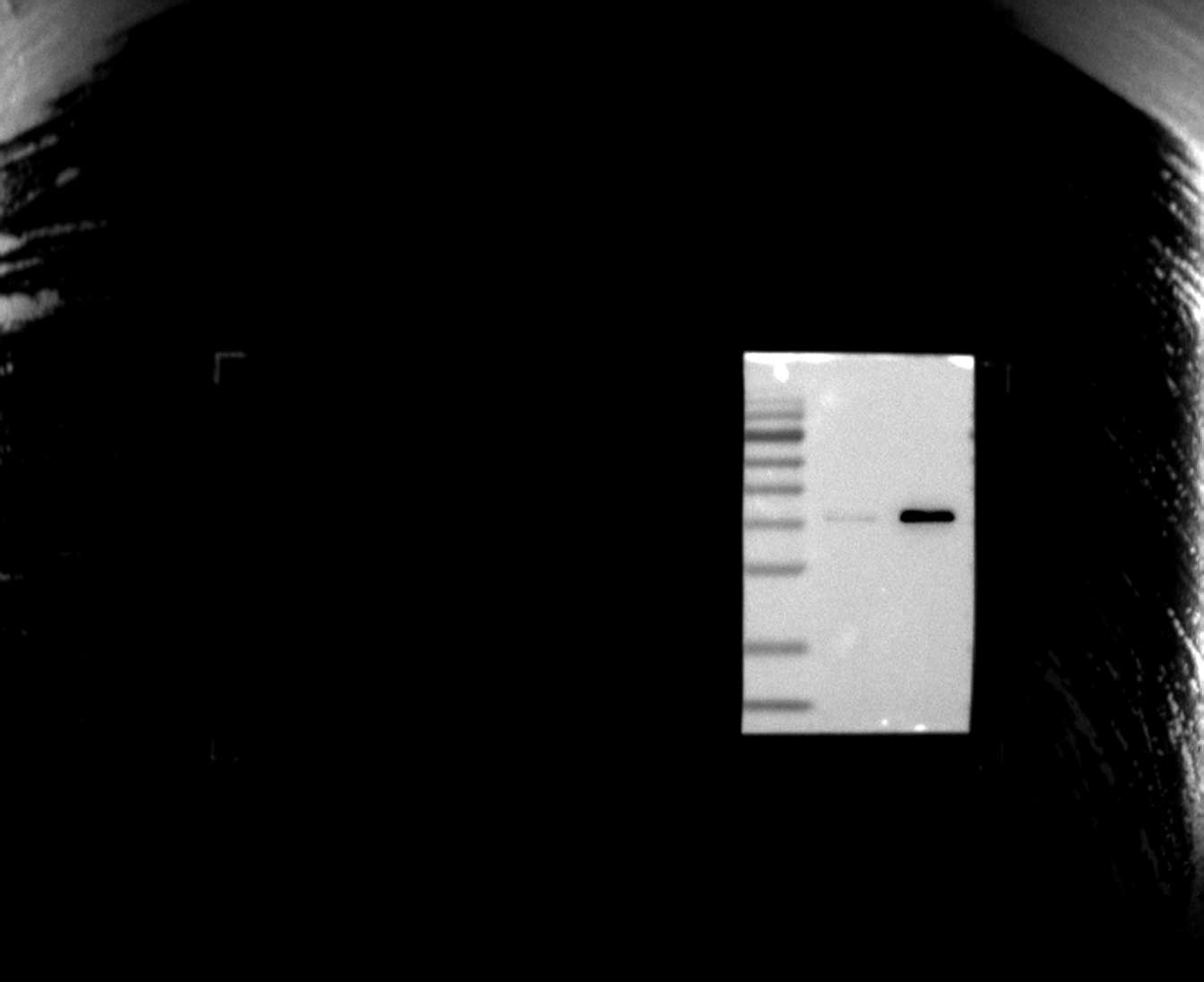

Supplement: Supplementary file 2 [file DataSheet2.zip › original.gel2/NUBPL 2-2.Tif]

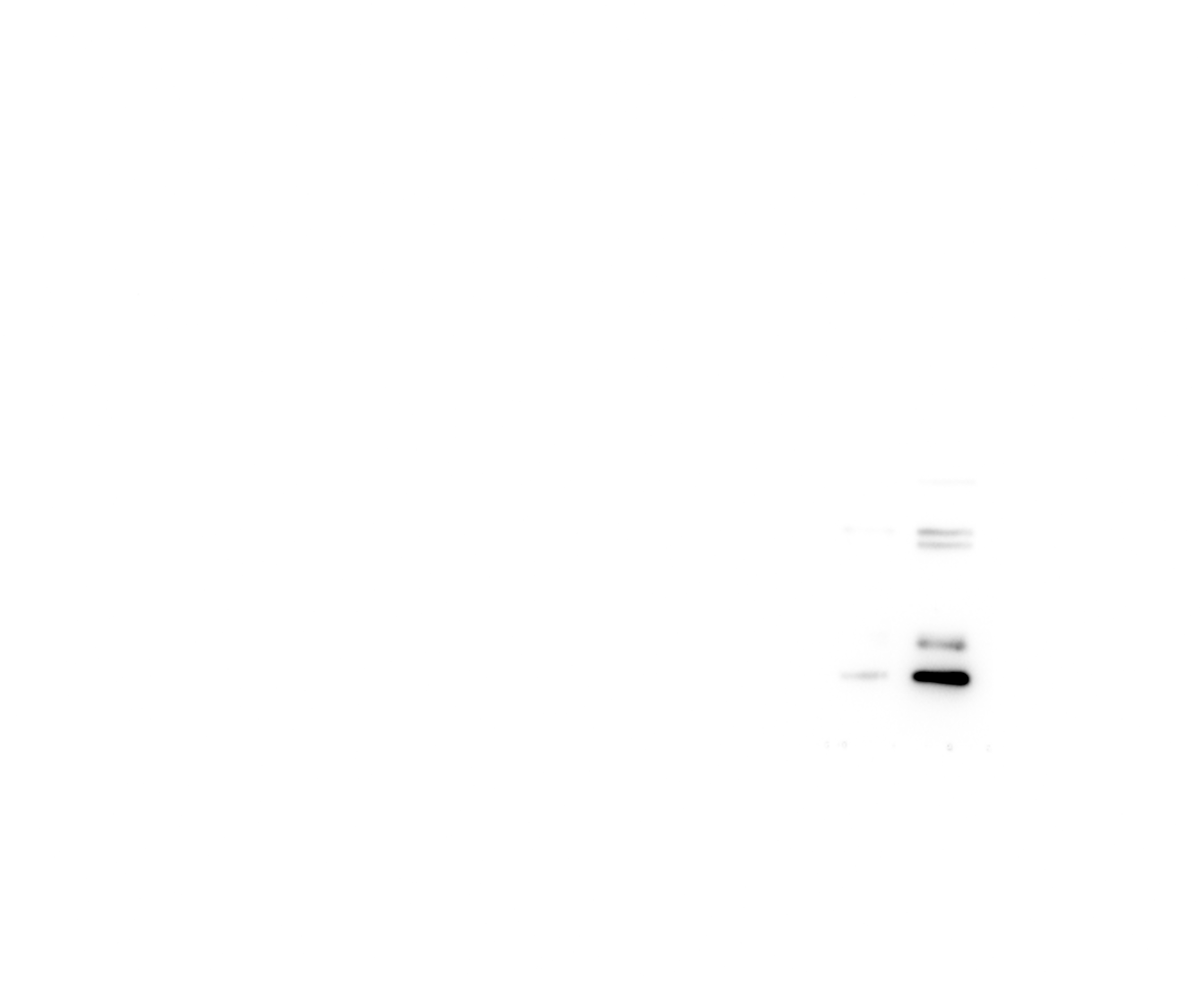

Supplement: Supplementary file 2 [file DataSheet2.zip › original.gel2/NDUFA11 2.Tif]

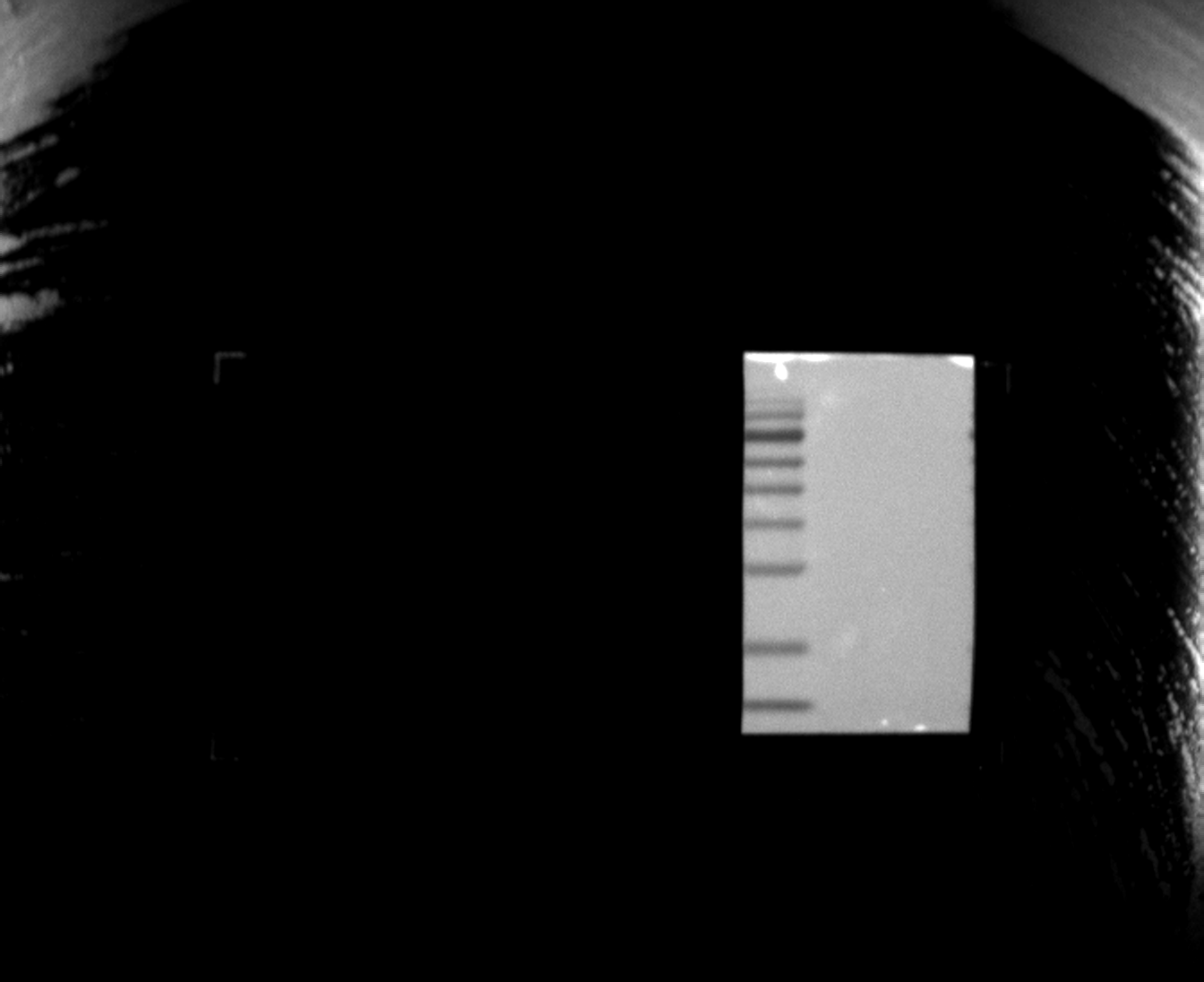

Supplement: Supplementary file 2 [file DataSheet2.zip › original.gel2/NUBPL 2-1.Tif]

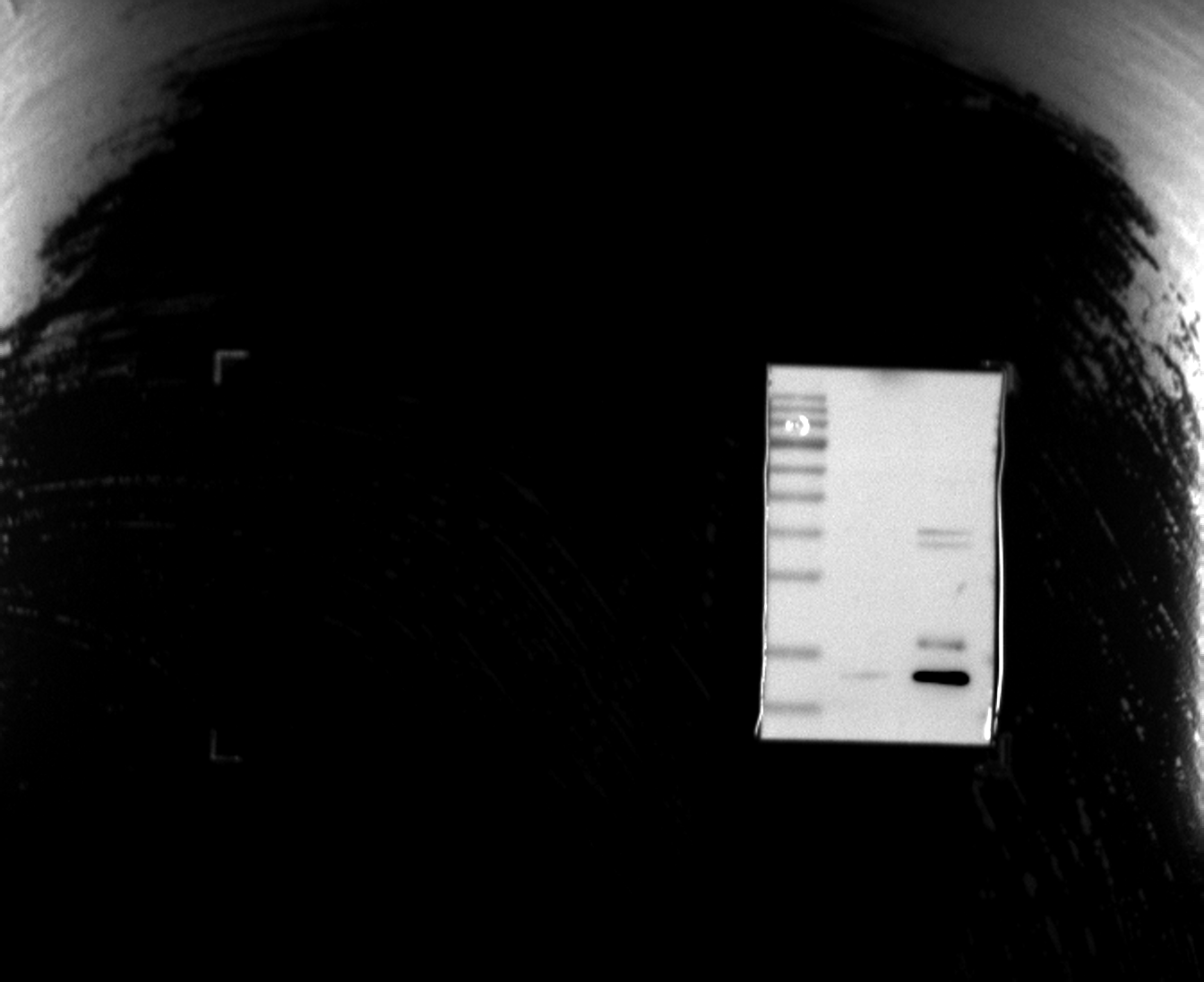

Supplement: Supplementary file 2 [file DataSheet2.zip › original.gel2/NDUFA11 2-2.Tif]

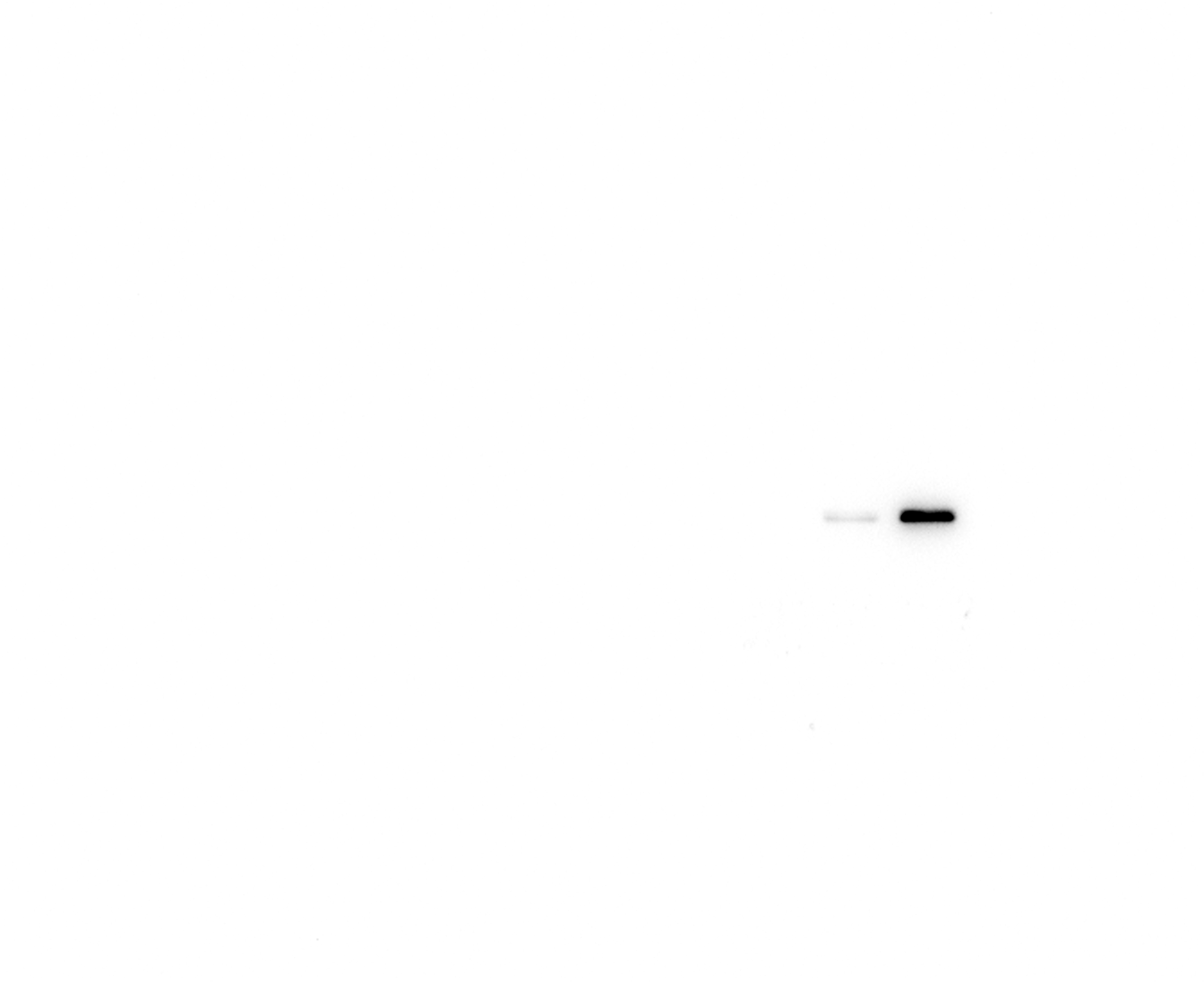

Supplement: Supplementary file 2 [file DataSheet2.zip › original.gel2/NUBPL 2.Tif]

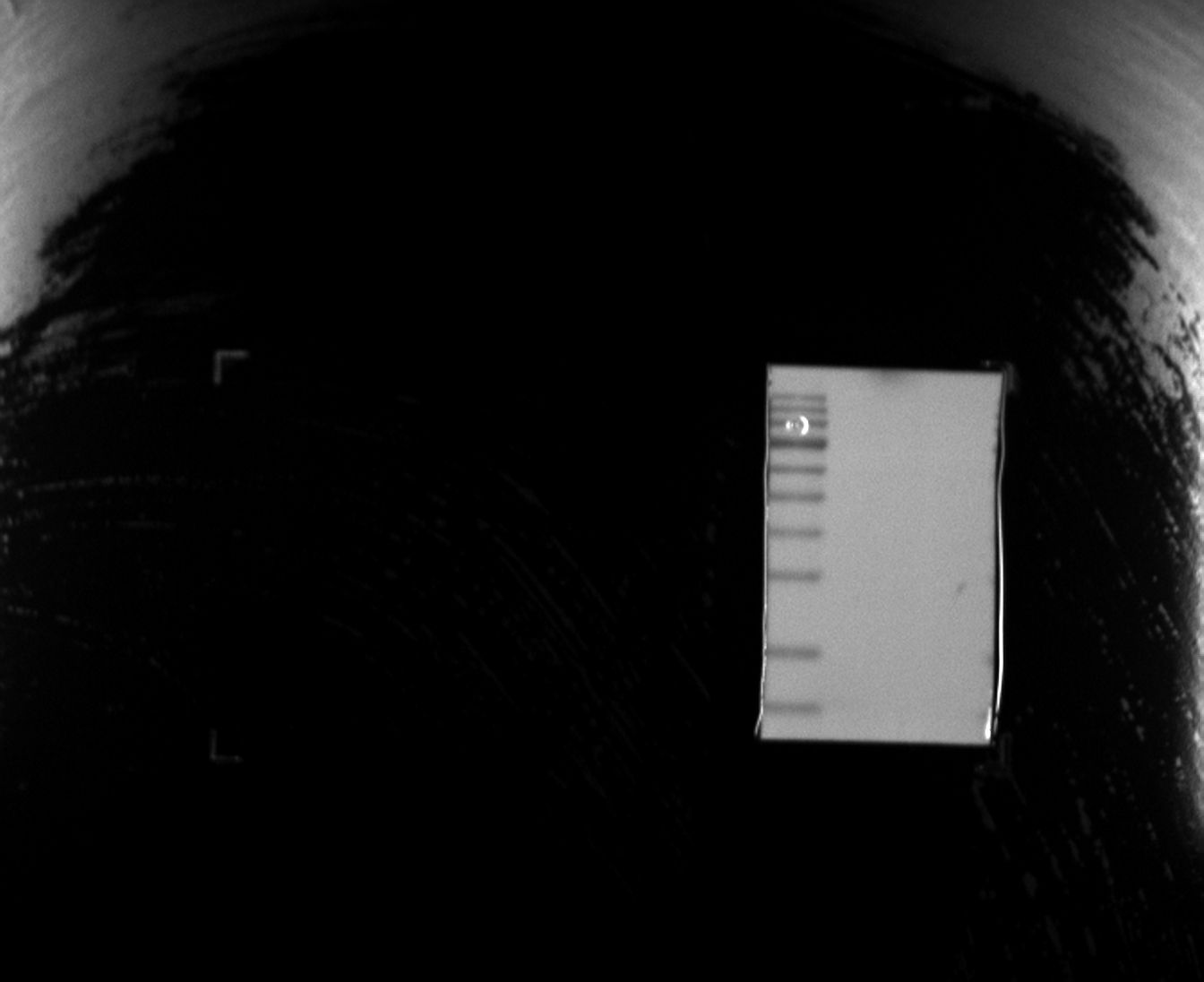

Supplement: Supplementary file 2 [file DataSheet2.zip › original.gel2/NDUFA11 2-1.Tif]

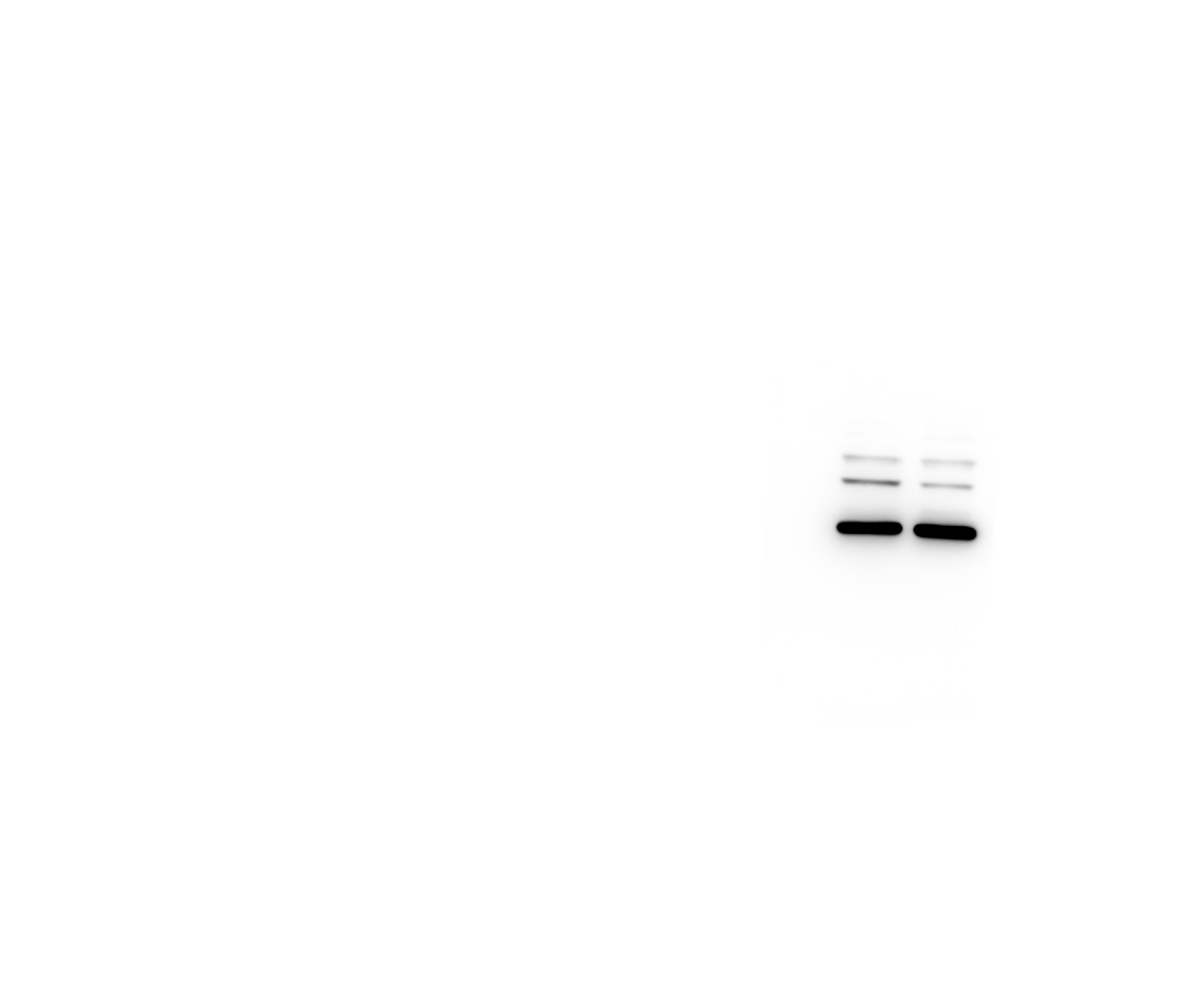

Supplement: Supplementary file 2 [file DataSheet2.zip › original.gel2/GAPDH 2.Tif]

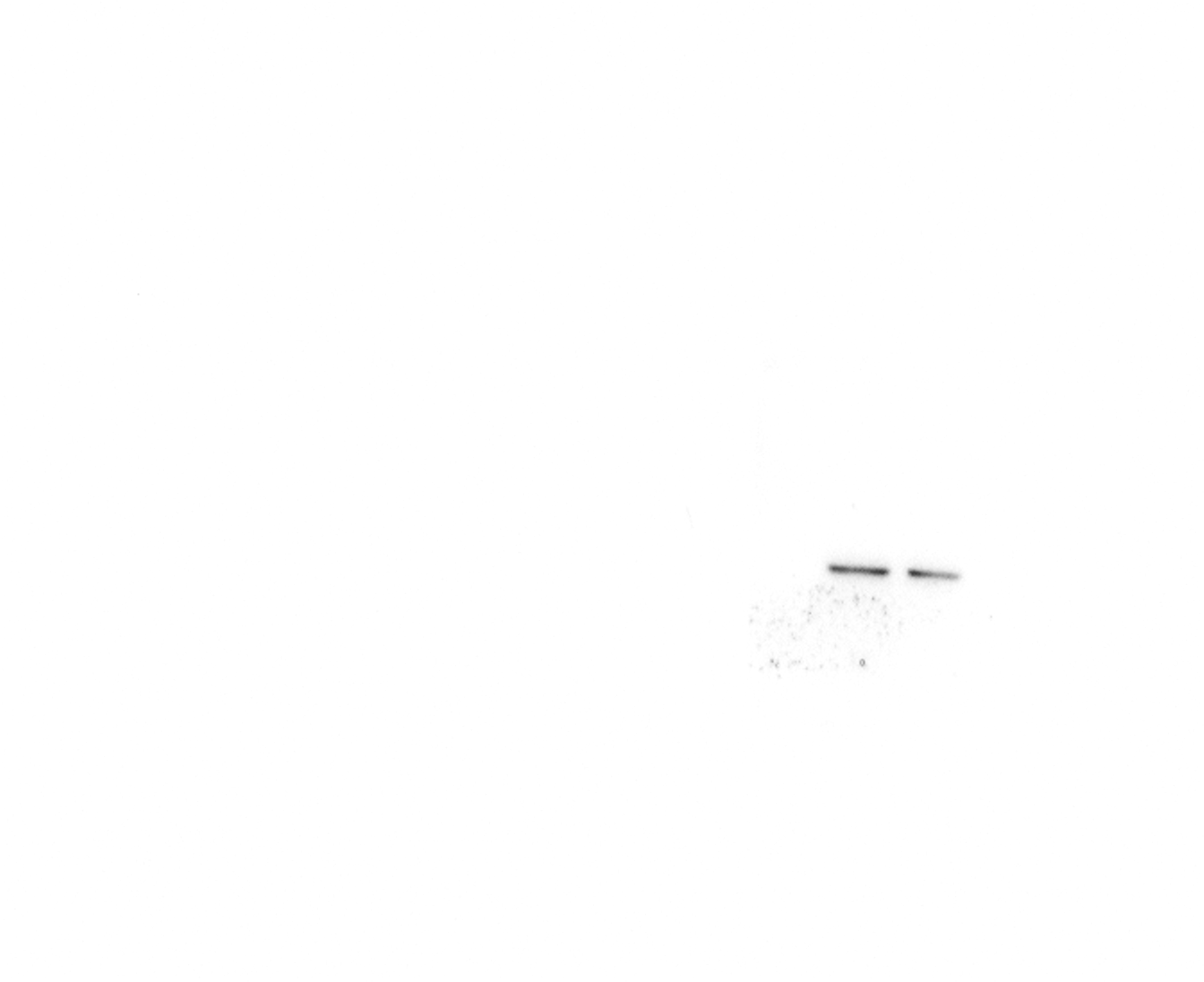

Supplement: Supplementary file 2 [file DataSheet2.zip › original.gel2/NDUFS1 2.Tif]

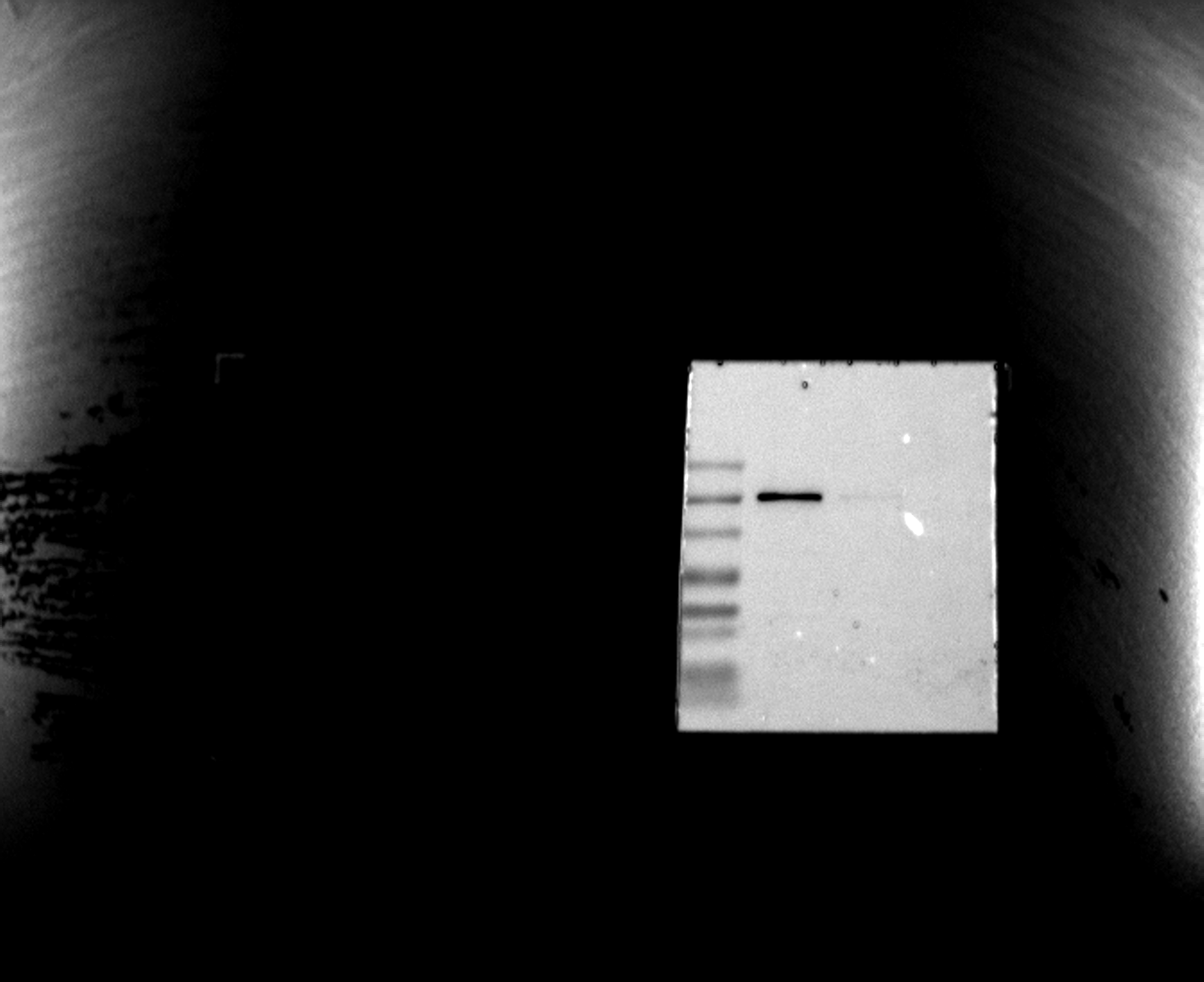

Supplement: Supplementary file 3 [file DataSheet3.zip › original.gel3/LRPRC 3-2.Tif]

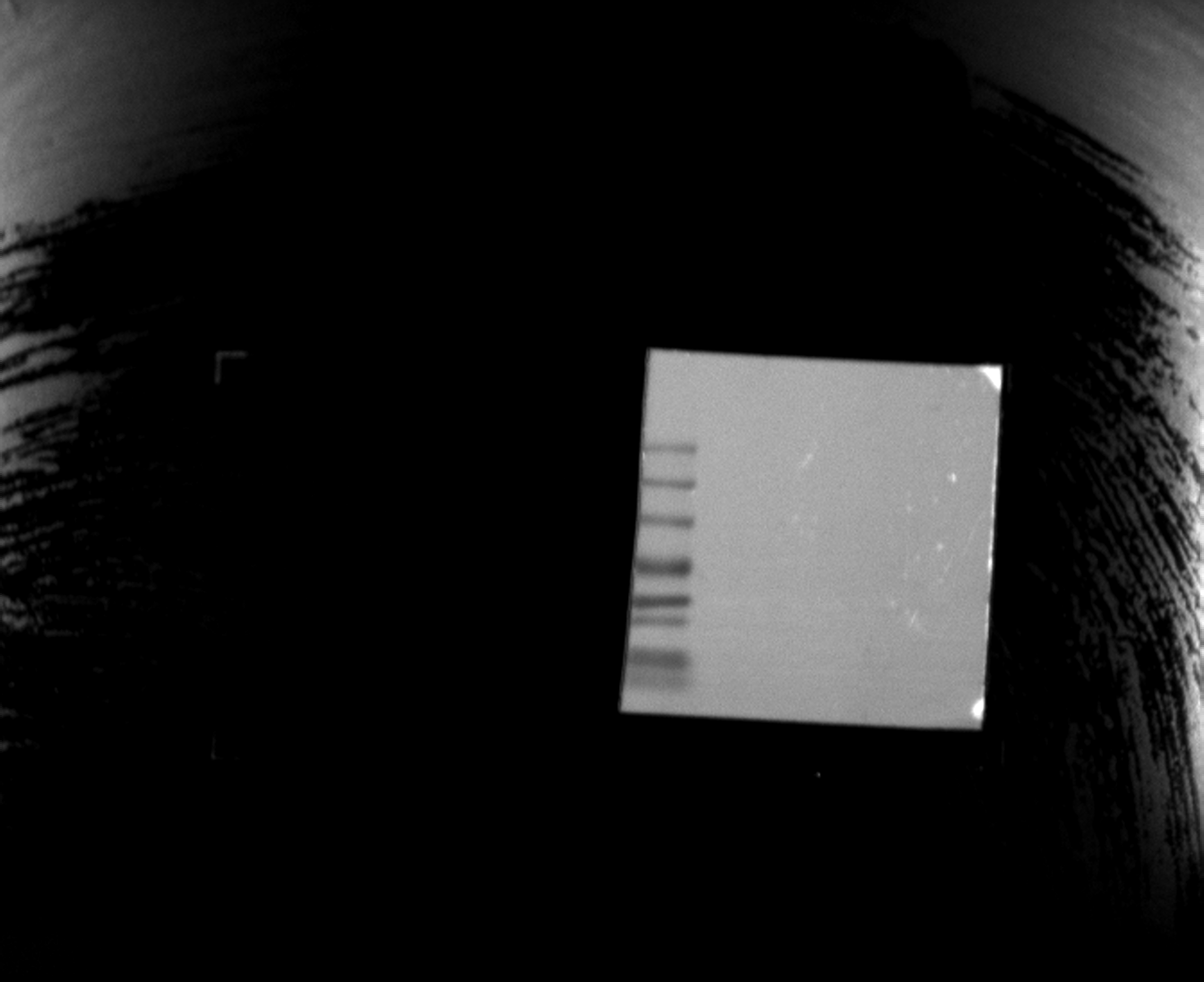

Supplement: Supplementary file 3 [file DataSheet3.zip › original.gel3/NDUFS1 3-1.Tif]

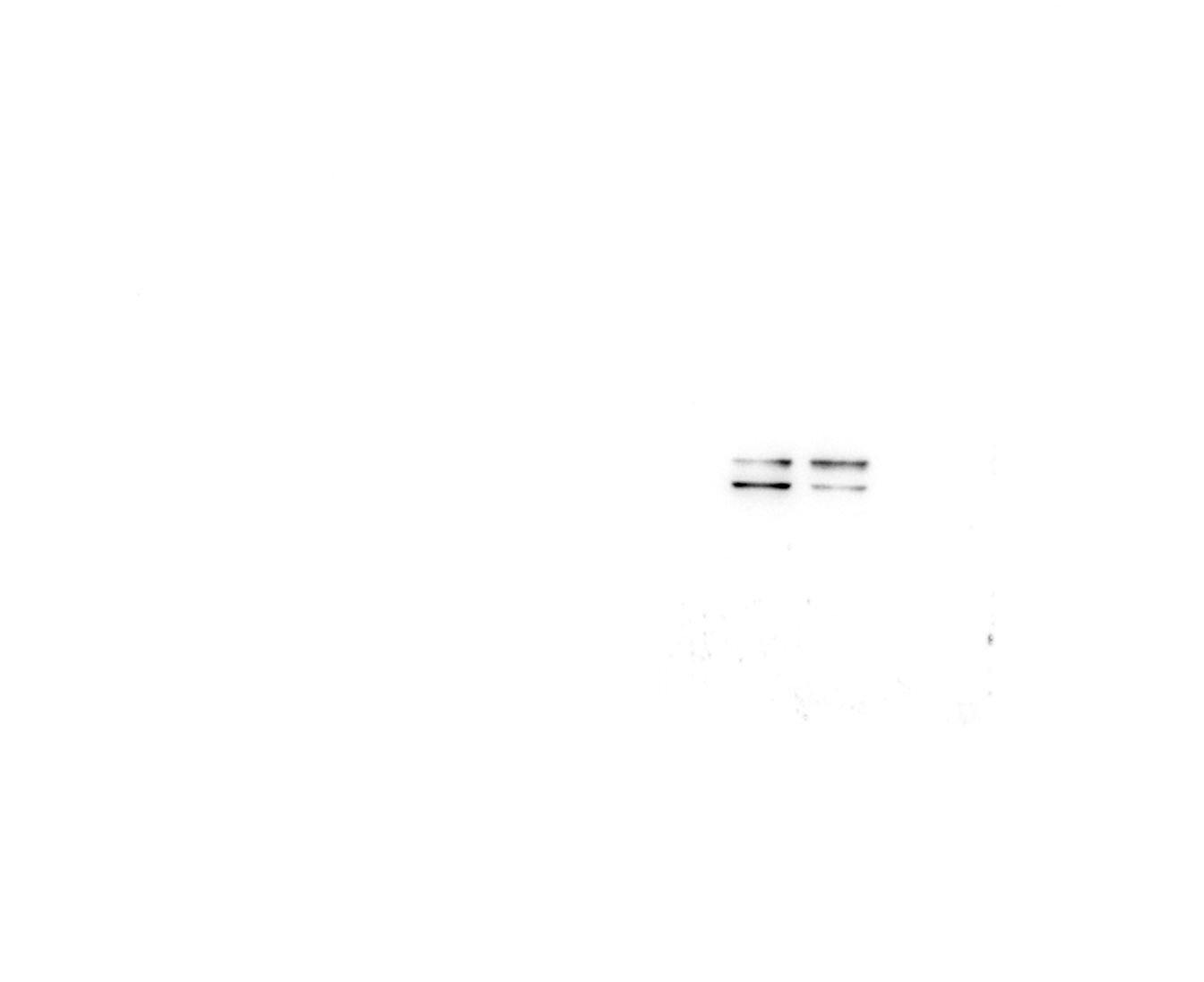

Supplement: Supplementary file 3 [file DataSheet3.zip › original.gel3/OXSM 3.Tif]

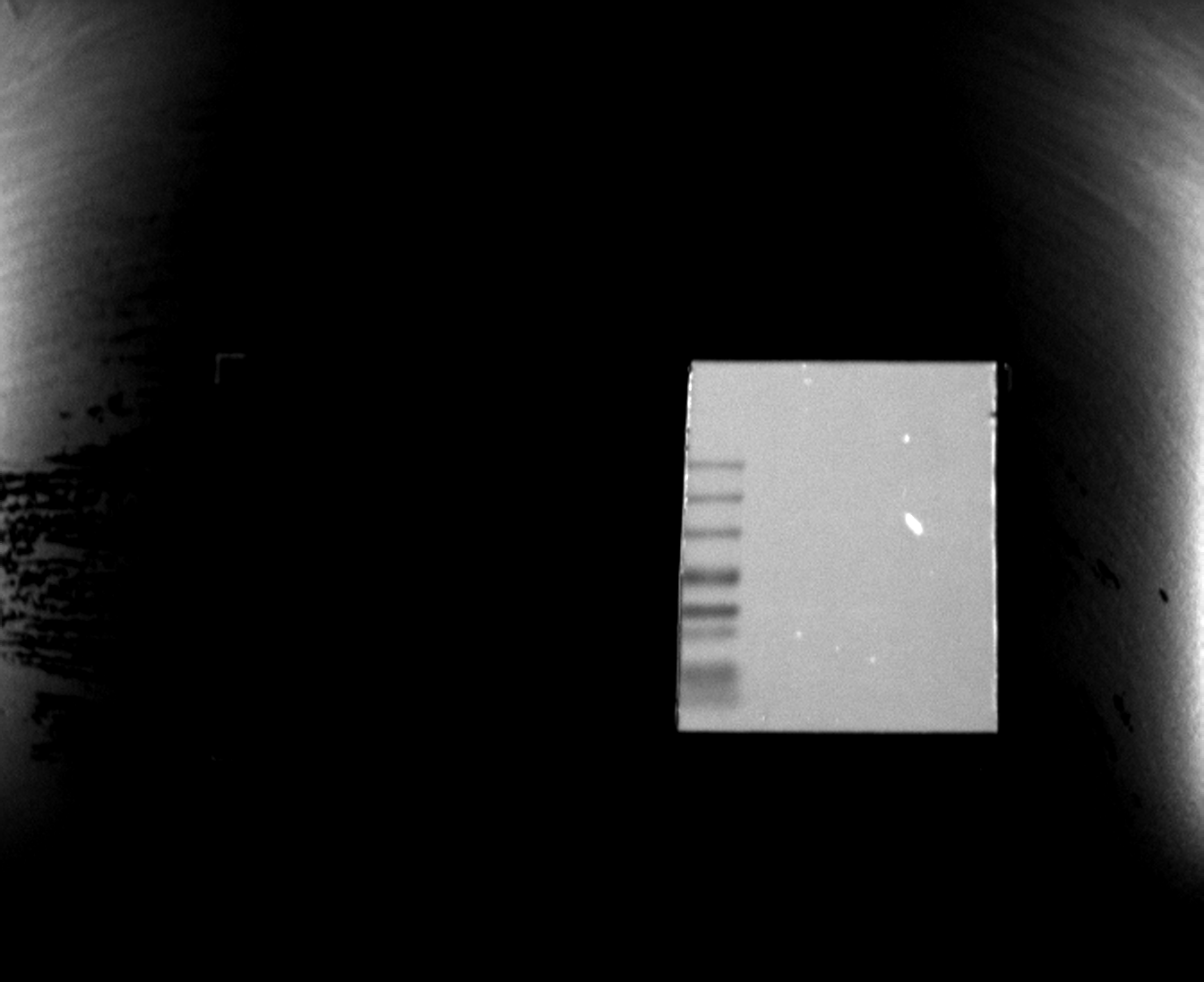

Supplement: Supplementary file 3 [file DataSheet3.zip › original.gel3/LRPRC 3-1.Tif]

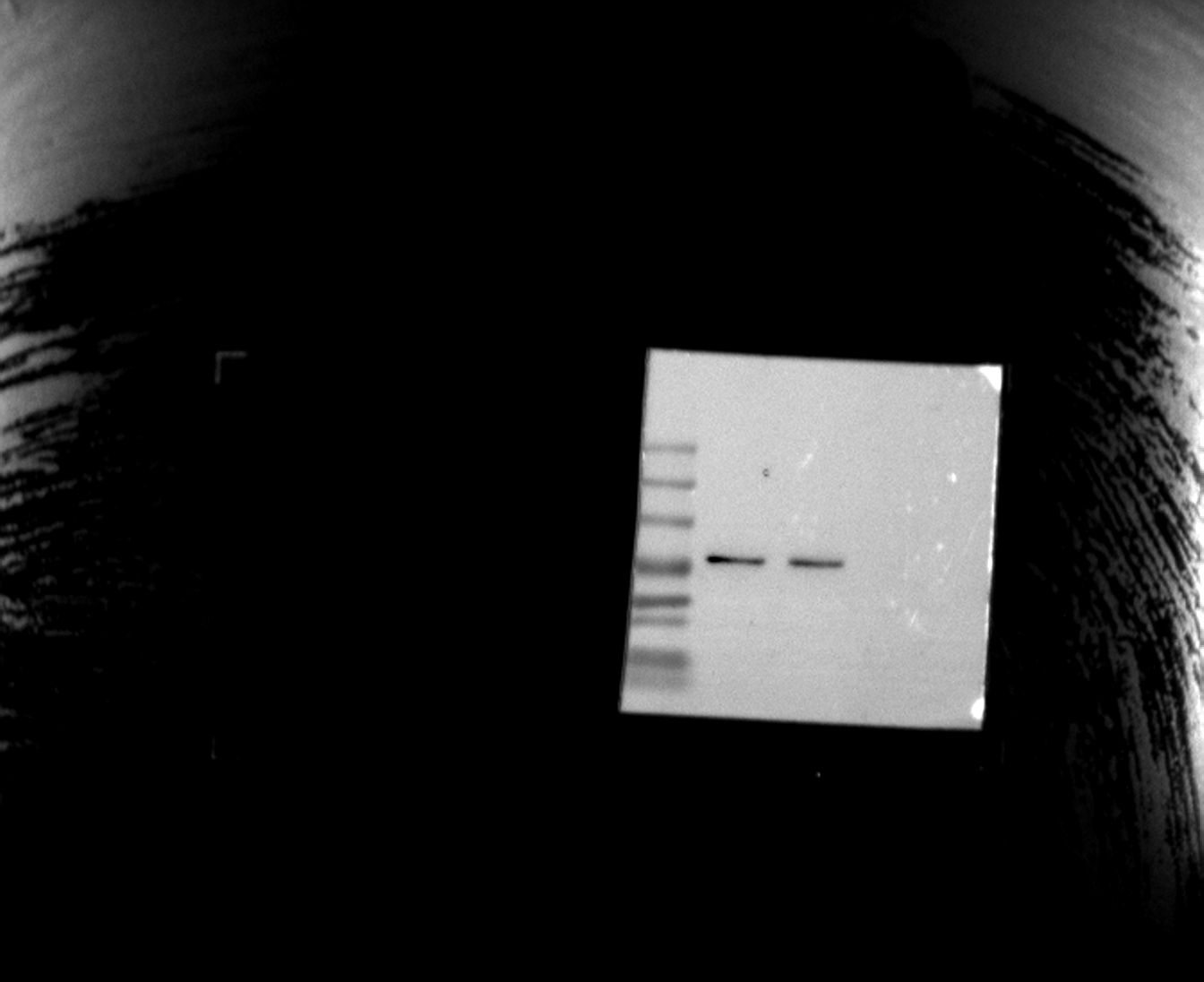

Supplement: Supplementary file 3 [file DataSheet3.zip › original.gel3/NDUFS1 3-2.Tif]

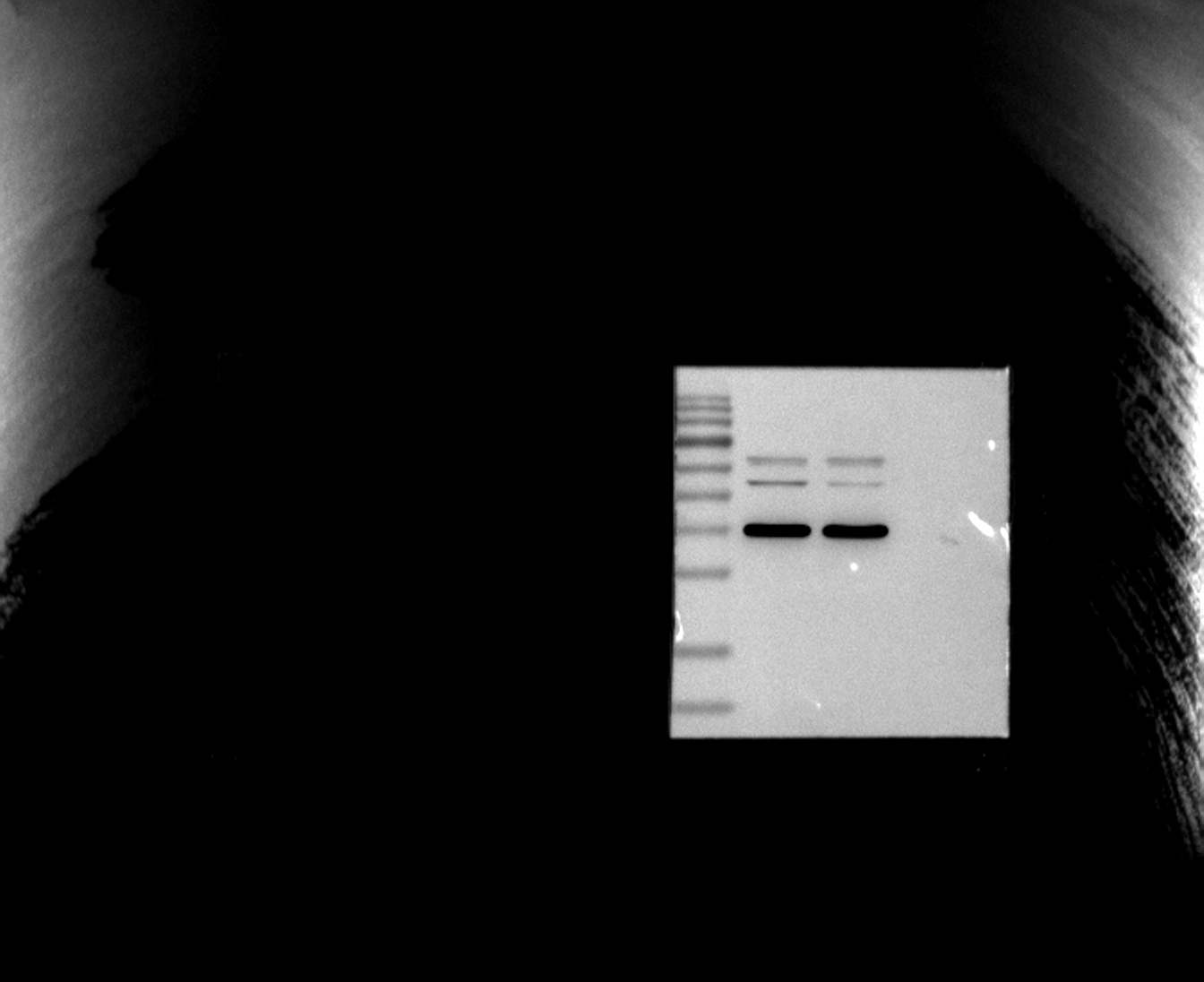

Supplement: Supplementary file 3 [file DataSheet3.zip › original.gel3/GAPDH 3-2.Tif]

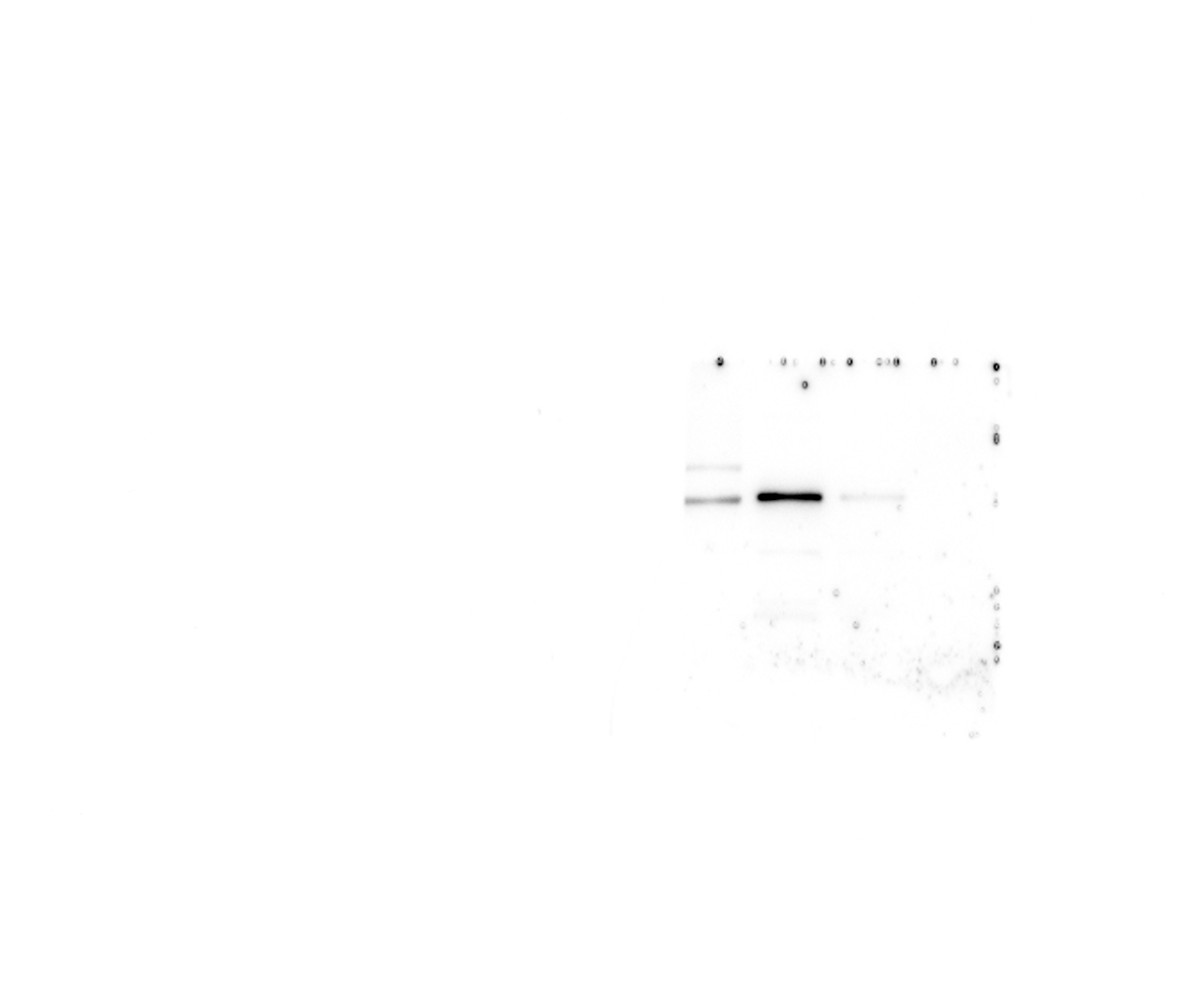

Supplement: Supplementary file 3 [file DataSheet3.zip › original.gel3/LRPRC 3.Tif]

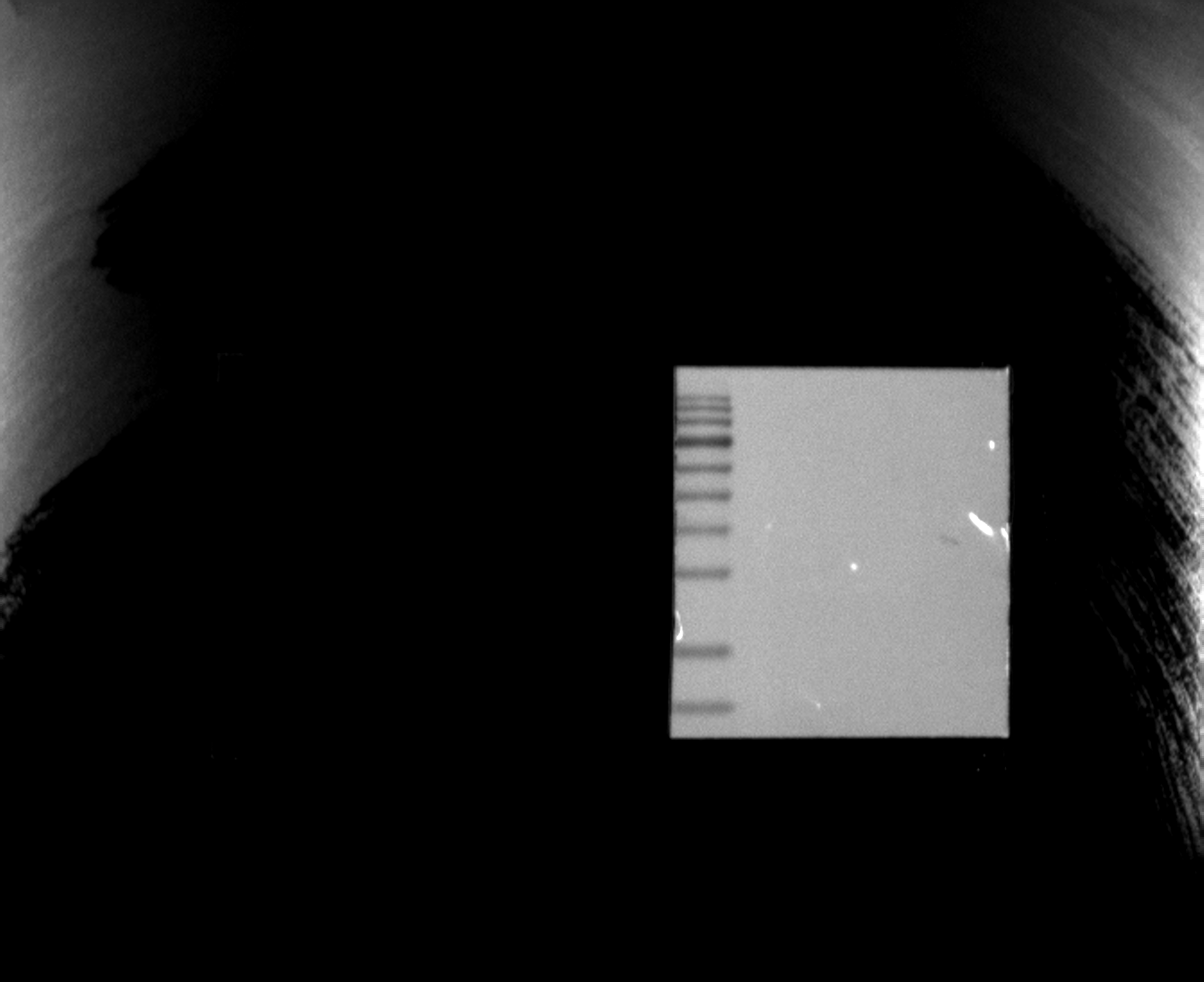

Supplement: Supplementary file 3 [file DataSheet3.zip › original.gel3/GAPDH 3-1.Tif]

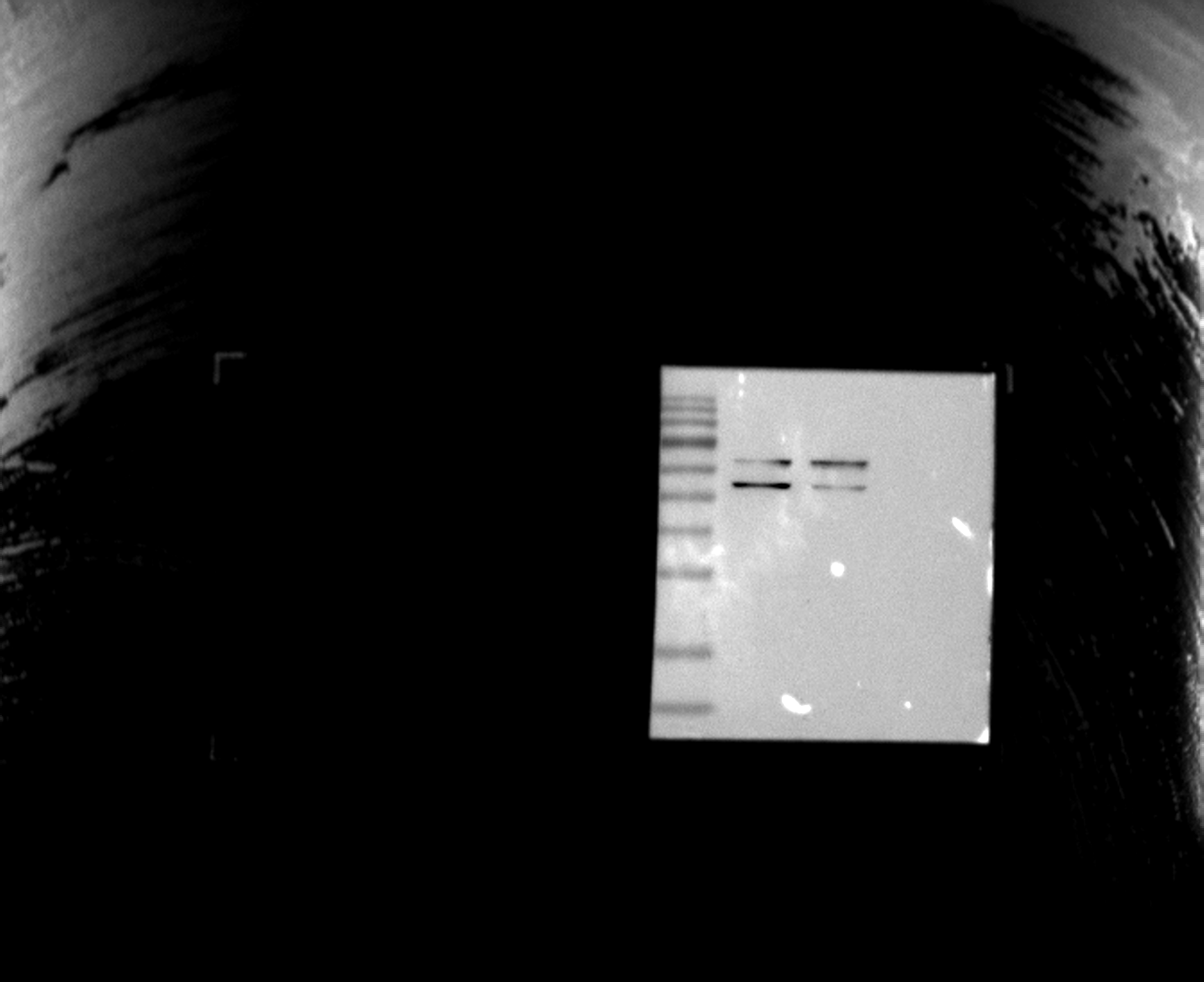

Supplement: Supplementary file 3 [file DataSheet3.zip › original.gel3/OXSM 3-2.Tif]

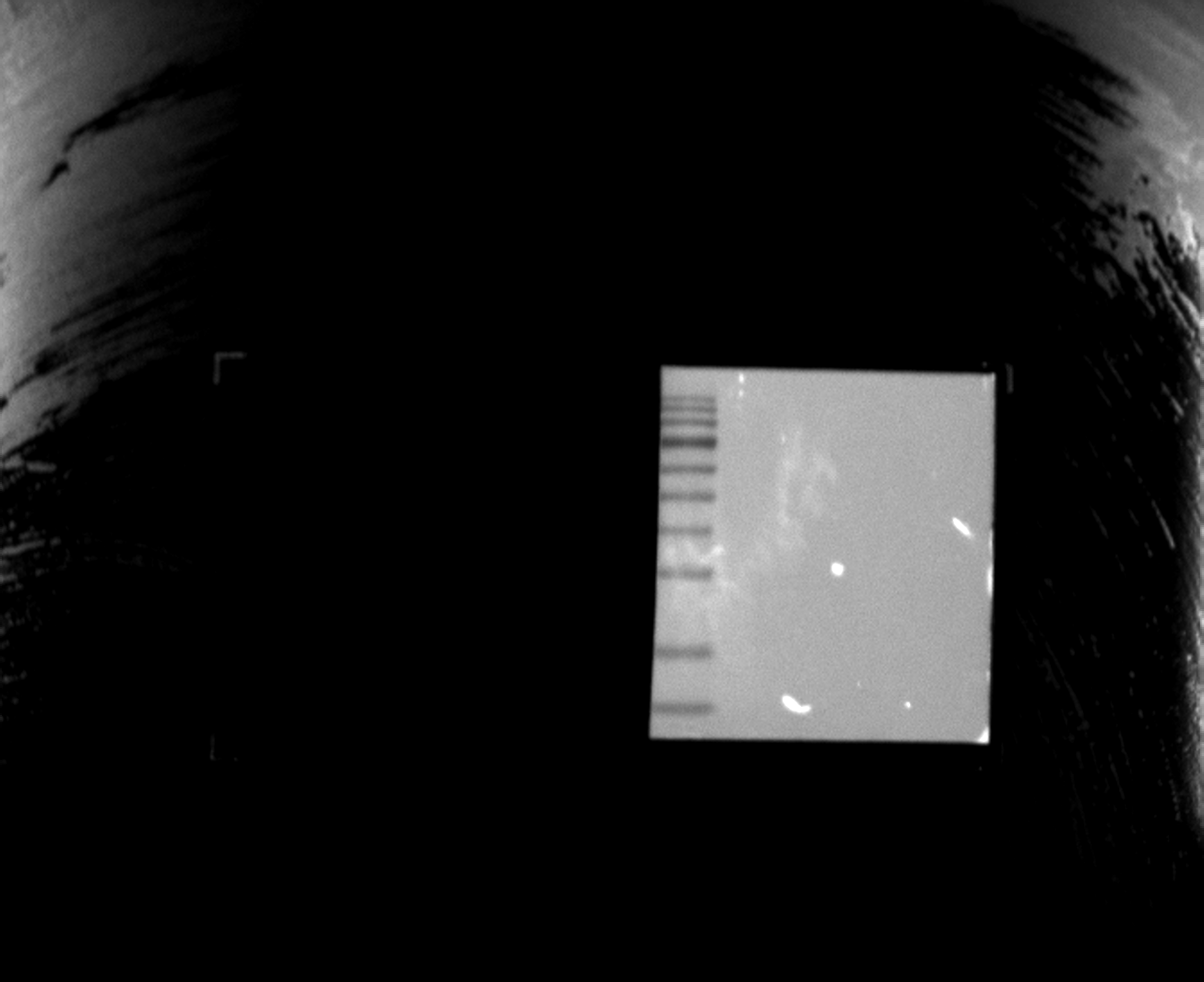

Supplement: Supplementary file 3 [file DataSheet3.zip › original.gel3/OXSM 3-1.Tif]

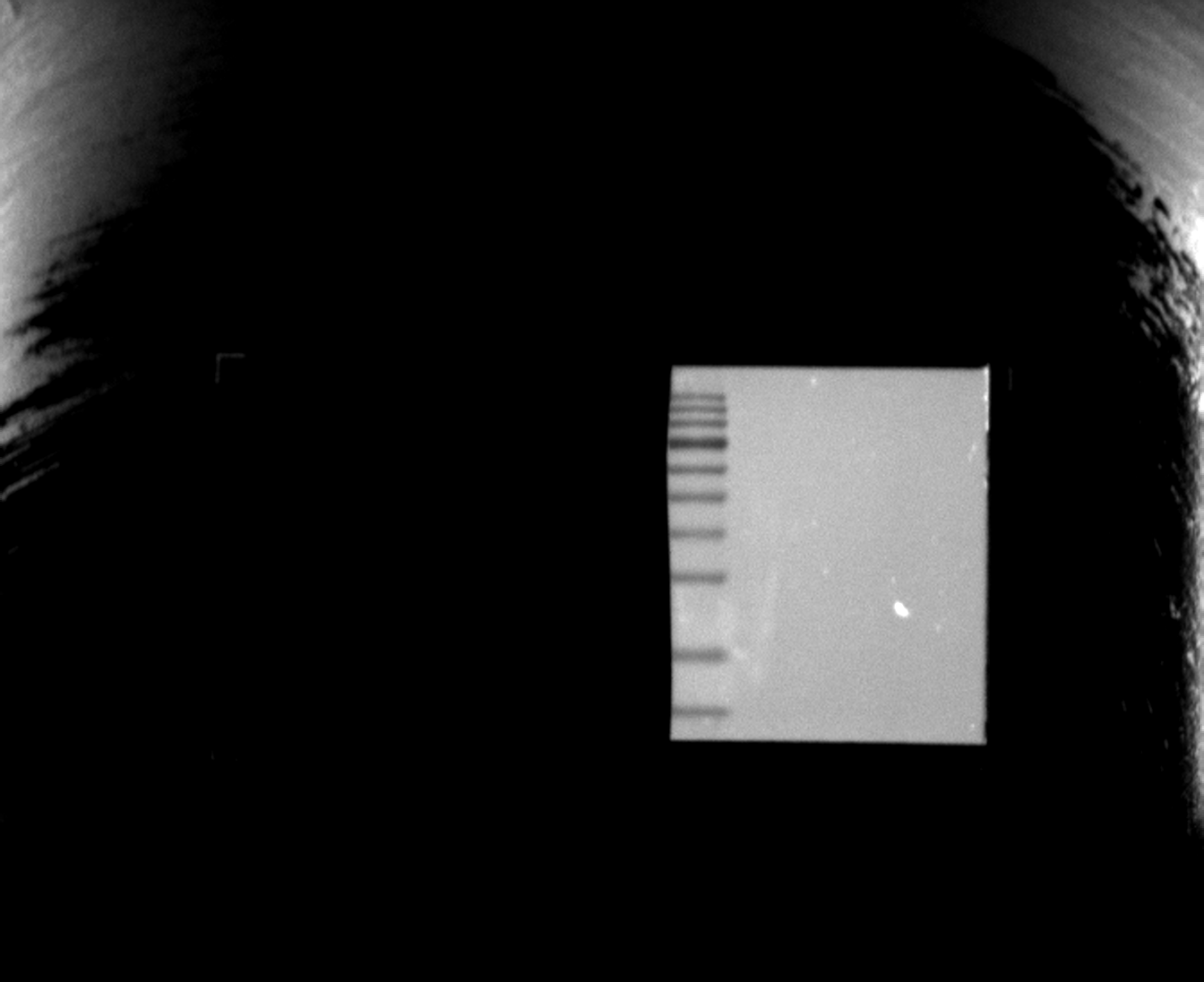

Supplement: Supplementary file 3 [file DataSheet3.zip › original.gel3/NDUFA11 3-1.Tif]

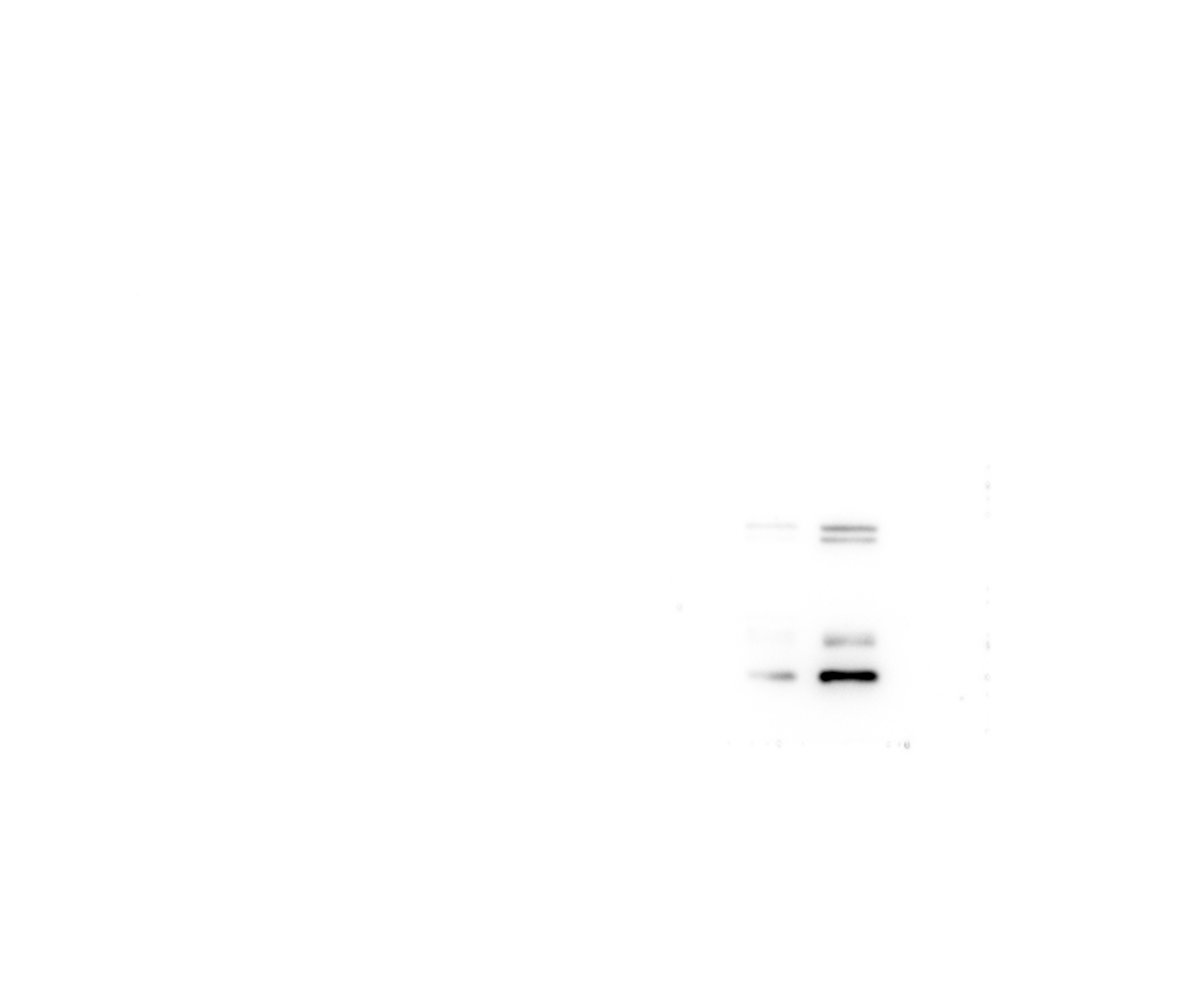

Supplement: Supplementary file 3 [file DataSheet3.zip › original.gel3/NDUFA11 3.Tif]

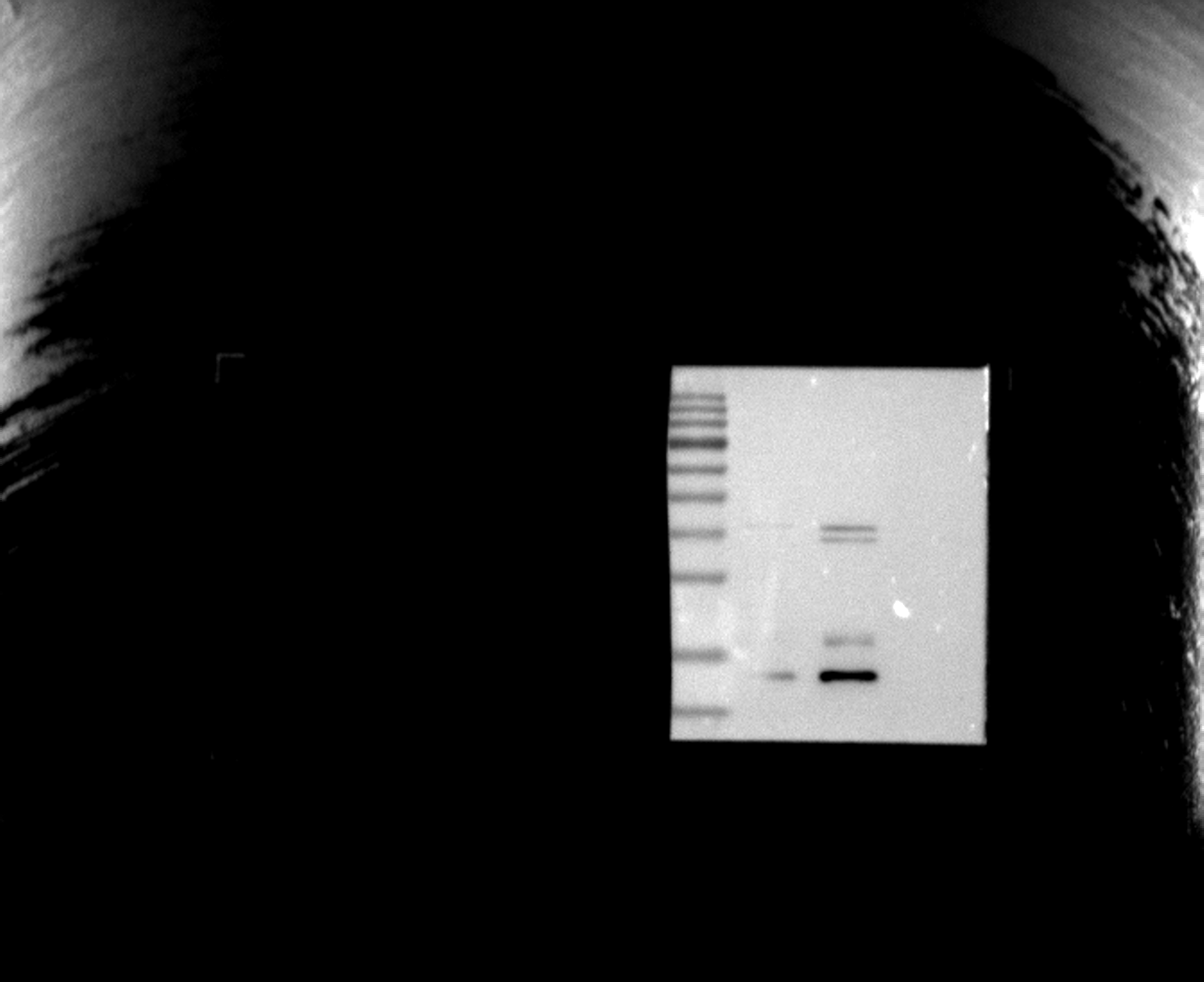

Supplement: Supplementary file 3 [file DataSheet3.zip › original.gel3/NDUFA11 3-2.Tif]

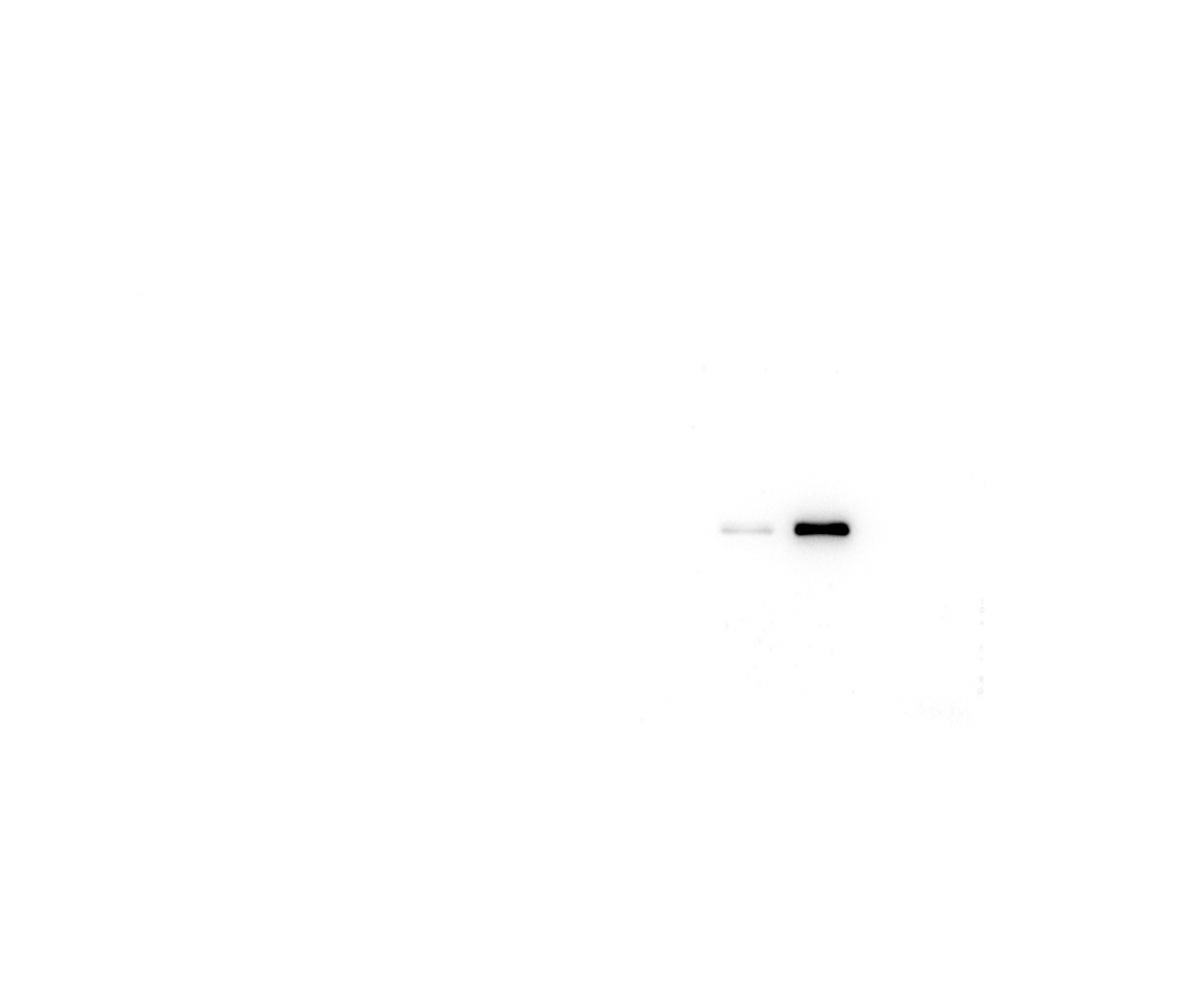

Supplement: Supplementary file 3 [file DataSheet3.zip › original.gel3/NUBPL 3.Tif]

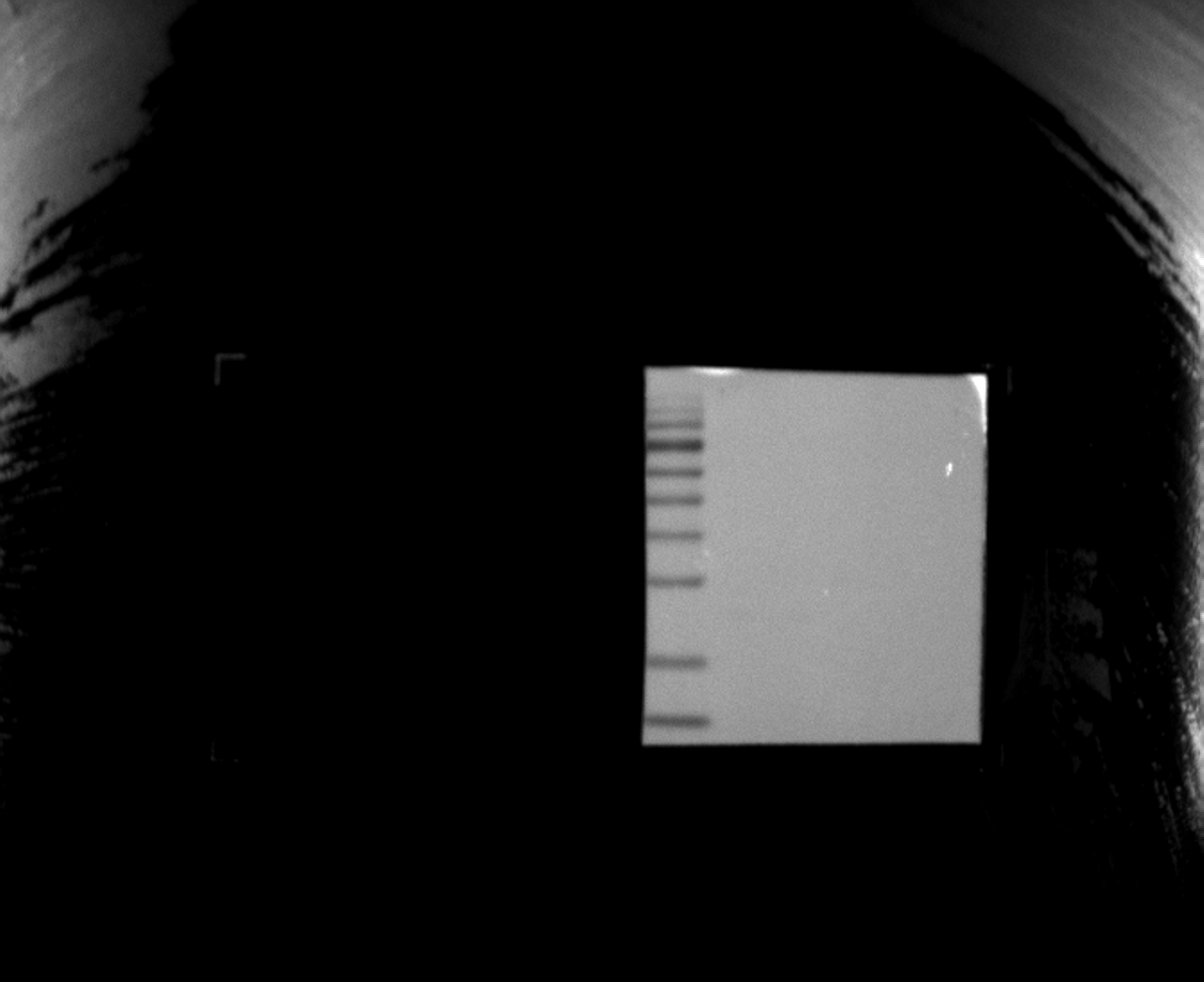

Supplement: Supplementary file 3 [file DataSheet3.zip › original.gel3/NUBPL 3-1.Tif]

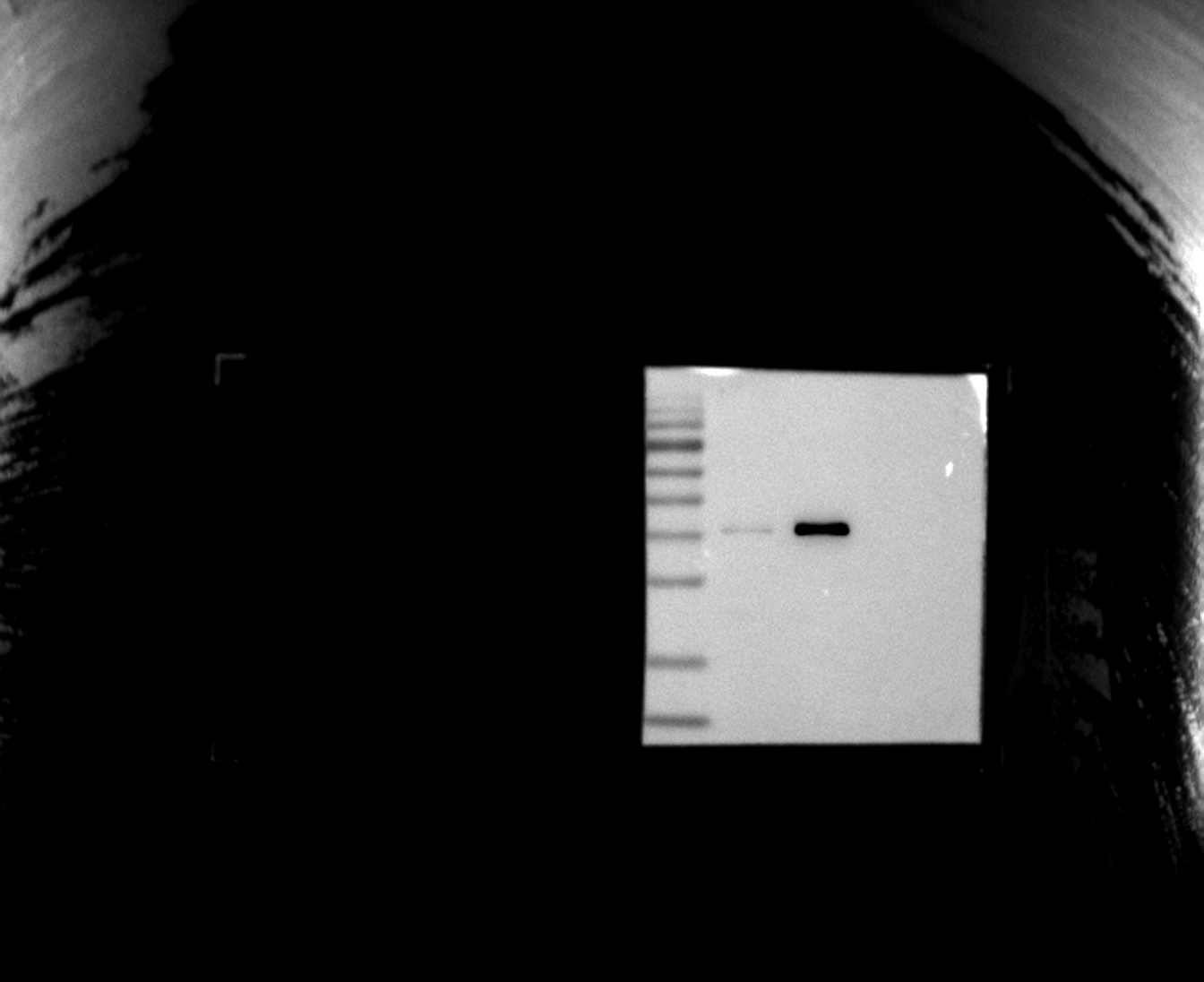

Supplement: Supplementary file 3 [file DataSheet3.zip › original.gel3/NUBPL 3-2.Tif]

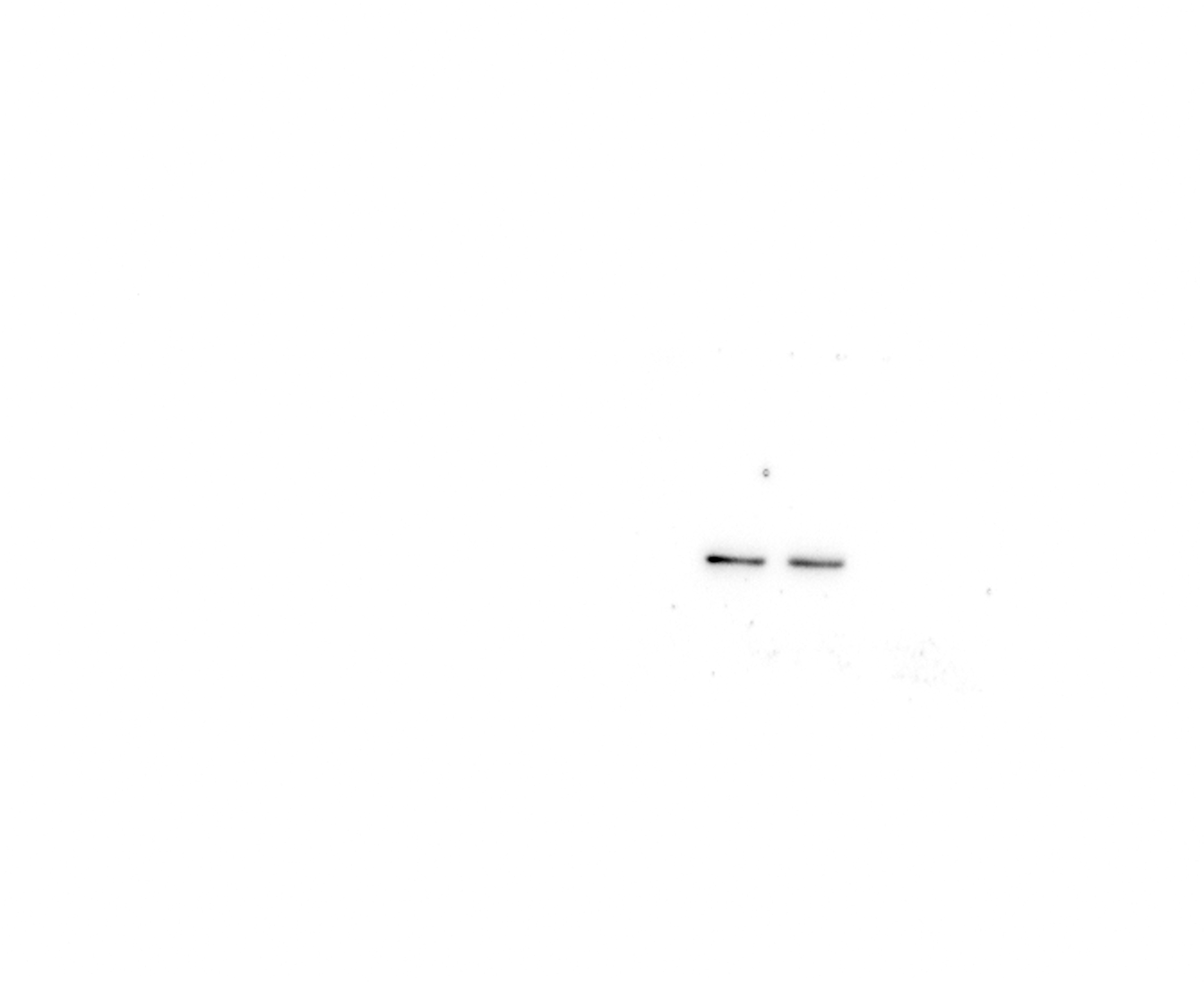

Supplement: Supplementary file 3 [file DataSheet3.zip › original.gel3/NDUFS1 3.Tif]

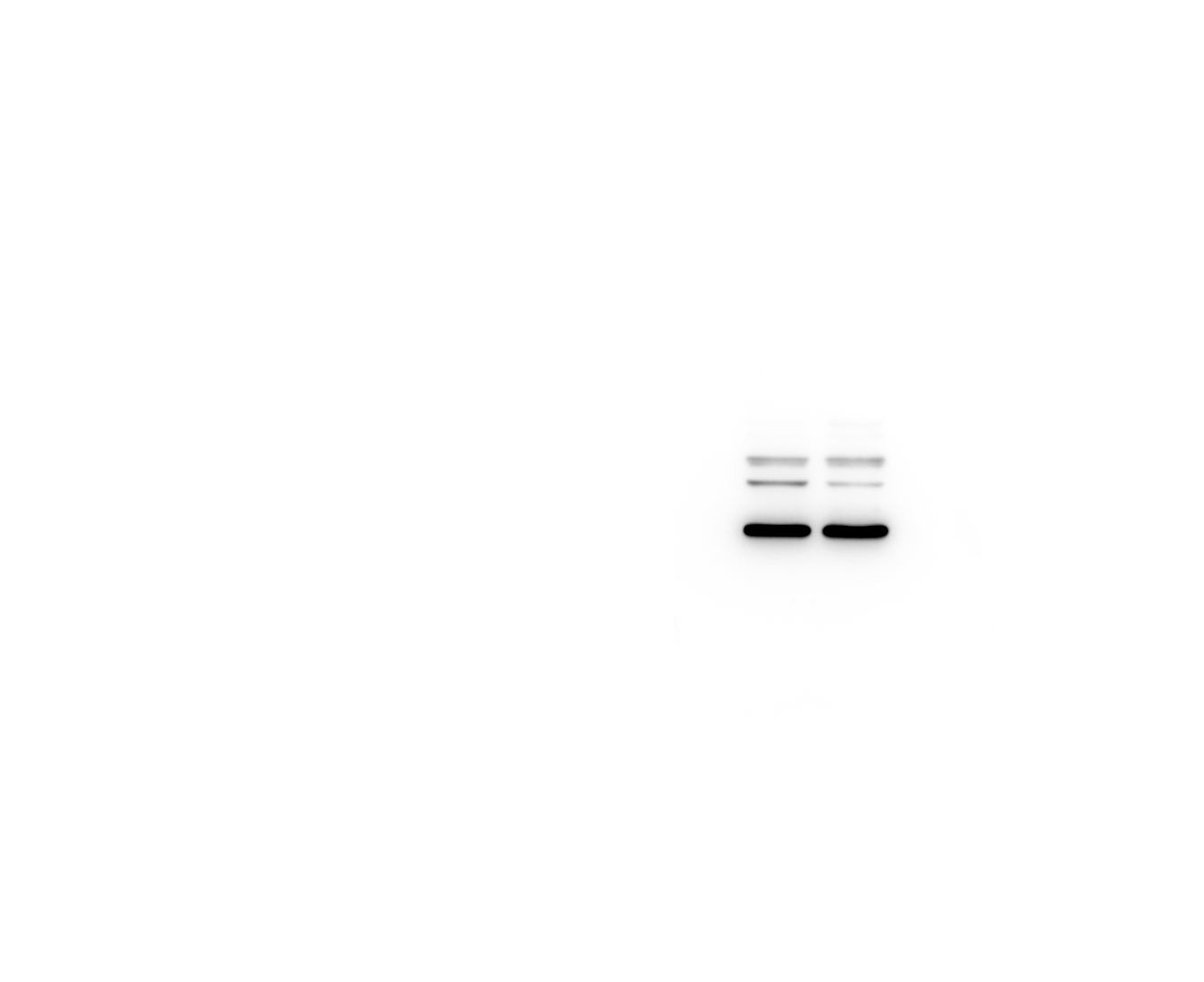

Supplement: Supplementary file 3 [file DataSheet3.zip › original.gel3/GAPDH 3.Tif]
